# Glycation modulates glutamatergic signaling and exacerbates Parkinson’s disease-like phenotypes

**DOI:** 10.1038/s41531-022-00314-x

**Published:** 2022-04-25

**Authors:** Ana Chegão, Mariana Guarda, Bruno M. Alexandre, Liana Shvachiy, Mariana Temido-Ferreira, Inês Marques-Morgado, Bárbara Fernandes Gomes, Rune Matthiesen, Luísa V. Lopes, Pedro R. Florindo, Ricardo A. Gomes, Patrícia Gomes-Alves, Joana E. Coelho, Tiago Fleming Outeiro, Hugo Vicente Miranda

**Affiliations:** 1 CEDOC, NOVA Medical School, NMS, Universidade NOVA de Lisboa, 1169-056 Lisboa, Portugal; 2 Instituto de Tecnologia Química e Biológica António Xavier, Universidade Nova de Lisboa, Av. Da República, 2780-157 Oeiras, Portugal; 3 iBET, Instituto de Biologia Experimental e Tecnológica, Apartado 12, 2781-901 Oeiras, Portugal; 4 Department of Experimental Neurodegeneration, Center for Biostructural Imaging of Neurodegeneration, University Medical Center Göttingen, Göttingen, Germany; 5 Instituto de Medicina Molecular João Lobo Antunes, Faculdade de Medicina da Universidade de Lisboa, Lisboa, Portugal; 6 Instituto de Investigação do Medicamento (iMed.ULisboa), Faculdade de Farmácia, Universidade de Lisboa, 1649-003 Lisboa, Portugal; 7 Max Planck Institute for Natural Sciences, 37075 Göttingen, Germany; 8 Faculty of Medical Sciences, Translational and Clinical Research Institute, Newcastle University, NE2 4HH Newcastle Upon Tyne, UK; 9 German Center for Neurodegenerative Diseases (DZNE), 37075 Göttingen, Germany

**Keywords:** Parkinson's disease, Neurophysiology, Systems biology, Proteins

## Abstract

Alpha-synuclein (aSyn) is a central player in the pathogenesis of synucleinopathies due to its accumulation in typical protein aggregates in the brain. However, it is still unclear how it contributes to neurodegeneration. Type-2 diabetes mellitus is a risk factor for Parkinson’s disease (PD). Interestingly, a common molecular alteration among these disorders is the age-associated increase in protein glycation. We hypothesized that glycation-induced neuronal dysfunction is a contributing factor in synucleinopathies. Here, we dissected the impact of methylglyoxal (MGO, a glycating agent) in mice overexpressing aSyn in the brain. We found that MGO-glycation potentiates motor, cognitive, olfactory, and colonic dysfunction in aSyn transgenic (Thy1-aSyn) mice that received a single dose of MGO via intracerebroventricular injection. aSyn accumulates in the midbrain, striatum, and prefrontal cortex, and protein glycation is increased in the cerebellum and midbrain. SWATH mass spectrometry analysis, used to quantify changes in the brain proteome, revealed that MGO mainly increase glutamatergic-associated proteins in the midbrain (NMDA, AMPA, glutaminase, VGLUT and EAAT1), but not in the prefrontal cortex, where it mainly affects the electron transport chain. The glycated proteins in the midbrain of MGO-injected Thy1-aSyn mice strongly correlate with PD and dopaminergic pathways. Overall, we demonstrated that MGO-induced glycation accelerates PD-like sensorimotor and cognitive alterations and suggest that the increase of glutamatergic signaling may underly these events. Our study sheds new light into the enhanced vulnerability of the midbrain in PD-related synaptic dysfunction and suggests that glycation suppressors and anti-glutamatergic drugs may hold promise as disease-modifying therapies for synucleinopathies.

## Introduction

Aging is an inevitable process that increases the risk for several conditions, including neurodegenerative disorders, such as Parkinson’s disease (PD). Neurodegenerative disorders are typically associated with the misfolding and aggregation of specific proteins in the brain and other tissues. However, the molecular mechanisms that trigger these phenomena are still elusive.

PD is one of several disorders known as synucleinopathies due to the misfolding and aggregation of alpha-synuclein (aSyn)^1,2^, a protein abundant in the brain that is also present in other tissues. Synucleinopathies also include dementia with Lewy bodies, multiple system atrophy, and pure autonomic failure^3–5^. Parkinsonism is a clinical syndrome characterized by resting tremor, bradykinesia, muscular rigidity, postural instability, and gait impairment^6–8^. Nevertheless, the clinical spectrum of PD includes several non-motor features such as cognitive impairment, hyposmia, obstipation, anxiety and depression, sleep disturbances, pain, and fatigue^7,9–11^.

aSyn is a natively unfolded protein that, under certain conditions, is prone to aggregation^2,12–15^. Several factors contribute to the oligomerization and fibrillization of aSyn, including high protein concentration, molecular crowding, mutations, posttranslational modifications, and interactions with specific metals and small molecules^16,17^.

PD is characterized by pronounced synaptic alterations, dysregulation of multiple neurotransmission pathways, including the loss of dopaminergic neurons in the *substantia nigra pars compacta* (SNpc), and by the presence of proteinaceous aggregates primarily composed of aSyn, known as Lewy bodies and Lewy neurites, in surviving neurons^1,18–21^. The pathological oligomerization and aggregation of aSyn is suggested to, somehow, trigger the degeneration of dopaminergic neurons in the SNpc^20–22^, and the consequent depletion of dopamine in the striatum induces alterations in dopamine signaling^23–26^ and major functional alterations in glutamatergic synapses^27–32^. Glutamate is the predominant excitatory neurotransmitter in the basal ganglia (BG)^33^. Although the striatum contains the highest density of glutamate receptors in the BG, glutamate or glutamate-dopamine neurons are intermixed with midbrain dopaminergic neurons. Interestingly, the SNpc is rich in N-methyl-D-aspartate (NMDA) and α-amino-3-hydroxy-5-methyl-4-isoxazolepropionic acid (AMPA) glutamate receptors, as well as in metabotropic glutamate receptors^34–36^. It is believed that the increase in firing of subthalamic nucleus neurons in PD acts as a compensatory mechanism to increase the release of dopamine from the surviving dopaminergic neurons in the SNpc in order to maintain dopamine homeostasis^37^. Furthermore, dopaminergic denervation also induces dysfunctional cortico-striatal glutamate release^38^, thereby eliciting an excitotoxic cascade that promotes further dopaminergic-neuronal loss and neurodegeneration^39–41^.

Since genetic alterations account for a smaller fraction of PD cases, it is imperative to identify and better understand the role of risk factors in the pathogenesis of this disorder. Type-2 diabetes mellitus, a widespread chronic metabolic disease, has been established as an important risk factor for PD and other neurodegenerative diseases^42,43^. Epidemiological studies revealed that 80% of PD patients have impaired glucose metabolism. Moreover, diabetes can increase the risk of developing PD in young diabetic individuals by up to 380%, and significantly accelerates the progression of both motor and cognitive deficits in PD patients^44–46^. However, the molecular mechanisms underlying this correlation are still unclear. One of the major outcomes of type-2 diabetes mellitus is the deleterious accumulation of reducing sugars that are unavoidably formed as by-products of essential metabolic processes such as glycolysis^47–50^. Glycation is a non-enzymatic reaction between reducing sugars, such as methylglyoxal (MGO), and biomolecules such as proteins, leading to the formation of mostly irreversible advanced glycation end products (AGEs)^49–51^. Importantly, the levels of circulating MGO in type-2 diabetes mellitus patients are 2–4-fold higher than in healthy individuals^52–55^.

Previously, we found that aSyn is glycated in the brain of PD patients and that this modification exacerbates aSyn pathogenicity by promoting its accumulation, oligomerization, aggregation, and toxicity, in vitro and in vivo^49,56,57^.

In this study, we aimed to determine whether generalized glycation in the mouse brain could trigger PD-like features, and to identify the molecular pathways implicated in this process. For this, we delivered MGO via intracerebroventricular (ICV) injection in transgenic Thy1-aSyn mice and in corresponding littermates and evaluated behavioral and biological alterations. We found that MGO exacerbates PD-like motor and non-motor features, alongside with proteomic alterations of glutamatergic components, specifically in the midbrain. In total, our study provides novel mechanistic insight into the connection between metabolic alterations, as those present in type-2 diabetes mellitus, and PD, opening novel avenues for the design of therapeutic interventions.

## Results

### MGO potentiates motor deficits and accelerates colonic dysfunction in Thy1-aSyn mice

To test our hypothesis that glycation-induced dysfunction of neuronal pathways might be an underlying molecular cause of synucleinopathies, we used Thy1-aSyn mice as a model of synucleinopathy. This model recapitulates several features of the PD spectrum, including aSyn accumulation, alterations in nigrostriatal dopaminergic pathway, and progressive motor and non-motor deficits^58,59^.

Briefly, age-matched male transgenic Thy1-aSyn and wild-type (WT) littermates mice received 5 µL of MGO (31.6 mM), corresponding to 0.16 µmol, or vehicle (phosphate-buffered solution - PBS, pH 7.4) through ICV injection into the right lateral ventricle. Animals were allowed to recover from the procedure. Three weeks post injection, mice were weighed, handled, and the Shirpa protocol performed. Behavioral phenotyping was performed 4 weeks after surgery (Supplementary Fig. 1a–c).

First, to establish a baseline, we applied a battery of motor tests to characterize the motor behavior of the animals used in the study (20-week-old animals). The open field test was performed to evaluate general motor activity, gross locomotor activity, and exploration habits^60^. The vertical pole test and rotarod were aimed at assessing locomotor activity, motor coordination and balance^60–63^. To evaluate balance and grip strength, the wire hang test was conducted^60,64^. The adhesive removal test was performed to evaluate sensorimotor deficits related to the paw and the mouth^65^. Finally, we also assessed the motor function using SHIRPA protocol tests, mainly the hindlimb clasping test, and colonic function (assessed during open field test)^61,66^.

**Fig. 1:**
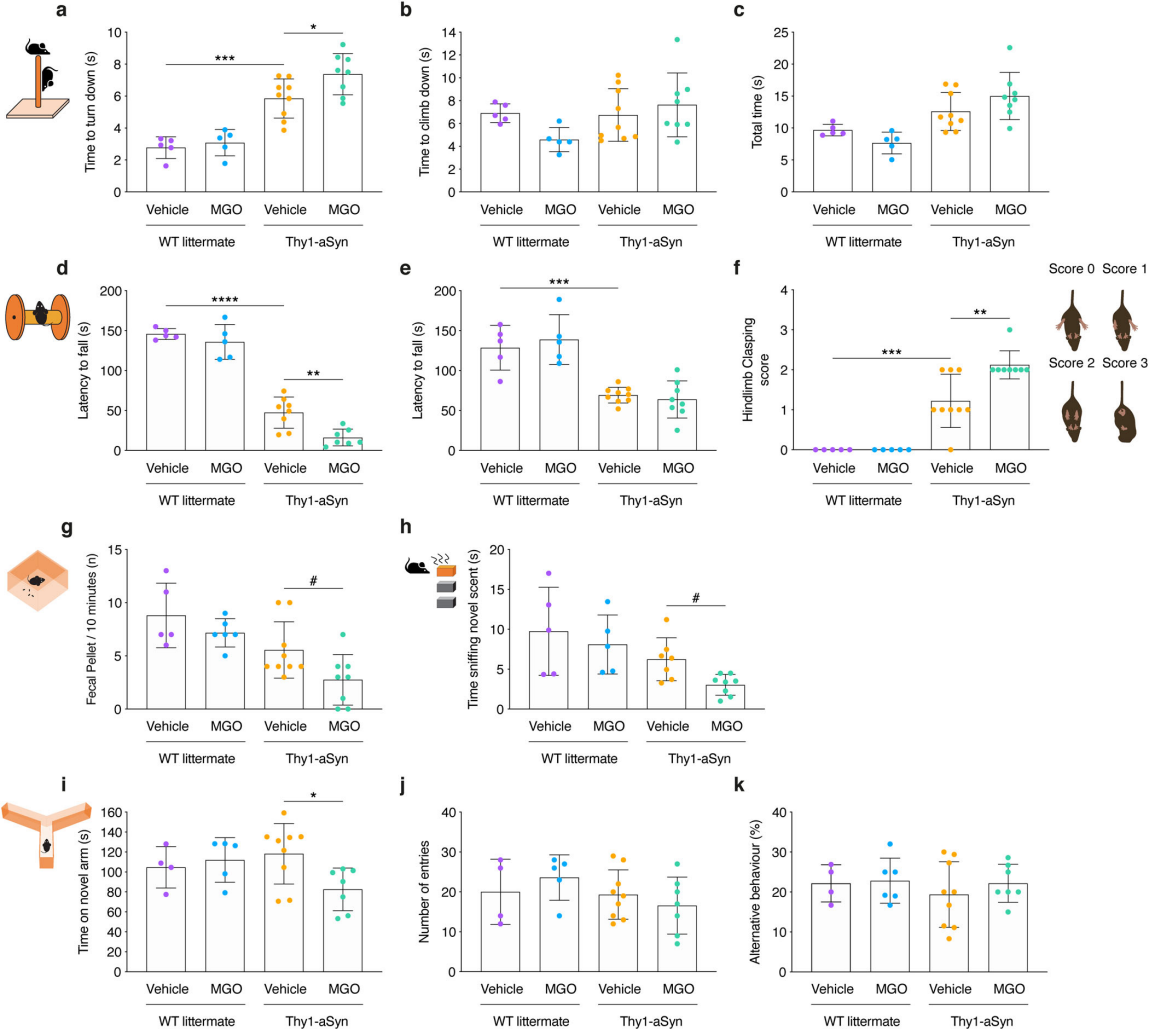
MGO-treatment induces motor dysfunction, cognitive impairment, olfactory, and colonic disturbances in Thy1-aSyn mice. Wild-type littermate (WT) and Thy1-aSyn transgenic (Tg) mice received an intracerebroventricular (ICV) injection of MGO or vehicle (PBS) at 16 weeks of age. Behavioral testing started 4 weeks post surgery. Plot representations of: pole test—**a** time to turn down, **b** time to climb down, **c** total time; rotarod—**d** stationary protocol, **e** acceleration protocol; **f** hindlimb clasping score; **g** fecal pellet production; block test—**h** time sniffing novel scent; Y maze test—**i** time on novel arm, **j** number of entries, **k** alternative behavior. At least *n* = 5 in all groups, data in all panels are average, with error bars representing standard deviation, Ordinary one-way ANOVA, *p < 0.05, **p < 0.01, ***p < 0.001, ****p < 0.0001; unpaired two-tailed *t*-test with equal SD, ^#^*p* < 0.05.

Vehicle-injected transgenic Thy1-aSyn mice required more time to turn down on the vertical pole (2.1-fold increase) than WT littermates but displayed no significant differences in the time of climbing down or in the total time to perform the task (Fig. 1a–c). Thy1-aSyn mice also showed a decreased latency to fall in the rotarod at both stationary (3.1-fold decrease) or accelerated rotation (1.9-fold decrease) (Fig. 1d, e) when compared to WT littermates. Impairments were also apparent in grip strength evaluated in the wire hang test, with a decreased latency to fall (3.1-fold decrease) (Supplementary Fig. 1d), and in the hindlimb clasping test, showing increased score when compared to WT littermate mice (Fig. 1f). We observed no sensorimotor differences, in the adhesive removal test (Supplementary Fig. 1e), no alterations of locomotor activity and exploration habits in the open field (Supplementary Fig. 1f–j), and no differences in colonic function, given by the number of fecal pellets dropped in an open field arena in 10 min (Fig. 1g).

Next, we assessed the effect of MGO injection and found that Thy1-aSyn mice required more time to turn down on the vertical pole compared to vehicle-injected Thy1-aSyn mice (1.3-fold increase) (Fig. 1a). No changes in the time to climb down or in the total time to perform the task were observed (Fig. 1b, c). Rotarod performance was also worse in MGO-injected Thy1-aSyn mice, evaluated at a steady rotation of the rod (2.9-fold decrease of latency to fall), when compared to vehicle-injected Thy1-aSyn mice (Fig. 1d). At accelerated rotation, no differences were observed (Fig. 1e). Finally, MGO-injected Thy1-mice showed worse performance in the hindlimb clasping test (Fig. 1f), and worse colonic function, with a smaller number of fecal pellets produced, when comparing with vehicle-injected Thy1-mice (Fig. 1g). On the other hand, MGO treatment did not significantly change the grip strength (wire hang test, Supplementary Fig. 1d), sensorimotor function (adhesive test Supplementary Fig. 1e), or locomotor activity and exploration habits (open field test, Supplementary Fig. 1f–j) when compared to vehicle-injected Thy1-aSyn mice. MGO treatment in WT littermates had no effect in all behavioral tests performed (Fig. 1a–k and Supplementary Fig. 1d–l).

### MGO aggravates cognitive and olfactory disturbances in Thy1-aSyn mice

Next, we assessed the occurrence of PD-associated non-motor features in the different animal groups^7,9–11^. The Y maze test was performed to assess short-term spatial reference memory, as a read-out of cognitive function^67,68^. Anxiety-related behavior was evaluated using the elevated plus maze test^69,70^. Olfactory function was assessed using the block test that evaluates sensitivity to social smells, olfactory acuity, and discrimination^71,72^.

At 20 weeks, Thy1-aSyn mice did not show alterations in the block test (Fig. 1h), Y maze test (Fig. 1i–k), and elevated plus maze (Supplementary Fig. 1k, l) when compared to WT littermates. Likewise, MGO injection did not alter the performance of WT mice in these tests (Fig. 1h–k and Supplementary Fig. 1f–l). In contrast, MGO-injected Thy1-aSyn mice spent less time sniffing the novel scent (2-fold decrease), comparing to vehicle-injected Thy1-aSyn mice (Fig. 1h). In addition, MGO-injected Thy1-aSyn mice spent less time (1.4-fold decrease) in the novel arm in the Y maze test (Fig. 1i), comparing to vehicle-injected Thy1-aSyn mice. No differences were observed in anxiety-related behavior (Supplementary Fig. 1k, l).

Altogether, we observed that MGO exacerbates motor deficits and triggers or anticipates colonic, cognitive, and olfactory disturbances in aSyn-overexpressing mice. We also confirmed that 20-week-old Thy1-aSyn mice already display impaired motor performance, without colonic, cognitive or olfactory disturbances, compared to WT littermate animals.

### MGO increases the levels of aSyn, AGEs, pS129-aSyn and aSyn insolubility in the midbrain of Thy1-aSyn mice

**Fig. 2:**
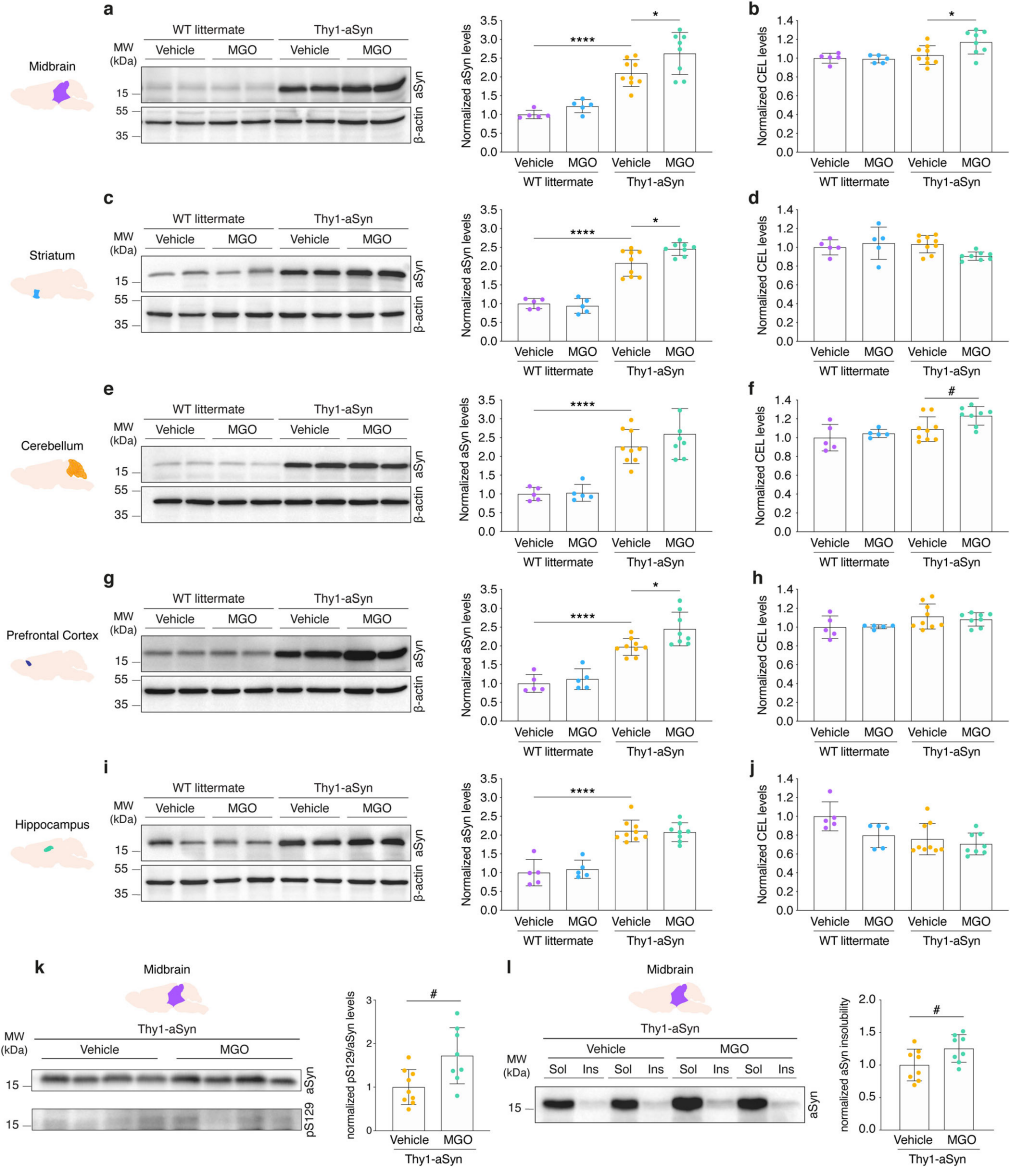
MGO-treatment induces the accumulation of aSyn and AGEs in the midbrain. Wild-type littermate (WT) and Thy1-aSyn transgenic mice received an intracerebroventricular (ICV) injection of MGO or vehicle (PBS) and protein brain extracts from several regions analyzed 5 weeks post injection. Protein extracts were resolved by SDS-PAGE or loaded into membranes in a dot-blot system (see Supplementary Fig. 2). Membranes were probed with anti-aSyn, anti-CEL, anti-pS129-aSyn and anti-β-actin for normalization. Representative blots showing two samples from each experimental group are shown for aSyn, and densitometric analysis represented for aSyn and CEL in **a**, **b** midbrain, **c**, **d** striatum, **e**, **f** cerebellum, **g**, **h** prefrontal cortex, and **i**, **j** hippocampus, respectively. **k** Representative blot showing four samples from vehicle- and MGO-injected Thy1-aSyn mice are shown for pS129-aSyn and aSyn total signal. Densitometric analysis of the ratio between pS129-aSyn and aSyn is presented. **l** Representative blot showing aSyn probing in soluble and insoluble fraction from vehicle- and MGO-injected Thy1-aSyn mice. Densitometric analysis of the ratio between the insoluble and the sum of both soluble and insoluble signals is presented. At least *n* = 5 in all groups, data in all panels are average with error bars representing standard deviation, Ordinary one-way ANOVA, *p < 0.05, **p < 0.01, ****p < 0.0001; unpaired two-tailed *t*-test with equal SD, ^#^*p* < 0.05.

After the behavioral analyses, animals were sacrificed, and the brains were analyzed. The left hemisphere was collected and dissected into midbrain, striatum, cerebellum, prefrontal cortex, and hippocampus, and these regions were analyzed by immunoblotting. Protein extracts were probed for aSyn and β-actin, for normalization. Interestingly, MGO-injected Thy1-aSyn mice presented higher levels of aSyn in the midbrain (1.2-fold increase) (Fig. 2a), striatum (1.2-fold increase) (Fig. 2c), and prefrontal cortex (1.2-fold increase) (Fig. 2i) in comparison to vehicle-injected Thy1-aSyn mice.

To understand the possible sustained effects of protein glycation, we measured the levels of N^ε^-carboxyethyl lysine (CEL), an MGO-derived AGE (MAGE), by dot-blot analysis, followed by immunoblotting probing for CEL and β-actin for normalization. Comparing to vehicle-injected Thy1-aSyn, MGO-injected Thy1-aSyn mice showed higher levels of CEL in the midbrain and cerebellum (both with 1.1-fold increase) (Fig. 2b, f and Supplementary Fig. 2). Strikingly, the hippocampus displayed decreased levels of CEL in both vehicle- and MGO-injected Thy1-aSyn mice (1.3- and 1.4-fold decrease, respectively) in comparison to vehicle-injected WT animals, suggesting that this region might be protected from glycation (Fig. 2h). No alterations in the levels of glyoxalase 1, the most important MGO-detoxification pathway, are detected between the experimental groups (Supplementary Fig. 3).

As expected, Thy1-aSyn mice presented a 2.1-fold increase in the levels of aSyn in the midbrain, striatum, and hippocampus, and 1.8- and 2.3-fold increase in the prefrontal cortex and cerebellum, respectively, in comparison to WT littermates (Fig. 2a, c, e, g, i). MGO-injected WT mice had no differences in the levels of aSyn or CEL in comparison to vehicle-injected WT mice (Fig. 2a–j).

As a measurement of aSyn pathology, we measured the levels of pS129-aSyn and assessed aSyn solubility in Triton X-100 in the midbrain. Notably, MGO-injected Thy1-aSyn mice displayed higher pS129-aSyn levels (1.7-fold) (Fig. 2k) and increased insolubility of aSyn (1.25-fold) in this brain region (Fig. 2l).

### MGO triggers neuronal loss in the vicinity of the midbrain of Thy1-aSyn mice

**Fig. 3:**
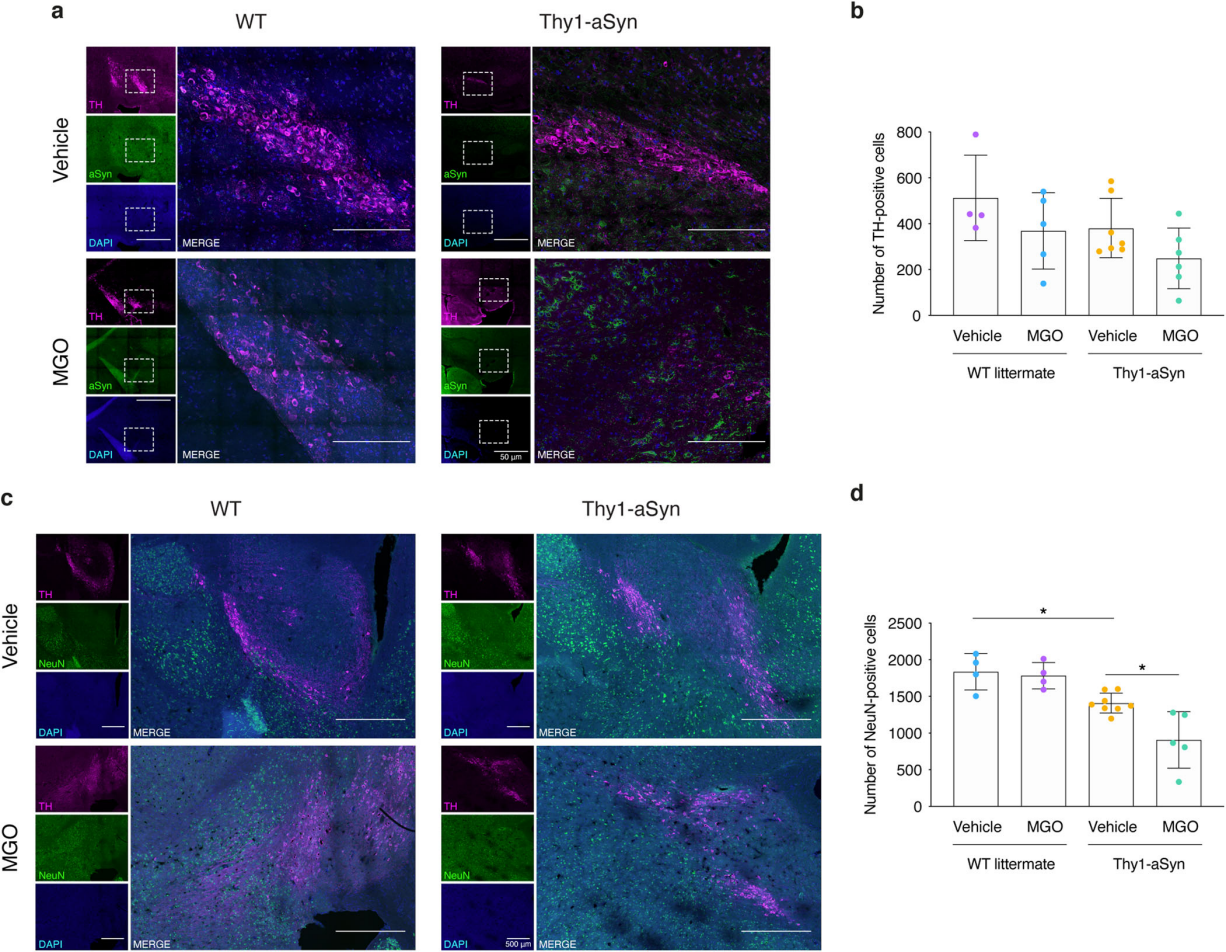
MGO-treatment induces the loss of NeuN-positive neurons in the SNpc. Wild-type littermate (WT) and Thy1-aSyn transgenic mice received an intracerebroventricular (ICV) injection of MGO or vehicle (PBS). **a** Representative micrographs of brain sections immunostained for TH (magenta), aSyn (green), and DAPI (blue). Merge signal is shown. Scale bar = 50 μm. Depicted area of *substantia nigra* (dashed square) is shown. Scale bar = 200 μm. **b** The number of TH-positive cells per experimental group is shown. **c** Representative micrographs of brain sections of the *substantia nigra* immunostained for TH (magenta), NeuN (green), and DAPI (blue). Merge signal is shown. Scale bar = 500 μm. **d** The number of NeuN-positive cells per experimental group is shown. At least *n* = 4 in all groups, data in all panels are average with error bars representing standard deviation, ordinary one-way ANOVA, *p < 0.05.

Next, we compared the number of dopaminergic neurons and of general neuronal population at SNpc surrounding area via immunohistochemical analysis. No alteration in the number of TH-positive neurons was detected between experimental groups. (Fig. 3a, b). In contrast, MGO-injected Thy1-aSyn mice showed lower number of NeuN-positive neurons, when compared to vehicle-injected Thy1-aSyn mice (1.6-fold decrease) (Fig. 3c, d). These findings suggest that although MGO treatment does not induce selective dopaminergic-neuronal loss in the SNpc, the loss of non-dopaminergic cells is observed in MGO-injected Thy1-aSyn mice.

### Proteomic analysis of differentially regulated proteins

To identify the pathways and molecular mechanisms that are dysregulated upon MGO injection, we performed a proteomic analysis using total protein extracts from the midbrain and prefrontal cortex of five animals per group (Thy1-aSyn and WT mice injected with vehicle or MGO). Sequential window acquisition of all theoretical mass spectrometry (SWATH-MS) was performed, and the MS data matched with a customized library of MS/MS spectra created from LC-ESI-MS analysis of brain protein extracts of animals from each group. Each of the five samples per group was analyzed three times. A total of 2153 proteins were identified for a peptide confidence level of >99%, FDR threshold of 1%. For the quantitative analysis, an initial outlier analysis was performed. The points removed were then replaced with mean values of the analyzed group, and intensity-based absolute quantification (iBAQ) was normalized. ANOVA analysis between the experimental groups of each brain region was performed to identify the differently regulated proteins.

The response to MGO was followed by comparing MGO-injected with vehicle-injected animals. In the midbrain, 457 proteins were found to be differently regulated between vehicle-injected and MGO-injected WT mice, while 350 proteins were differently present between vehicle-injected and MGO-injected Thy1-aSyn mice. In the prefrontal cortex, 206 proteins were found to be altered by MGO injection in WT mice, and 172 in Thy1-aSyn mice.

The comparison between vehicle-injected Thy1-aSyn and WT mice, revealed the response of the brain proteome to the overexpression of aSyn. Interestingly, we found that 454 proteins were altered in the midbrain, and 256 proteins were altered in the prefrontal cortex of aSyn-overexpressing animals.

#### Proteins uniquely altered by MGO in aSyn transgenic animals

**Fig. 4:**
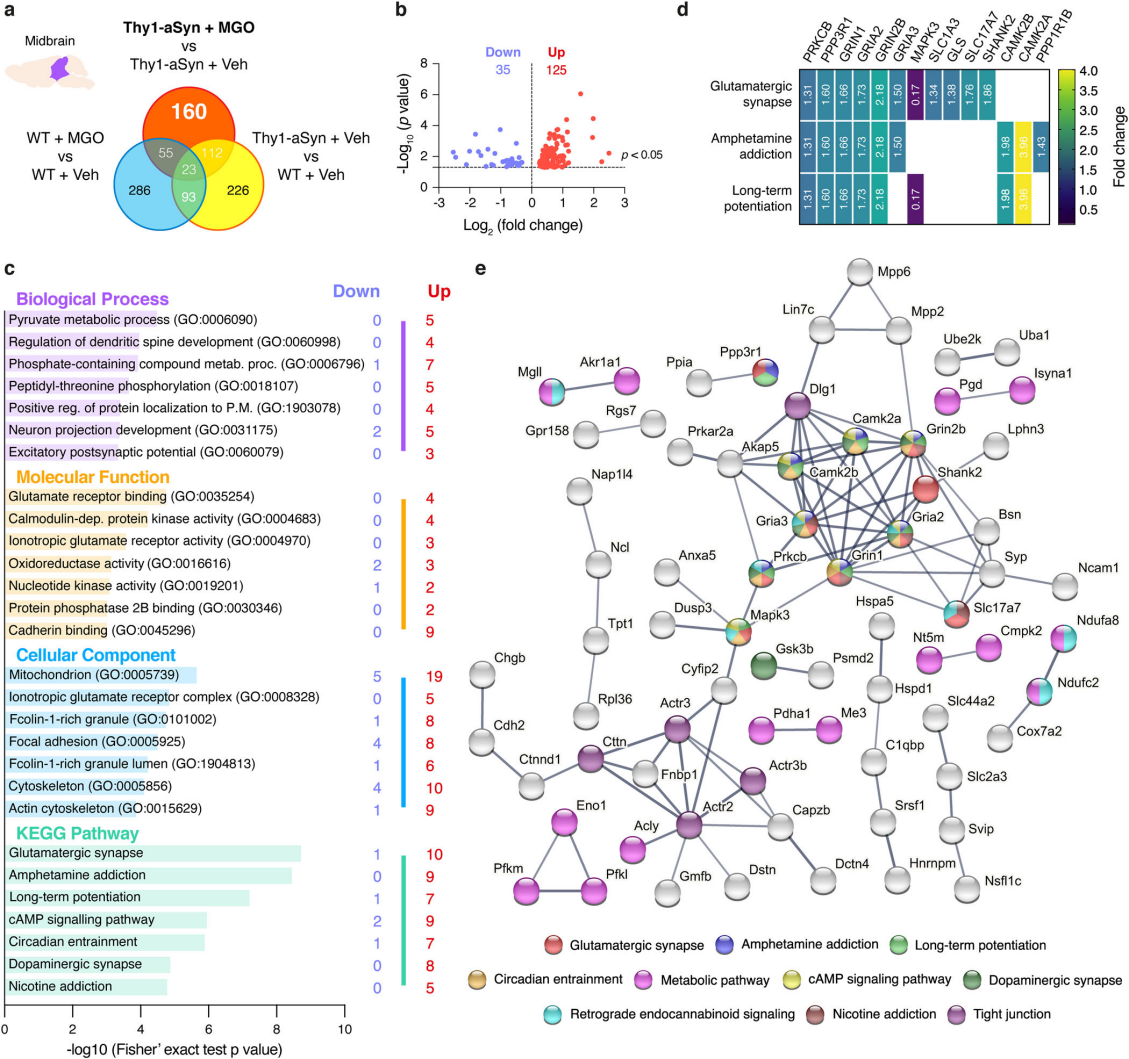
MGO-treatment alters components of the glutamatergic pathway in the midbrain of Thy1-aSyn mice. Proteomic differences between midbrain proteins from WT or Thy1-aSyn mice injected with vehicle or MGO. **a** Venn diagram depicting unique and shared midbrain proteome between pairwise comparisons of Thy1-aSyn mice injected with MGO and vehicle, Thy1-aSyn mice and WT mice both injected with vehicle and WT mice injected with MGO and vehicle. **b** Volcano plot for unique hits of Thy1-aSyn mice injected with MGO and Vehicle. Statistically significant down- and upregulated hits are presented. **c** KEEG pathways and Gene Ontology (GO) terms analysis is presented, depicting the number of down- and upregulated unique hits. Distribution of −log_10_ (Fisher exact test *p* value) is shown. **d** Heatmap of the uniquely altered proteins corresponding to the top 3 altered KEGG pathways. **e** Protein–protein interaction networks of uniquely altered proteins, extracted from the STRING 11.0 database. Only the proteins that are interacting within a network are show. KEGG pathways or GO Biological processes are color coded.

**Fig. 5:**
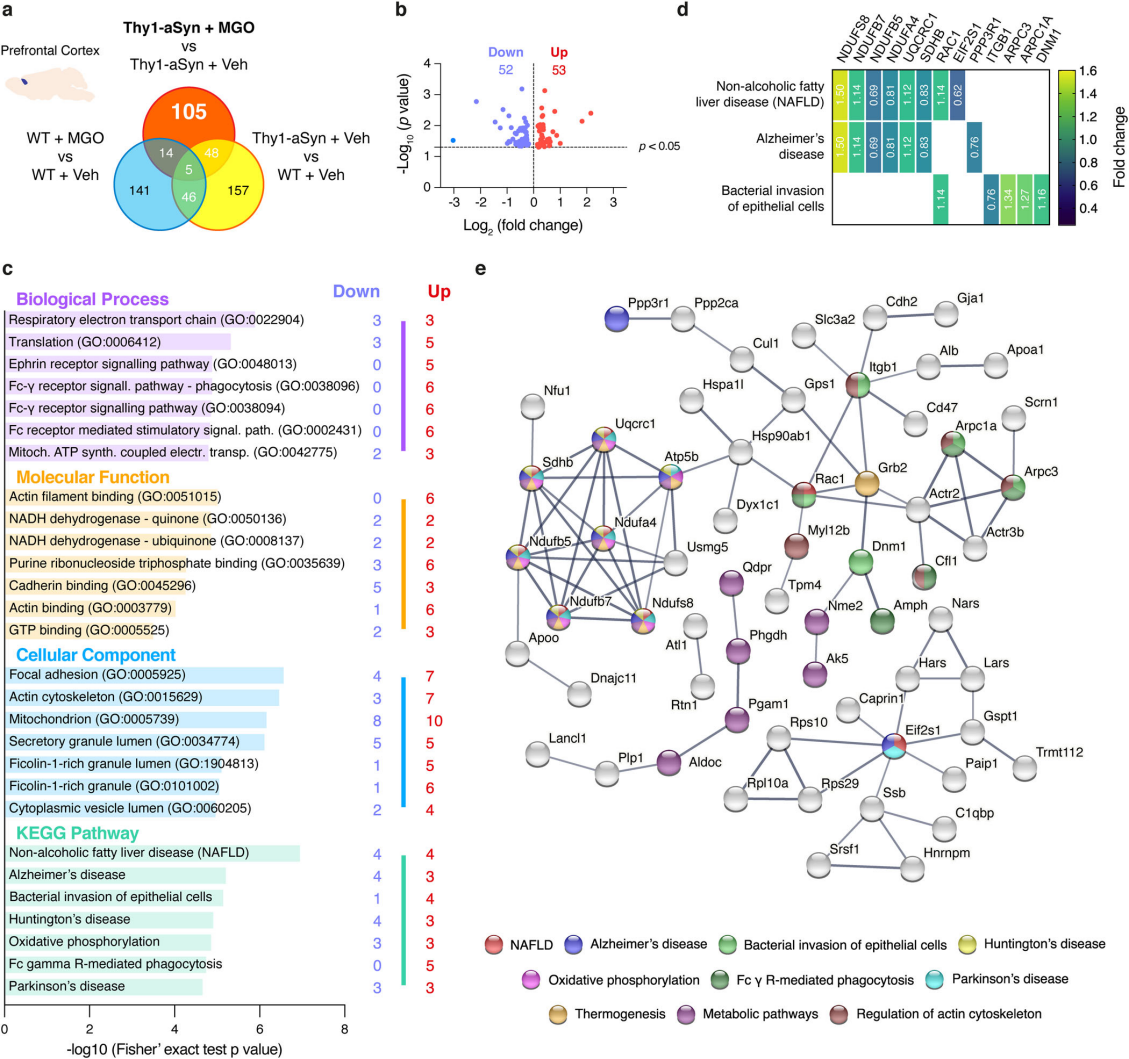
MGO-treatment alters components of the respiratory electron transport chain in the prefrontal cortex of Thy1-aSyn mice. Proteomic differences between cortical proteins from WT or Thy1-aSyn mice injected with vehicle or MGO. **a** Venn diagram depicting unique and shared midbrain proteome between pairwise comparisons of Thy1-aSyn mice injected with MGO and vehicle, Thy1-aSyn mice and WT mice both injected with vehicle and WT mice injected with MGO and vehicle. **b** Volcano plot for unique hits of Thy1-aSyn mice injected with MGO and Vehicle. Statistically significant down- and upregulated hits are presented. **c** KEEG pathways and Gene Ontology (GO) terms analysis is presented, depicting the number of down- and upregulated unique hits. Distribution of −log_10_ (Fisher exact test *p* value) is shown. **d** Heatmap of the uniquely altered proteins corresponding to the top 3 altered KEGG pathways. **e** Protein–protein interaction networks of uniquely altered proteins, extracted from the STRING 11.0 database. Only the proteins that are interacting within a network are show. KEGG pathways or GO Biological processes are color coded.

Next, we identified targets specifically induced by MGO. To this end, we used a Venn diagram analysis representing the comparisons that enabled us to exclude the general effects of MGO (WT mice injected with MGO vs vehicle) and of aSyn overexpression (vehicle-injected Thy1-aSyn vs WT mice), as presented for midbrain (Fig. 4a) and prefrontal cortex (Fig. 5a). We found that 160 target proteins were altered as a specific response to MGO treatment in the midbrain, and 105 in the prefrontal cortex of Thy1-aSyn mice. From these, 125 proteins are upregulated, and 35 proteins are downregulated in the midbrain, whereas in the prefrontal cortex 53 proteins are upregulated while 52 are downregulated (Figs. 4b and 5b). By comparing the specific response in the midbrain and prefrontal cortex, we found that only 18 proteins were commonly affected in both regions, suggesting that MGO elicited region-specific alterations (Supplementary Table 3).

#### MGO induces alterations in glutamatergic system-related proteins in the midbrain of Thy1-aSyn mice

Upon functional enrichment analysis, we found that the glutamatergic synapse Kyoto Encyclopedia of Genes and Genomes (KEGG) pathway was mostly affected by MGO in the midbrain of Thy1-aSyn mice. This was followed by amphetamine addiction and long-term potentiation. Strikingly, these three pathways share several common proteins (Fig. 4c, d). We found that MGO mainly induced the increase in the levels of proteins related with glutamate production, transport, and its respective transporters and receptors (Fig. 4d). Consistently with the changes identified in glutamatergic synapses, the most affected Gene Ontology (GO) molecular function was the glutamate receptor binding, and the second most affected GO cellular component was the ionotropic glutamate receptor complex (Fig. 4c). The major changes in GO biological processes were in pyruvate metabolic processes and in the regulation of dendritic spine development (Fig. 4c).

STRING analysis showed a strong connection between the uniquely altered proteins. A central cluster of highly connected proteins included calcium/calmodulin-dependent protein kinase type II alpha (CAMK2A), and beta (CAMK2B), NMDA (GRIN1, GRIN2B), AMPA (GRIA2, GRIA3), SH3 and multiple ankyrin repeat domains protein 2 (SHANK2) and protein kinase C beta type (PRKCB). Interestingly, other interconnected proteins were associated with tight junction or with metabolic pathway (KEGG) (Fig. 4e).

#### MGO alters components of the respiratory electron transport chain in the prefrontal cortex of Thy1-aSyn mice

In the prefrontal cortex, the most affected KEGG pathways included non-alcoholic fatty liver disease (NAFLD), Alzheimer’s disease, and bacterial invasion of epithelial cells pathways (Fig. 5c, d). Strikingly, the hits related to NAFLD, Alzheimer’s disease, Huntington’s disease, oxidative phosphorylation, and PD, corresponded to a group of proteins involved in the respiratory electron transport chain (Fig. 5c–e).

Other unique hits and interconnected proteins were associated with actin filament binding, purine ribonucleoside triphosphate binding, cadherin binding processes, Fc γ receptor mediated phagocytosis or to metabolic pathway (Fig. 5c, e).

#### MGO induces alterations in components of oxidative phosphorylation and PD pathways in WT mice

**Fig. 6:**
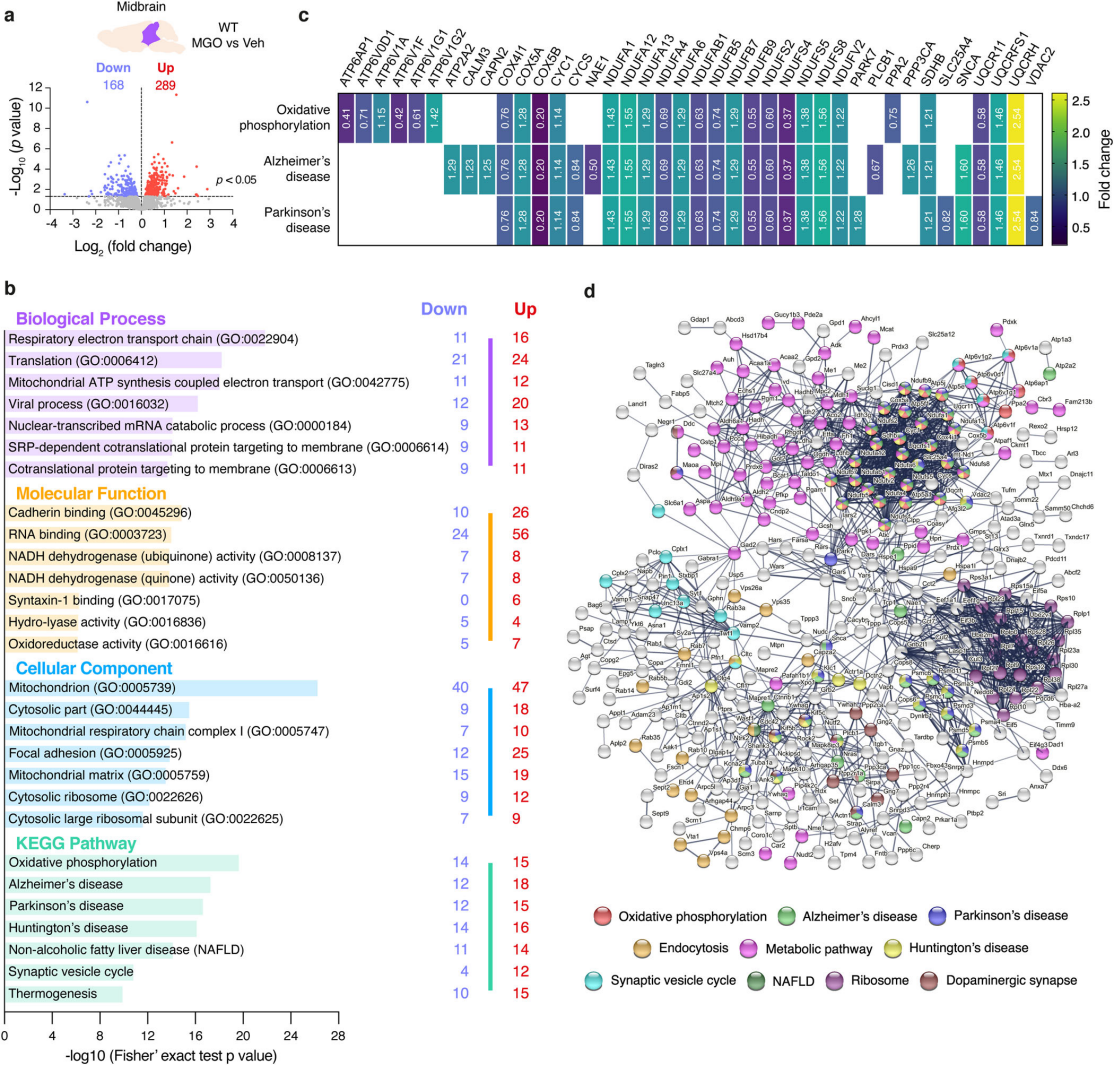
MGO-treatment alters respiratory electron transport chain and neurodegenerative-associated components in the midbrain of WT mice. Proteomic differences between midbrain proteins from WT mice injected with vehicle or MGO. **a** Volcano plot for presenting statistically significant down- and upregulated hits. **b** KEEG pathways and Gene Ontology (GO) terms analysis is presented, depicting the number of down- and upregulated unique hits. Distribution of −log_10_ (Fisher exact test *p* value) is shown. **c** Heatmap of the uniquely altered proteins corresponding to the top 3 altered KEGG pathways. **d** Protein–protein interaction networks of the altered proteins, extracted from the STRING 11.0 database. Only the proteins that are interacting within a network are show. KEGG pathways or GO Biological processes are color coded.

**Fig. 7:**
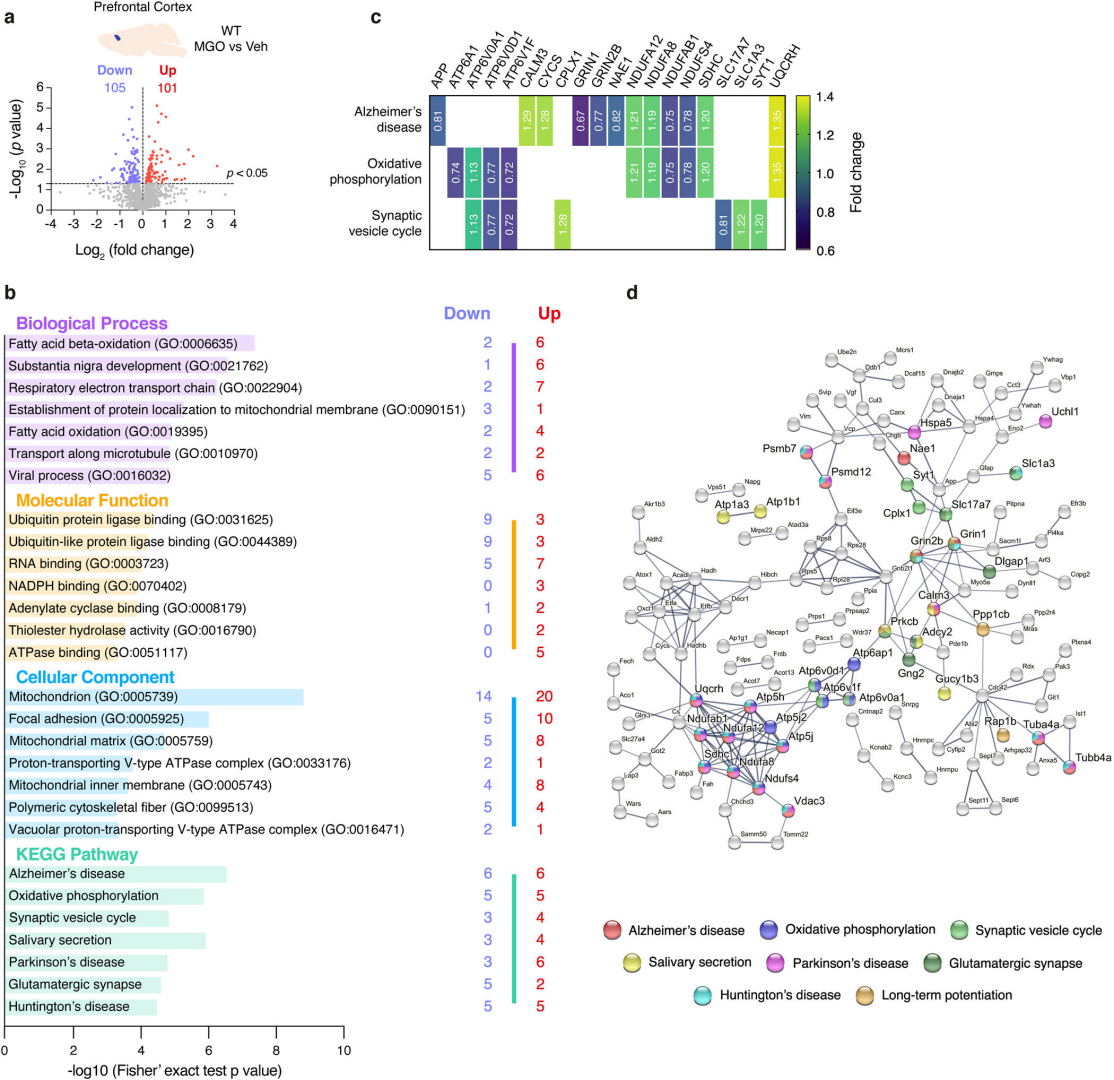
MGO-treatment alters oxidative phosphorylation-associated components in the prefrontal cortex of WT mice. Proteomic differences between prefrontal cortex proteins from WT mice injected with vehicle or MGO. **a** Volcano plot for presenting statistically significant down- and upregulated hits. **b** KEEG pathways and Gene Ontology (GO) terms analysis is presented, depicting the number of down- and upregulated unique hits. Distribution of −log_10_ (Fisher exact test *p* value) is shown. **c** Heatmap of the uniquely altered proteins corresponding to the top 3 altered KEGG pathways. **d** Protein–protein interaction networks of the altered proteins, extracted from the STRING 11.0 database. Only the proteins that are interacting within a network are show. KEGG pathways or GO Biological processes are color coded.

MGO injection in WT mice altered 457 proteins in the midbrain (289 upregulated and 168 downregulated) (Fig. 6a and Supplementary Table 4) and 206 proteins in the prefrontal cortex (101 upregulated and 105 downregulated) (Fig. 7a and Supplementary Table 5).

Functional enrichment analysis showed that oxidative phosphorylation was the pathway most affected by MGO in the midbrain of WT mice (Fig. 6b–d). Moreover, neurodegeneration-associated pathways were also affected, including components from Alzheimer’s, Parkinson’s and Huntington’s diseases. Most of these proteins were shared with the alterations identified in oxidative phosphorylation pathway (Fig. 6b–d). Notably, synaptic vesicle cycle and dopaminergic synapse components were also altered (Fig. 6d).

Consistently with the changes identified in the KEGG pathways, the most affected GO biological process and cellular component were the respiratory electron chain and the mitochondrion respectively (Fig. 6b). The major changes in GO molecular function were in cadherin binding and in NADH dehydrogenase ubiquinone and quinone activity, members of the respiratory complex I (Fig. 6b).

Alterations in components of dopaminergic signaling were also detected in the midbrain of MGO-injected WT mice. Major alterations occur at postsynaptic level, increasing calmodulin 3 (CALM3), protein phosphatase 3 catalytic subunit alpha (PPP3CA), protein phosphatase 1 catalytic subunit gamma (PPP1CC), protein phosphatase 2 scaffold subunit alpha (PPP2R1A) levels, while decreasing G protein subunit gamma 2 and 7 (GNG2 and GNG7). At a presynaptic level, monoamine oxidase A (MAOA) decreases while dopa decarboxylase (DDC) increases.

In the prefrontal cortex, MGO mostly affected Alzheimer’s disease, oxidative phosphorylation, synaptic vesicle cycle, PD, and glutamatergic synapse KEGG pathways (Fig. 7b–d). In agreement with these alterations, the most affected GO cellular component was the mitochondrion (Fig. 7b). Moreover, fatty acid beta-oxidation, *substantia nigra* development, and respiratory electron transport chain are the most affected GO biological processes. Interestingly, ubiquitin or ubiquitin-like protein ligase binding were the most altered GO molecular functions (Fig. 7b).

STRING analysis showed a strong cluster including the altered targets from oxidative phosphorylation and neurodegeneration-associated pathways. Interestingly, other interconnected proteins were associated with synaptic vesicle cycle, and glutamatergic synapse (Fig. 7d).

#### MGO induces similar alterations to aSyn overexpression in components of Parkinson’s disease and oxidative phosphorylation pathways

**Fig. 8:**
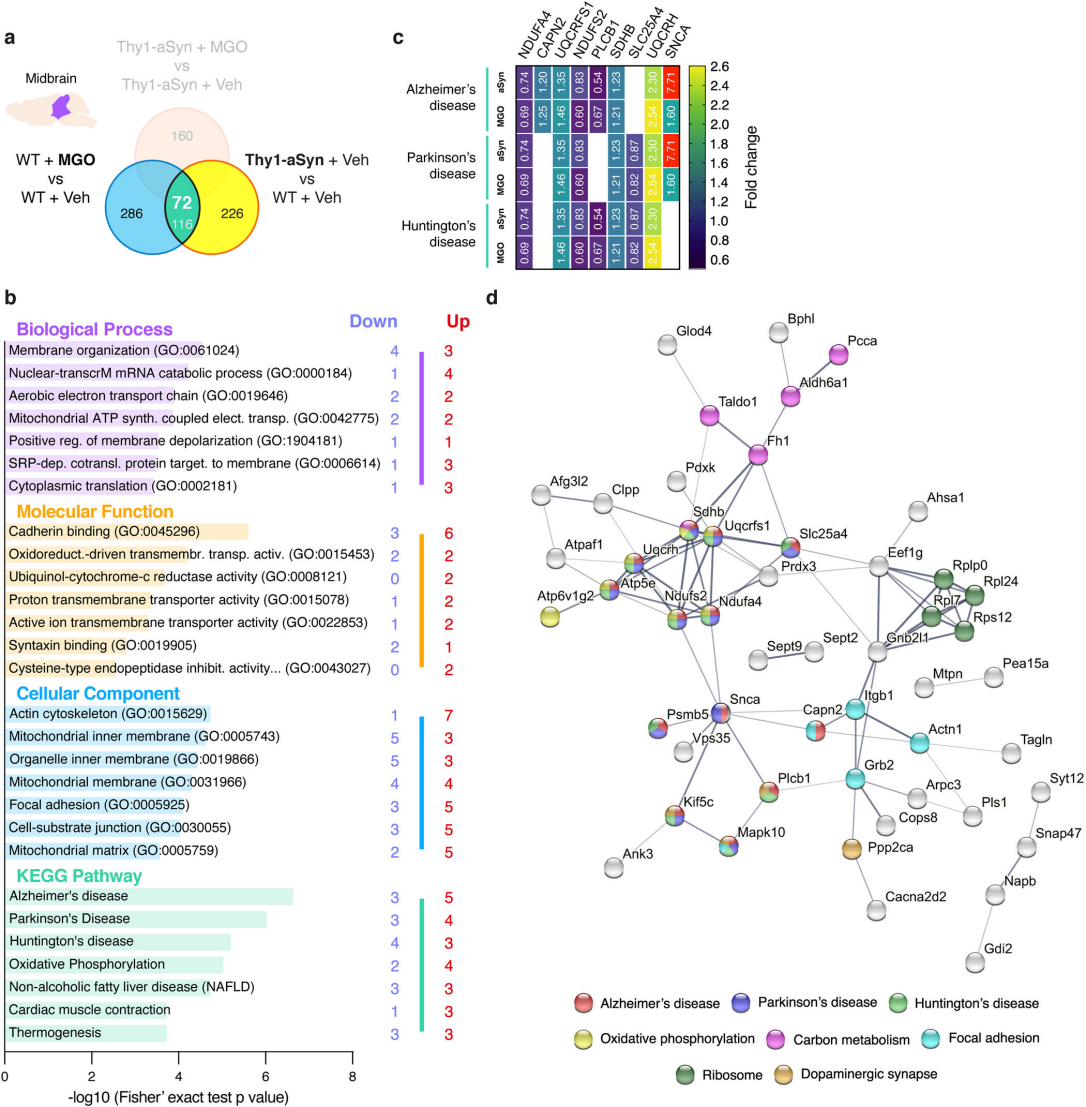
MGO-treatment and aSyn overexpression similarly alters neurodegenerative- and oxidative phosphorylation-associated components in the midbrain. Proteomic differences between midbrain proteins from WT or Thy1-aSyn mice injected with vehicle or MGO. **a** Venn diagram depicting midbrain proteome alterations between pairwise comparisons of MGO vs Vehicle-injected WT mice and Thy1-aSyn vs WT mice both injected with vehicle. From the identified 116 proteins, 72 are altered in the same direction (up- or downregulated). **b** KEEG pathways and Gene Ontology (GO) terms analysis is presented for the commonly altered proteins, depicting the number of down- and upregulated unique hits. Distribution of −log_10_ (Fisher exact test *p* value) is shown. **c** Heatmap of the commonly altered proteins corresponding to the top 3 altered KEGG pathways is presented per each pairwise comparison (aSyn—vehicle-Thy1-aSyn vs vehicle-WT; MGO—MGO-WT vs Veh-MGO). **d** Protein–protein interaction networks of the commonly altered proteins, extracted from the STRING 11.0 database. Only the proteins that are interacting within a network are show. KEGG pathways or GO Biological processes are color coded.

Next, we identified the targets that were similarly altered by MGO challenge or aSyn overexpression. To this end, we used comparisons that enabled us to determine the common hits induced by MGO (WT mice injected with MGO vs vehicle) and by aSyn overexpression (vehicle-injected Thy1-aSyn vs WT mice) (Fig. 8a). From the 454 proteins altered by aSyn and the 457 by MGO, 116 proteins were altered in both groups. From these, 72 proteins showed the same trend of alteration (up- or downregulated) (Supplementary Table 6).

Functional analysis showed that MGO and aSyn mostly affected components associated with the KEGG pathways Alzheimer’s, Parkinson’s, and Huntington’s diseases, as well as oxidative phosphorylation (Fig. 8b–d). In agreement, membrane organization and aerobic electron transport chain, as well as molecular functions associated with oxidative phosphorylation, and cadherin binding were the most affected GO biological processes and molecular functions. Actin cytoskeleton and mitochondrial inner membrane are the most affected GO cellular components (Fig. 8b–d). Interestingly, the extent of alteration induced by aSyn and MGO were similar between the components associated with the top 3 KEGG pathways (Fig. 8c).

#### Glycation mainly occurs in components associated with Parkinson’s disease and dopaminergic synapses

**Fig. 9:**
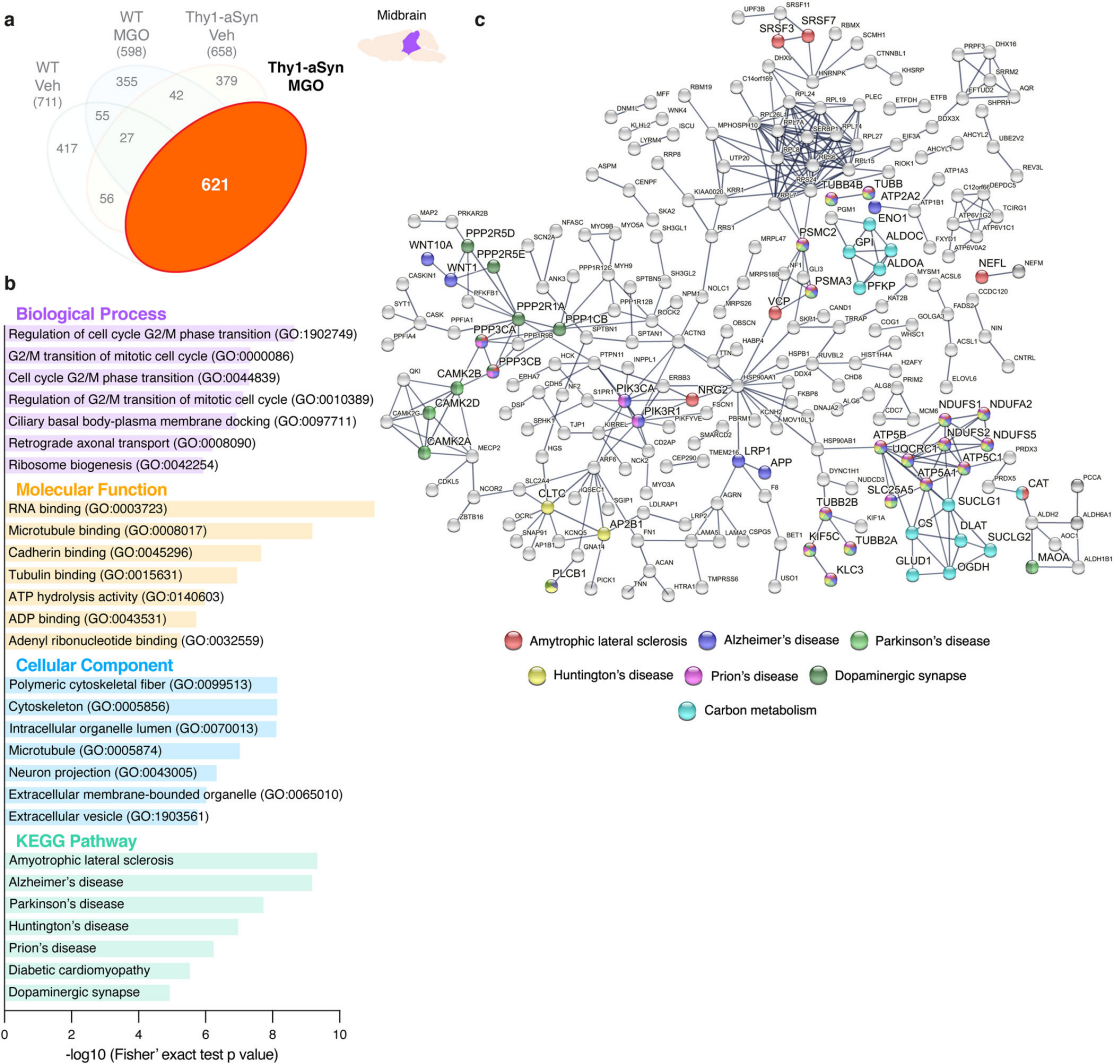
Glycation mainly impacts neurodegenerative- and dopaminergic synapse-associated pathways in the midbrain of MGO-injected Thy1-aSyn mice. Glycation profile of midbrain proteins from the different experimental groups. **a** Venn diagram depicting glycated proteins (621) in MGO-injected Thy1-aSyn mice. **b** KEEG pathways and Gene Ontology (GO) terms analysis is presented for the glycated proteins in MGO-injected Thy1-aSyn mice. Distribution of −log_10_ (Fisher exact test *p* value) is shown. **c** Protein–protein interaction networks of the glycated proteins in MGO-injected Thy1-aSyn mice, extracted from the STRING 11.0 database. Only the proteins that are interacting within a network are show. KEGG pathways or GO Biological processes are color coded.

Using proteomics approach, we determined which proteins were glycated in the midbrain proteome of all experimental groups. In contrast to the quantitative analyses described above, a limitation of this approach is that it only allows a qualitative analysis of the glycated proteins. A peptide search toward MAGEs including CEL, argpyrimidine, hydroimidazolones and tetrahidropirimidine was performed. A total of 711 (vehicle-injected WT mice), 598 (MGO-injected WT mice), 658 (vehicle-injected Thy1-aSyn mice) and 621 (MGO-injected Thy1-aSyn mice) glycated proteins were detected (Fig. 9a).

The functional analysis of the glycated proteome in MGO-injected Thy1-aSyn mice showed that several glycated proteins correlate better with neurodegeneration-associated pathways, including PD, and dopaminergic synapse (Fig. 9b, c and Supplementary Table 7).

We identified 354 glycated proteins that were uniquely identified in MGO-injected Thy1-aSyn mice (Supplementary Fig. 4a). Interestingly, these also correlated better with pathways of neurodegeneration as PD (Supplementary Fig. 4b, c). Proteins associated with proteasomal (*Psmc2*, *Psma3*; *Usp35*, *Ube3d*, *Usp17l2*) and autophagy (*Atg2b*) clearance; mitochondrial function (*Ndufa2* and *Uqcrc1*); heat-shock protein 27 (HSPB1); and insulin signaling, and cell survival (*Pik3r1* and *Pik3ca*) were uniquely glycated in this group (Supplementary Table 8).

## Discussion

Synucleinopathies are a group of devastating neurodegenerative diseases for which disease-modifying therapies are missing. The misfolding, accumulation, and aggregation of aSyn, a common hallmark among the various synucleinopathies, is thought to be a key event in the neurodegenerative process. However, our poor understanding of the molecular mechanisms underlying neurodegeneration has hampered our ability to develop effective therapies. Mutations and polymorphisms have been linked to familiar forms of PD^73,74^, but account for only 5–10% of cases^75,76^, suggesting that an interplay between genetics and environmental factors account for the vast majority of cases.

Type-2 diabetes mellitus has emerged as an important risk factor for various neurodegenerative disorders and, in particular, for PD^42–44,46,49,50^. Epidemiological studies revealed a strong association between type-2 diabetes mellitus and the risk for developing PD^44^. Importantly, type-2 diabetes mellitus accelerates the progression of both motor and cognitive deficits in PD patients^44–46^. However, the molecular mechanisms underlying the connection between the two diseases remains poorly understood. Type-2 diabetes mellitus is a chronic metabolic disease known for glucose metabolism imbalance, and is characterized by hyperglycemia, insulin resistance, and impaired glucose tolerance^45,47,77,78^. Remarkably, glycation, a major molecular outcome of type-2 diabetes mellitus, has also been implicated in several neurodegenerative disorders. We found that glycation modulates the pathogenicity of both aSyn and huntingtin, central players in PD and in Huntington’s disease, respectively^56,79^. Moreover, several studies showed that glycation also plays a role in Alzheimer’s disease^80–82^. Given that protein aggregation is a common hallmark among different neurodegenerative disorders, we posit that glycation may modulate the aggregation and toxicity of the different aggregation-prone proteins and that it may unveil novel targets for therapeutic intervention.

In this study, we investigated how glycation contributes to the dysfunction of neuronal pathways implicated in synucleinopathies, and how it contributes to the onset of PD-like phenotypes. To explore our hypothesis, 16-week-old Thy1-aSyn or WT littermate mice received a single dose of 160 nmol of MGO or vehicle via ICV injection. In a previous study, we evaluated the impact of an acute delivery of MGO directly to the SNpc or to the striatum^56^. Although several aSyn-associated phenotypes were identified, our previous experimental procedure was based on the delivery of MGO directly into the brain regions that are mostly affected in PD. Here, we applied more physiological amount of MGO (4.5-times lower than previously) and used ICV injection to take advantage of the CSF route for the direct delivery of compounds to the central nervous system, allowing MGO diffusion throughout the brain and was based on previously reported studies^83–89^. Since our aim was to disclose the role of MGO-induced glycation specifically in the brain, we choose the ICV route instead of a systemic administration of MGO by intraperitoneal injection or oral solution. The deleterious impact of glycation in multiorgan is well established as well as its role in the pathophysiology of diabetes complications that significantly increase the associated comorbidities^47^. To unveil the specific role of MGO-induced glycation in the brain and its impact on motor function and cognition, ICV injection avoids peripheral effects that could mislead our results. The amount of MGO used corresponds to reported levels of this glycating agent in the mouse brain (approximately 60–130 nmol/brain)^90^. Importantly, the amount used is significantly lower (4 to more than 100 times) than those used in other studies using ICV delivery^87–90^.

We performed a detailed behavioral characterization of the animals 4 weeks after ICV injection, and 5 weeks post injection animals were sacrificed and brains were collected for biochemical, immunohistochemical and SWATH-MS analysis. The choice of this timeline was based on the reported onset of behavioral alterations in Thy1-aSyn mice^58^, and our previously published work in the herein used animal facility^91^. Moreover, we conducted a pilot study to optimize both the timeline and the amount of MGO injected.

Previous reports indicate that most PD-like behavioral features in Thy1-aSyn mice start at the age of 28 weeks^58^, although they may also exhibit earlier motor deficits^92^. In our experimental conditions, 20-week-old Thy1-aSyn mice already presented reduced performance on the vertical pole test, rotarod and wire hang test, and increased hindlimb clasping score^92^. At this stage, no colonic, cognitive, olfactory, and anxiety-related alterations were observed. Therefore, our cohort displayed several alterations that are typically observed at 28 weeks. Moreover, these Thy1-aSyn mice already presented a higher motor impairment in the rotarod test than usual (24 months of age)^93^. In contrast, although Thy1-aSyn mice are reported to be more anxious and to present olfactory alterations at 12 weeks of age, we did not confirm these features in our experimental cohort^71,94^. In agreement with previous reports, no cognitive deficits were observed on the Y maze test, as they commonly appear in mice between 28 and 36 weeks of age^94^.

Following the protocol described, we found that MGO injection in Thy1-aSyn mice aggravates motor deficits as observed in the vertical pole test and hindlimb clasping test. In the rotarod test, we found that MGO injection in Thy1-aSyn mice induced a significant impairment in the constant speed protocol that is a stress test aimed at evaluating psychical and motor resistance. In contrast, we did not find any differences in the acceleration protocol that mostly evaluates capacity to respond to a challenging task. Moreover, MGO injection accelerates cognitive impairment in Thy1-aSyn mice, as this was only described to occur between the age of 28 and 36 weeks^94^. It also anticipated the onset of colonic and olfactory disturbances in Thy1-aSyn mice, assessed in the open field and block test, respectively. These behavioral alterations suggest that MGO accelerates and aggravates disease progression in aSyn-overexpressing mice. In the experimental conditions used, although some tendencies are observed, MGO treatment did not alter the behavioral phenotypes of WT mice. In the future, it will be of interest to design a study to assess the effect of chronic exposure to MGO in WT animals, to test whether glycation triggers PD-like phenotypes.

Our biochemical analysis of different brain regions showed that MGO-injected Thy1-aSyn mice display increased levels of aSyn in the midbrain, striatum, and prefrontal cortex. Consistently with the MGO-associated potentiation of PD-like phenotypes, we also found higher levels of S129 phosphorylation, and higher levels of insoluble aSyn in the midbrain, which may account for the behavioral alterations observed^95–97^. Chemicals delivered via ICV injection reach various brain regions, including the prefrontal cortex, striatum, hypothalamus, hippocampus, midbrain, and medulla^98^. Although the local concentration of the injected chemical may differ from region to region, the areas surrounding the ventricles should be exposed to higher concentrations of the chemicals, in contrast to subarachnoid areas. Therefore, it was surprising that the midbrain and cerebellum displayed a significant increase of MGO-glycated proteins. Since the striatum and hippocampus should also be exposed to MGO, this finding suggests that either the proteostasis network in the midbrain is less effective in clearing dysfunctional glycated proteins than other brain regions, or that the midbrain is more susceptible to carbonyl-stress. To explore this, we measured the levels of glyoxalase 1, the most important basal defense mechanism against carbonyl-stress by detoxifying MGO. While MGO does not elicit alterations in the levels of this enzyme, it is apparent that midbrain presents higher levels. This finding suggests that an upregulation of glyoxalase 1 in this brain region may be a compensatory mechanism to cope with increased levels of MGO, further suggesting an increased vulnerability of this brain region to glycation. It is relevant to state that proteome analyses were performed 5 weeks post-MGO injection, a timing that, depending on the degradative activity of each target cells, should allow natural protein turn-over to take place and to clear abnormally modified proteins, preventing their accumulation.

Regarding the assessment of neurodegeneration, while the number of TH-positive neurons is not altered upon MGO-treatment, a decrease of NeuN-positive neurons in the SNpc of MGO-injected Thy1-aSyn mice in comparison to vehicle-injected Thy1-aSyn mice occurs. This finding may suggest that the observed motor phenotype could be independent from dopaminergic neuronal loss, resulting instead from the dysregulation of multiple neurotransmission pathways. Moreover, we cannot discard that the function of these neurons may be compromised and account for the observed decrease in motor performance^27,29–31^. In fact, TH-positive neuronal loss is not expected in Thy1-aSyn mice at the age of 5 month, as the number of neurons remains unaltered until the age of 24 month in animals that present several PD-like phenotypes^58^.

Using our proteomics approach, we determined that the response to MGO-injection differs between prefrontal cortex and midbrain. Noteworthy, this approach is quantitative and highly sensitive, allowing to determine small statistical differences in the levels of the measured proteins. From the uniquely dysregulated proteins specifically induced by glycation in Thy1-aSyn mice, only 18 proteins are commonly dysregulated between the prefrontal cortex and the midbrain. Moreover, the impact of MGO in the midbrain affects a larger number of proteins (350) than in the prefrontal cortex (172), for the same total amount of detected proteins. Although MGO increased the levels of aSyn in both regions, the differences may be due to a distinct exposure to MGO, as AGEs accumulated in the midbrain, but not in the prefrontal cortex. Another possibility is that this may also be the result of a higher susceptibility of midbrain cells to MGO, as previously suggested. Given the high number of protein targets and the differences across brain region, it is evident that the effects of MGO are pleotropic and depend on the different vulnerability of target brain areas.

MGO glycation is known to trigger the unfolded protein response, impair oxidative metabolism, drive mitochondrial dysfunction, and impact on the oxidative stress response^49,50,99,100^. Several of these consequences are intrinsically related with the depletion of cellular defenses against oxidative stress since reduced glutathione and NADPH are shared cofactors to cope with both oxidative stress response and with the detoxification of MGO by glyoxalases and aldose reductases^49,50^. This is particularly relevant in neurons, which are more vulnerable to MGO due to their lower capacity to detoxify this compound, particularly when compared to astrocytes^101,102^. Failure in its detoxification results in increased glycation of several proteins, particularly in the mitochondria^103^. In fact, neuronal mitochondrial damage is well established, and is known to suppress oxygen consumption, decrease the activity of respiratory chain complexes, and the capacity of energy production, increasing the production of reactive oxygen species^104,105^. Importantly, mitochondrial dysfunction in the dopaminergic neurons is associated with PD^106,107^. Thy1-aSyn mice injected with MGO displayed severe dysregulation of oxidative phosphorylation components in the prefrontal cortex. Several elements of mitochondrial complex I were dysregulated, with a decrease of *Ndufb5* and increase of both *Ndufb7* and *Ndufb8* . Moreover, components of complex II (*Sdhb*) and complex IV (*Ndufa4*) were decreased, while a component of complex III (*Uqcrc1*) was increased. These findings suggest that increased levels of MGO in the brain may contribute to mitochondrial dysfunction in the cortical area.

**Fig. 10:**
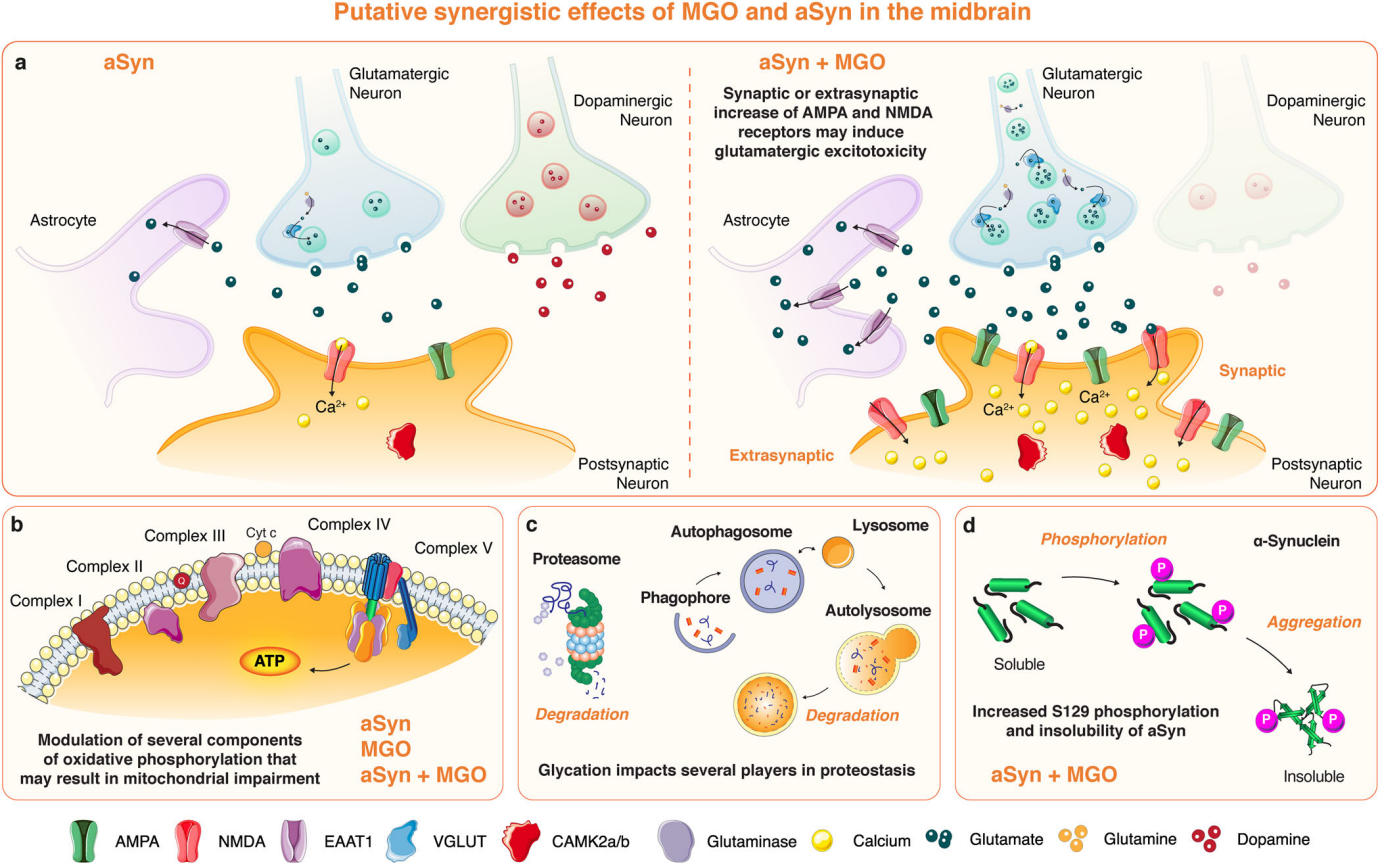
Putative synergistic effects of MGO and aSyn in the midbrain. **a** The schematic depicts the MGO-altered glutamatergic proteins in the midbrain of Thy1-aSyn mice. In contrast to control, vehicle-injected mice (left), MGO-injected mice (right) display signs of altered dopaminergic and glutamatergic signaling. Proteomics data suggest a probable increase of glutamate production (glutaminase) and packaging in glutamatergic vesicles (VGLUT), the release of glutamate to the synaptic cleft and astrocytic reuptake (EAAT1) or entry to the postsynaptic neuron via AMPA or NMDA receptors. The increase in these receptors may occur at the synapse or extrasynaptically, inducing excessive calcium entry to the cell. An increase of both CAMK2A and CAMK2B is also observed, supporting the hypothesis of enhanced glutamatergic signaling. A strong imbalance in the postsynaptic levels of calcium can occur and contribute to excitotoxicity. **b** Proteomics data also suggest alterations in several components of the electron transport chain in the oxidative phosphorylation pathway. Are detected upon aSyn overexpression (Veh-Thy1-aSyn vs Veh-WT mice), after MGO injection (Veh-WT vs MGO-WT) or in MGO-injected Thy1-aSyn (vs Veh-Thy1-aSyn), suggesting that MGO and aSyn trigger the dysfunction of this common pathway. In fact, some of these targets are glycated in MGO-injected Thy1-aSyn mice. **c** The glycated proteome also impacts on proteostasis components such as the proteasome, autophagy-lysosome pathway, and heat-shock proteins response. **d** MGO further increases aSyn S129 phosphorylation and aSyn insolubility, typical hallmarks of aSyn pathology.

Altered glutamatergic firing has been described in PD and is believed to be caused by the dopamine depletion in the striatum^27–31^. The SNpc is highly rich in NMDA, AMPA and metabotropic glutamate receptors, and glutamate or glutamate-dopamine neurons are intermixed with midbrain dopaminergic neurons^34–36^. In the midbrain of MGO-injected Thy1-aSyn mice, we observed a generalized loss of neurons. Moreover, we found that several uniquely dysregulated proteins in the midbrain of MGO-injected mice belong to the glutamatergic pathway, which may reflect a possible increase of glutamatergic signaling. Specifically, there was an increase of the levels of glutaminase and vesicular glutamate transporter 1 (VGLUT1). These events suggest a probable increase in the presynaptic production of glutamate, and of its vesicular storage (Fig. 10a). The levels of astrocytic excitatory amino acid transporter 1 (EAAT1) were also increased and this protein is classically responsible for the rapid removal of released glutamate from the synaptic cleft^108,109^. This finding further supports the hypothesis that glutamate production and release are increased in this brain region upon MGO insult. Moreover, a generalized increase of AMPA (GRIA2 and GRIA3) and NMDA (GRIN1 and GRIN2B) receptors was observed, most probably at the postsynaptic neuron (Fig. 10a). We observed an increase of the GRIN2B, a subunit of NMDA receptor that is generally linked to cell death signaling^110,111^, and is typically more abundant early in development, shifting to GRIN2A during development^111^. Our study does not allow us to resolve whether the increased levels of these glutamate receptor subunits are at the postsynaptic membrane, extrasynaptically, or at the cytosol. Nevertheless, several postsynaptic glutamatergic targets are also increased, including the calcineurin subunit B type 1, protein phosphatase 1 regulatory subunit 1B (PPP1R1B), PRKCB, SHANK2, suggesting increased glutamatergic activity. In fact, we also found an increase in the levels of CAMK2A and CAMK2B in MGO-injected Thy1-aSyn mice, which are known to promote the trafficking and transient translocation of AMPA receptors to the postsynaptic membrane^112^. Together with the observation of decreased levels of mitogen-activated protein kinase 3 (MAPK3/ERK2), these findings suggest an impairment of long-term potentiation (LTP)^113^, as we previously reported^112^. Excessive release of glutamate may also activate extrasynaptic glutamate receptors. Oligomeric aSyn, may not only induce increased release of glutamate from astrocytes, but also activate extrasynaptic NMDA receptors, and this process may lead to synaptic loss. Importantly, the specific extrasynaptic NMDA receptor antagonist NitroSynapsin protects from these deleterious effects of oligomerized aSyn^114^. Independently of the localization of glutamate receptors, excessive glutamate may trigger apoptotic cell death because of intracellular calcium overload upon receptor stimulation, and is a common hallmark of neurodegenerative diseases^115^.

Based on our findings, we cannot distinguish whether the alteration in glutamatergic signaling precedes dopaminergic degeneration or is a consequence of dopaminergic dysregulation in MGO-injected Thy1-aSyn mice that raises two hypotheses. In the first, MGO may elicit glutamatergic hyperactivity, causing an excitotoxic phenomenon that triggers general neuronal degeneration. Our results suggest that exacerbated behavioral phenotype might result from the dysregulation of multiple neurotransmission pathways in the midbrain, rather than the exclusive failure of the dopaminergic system. In agreement with this hypothesis, we observed the accumulation of aSyn, of S129 phosphorylated aSyn and higher amount of aSyn in the insoluble fraction in the midbrain, in accordance with our previous report that MGO-treatment increases both aSyn glycation and oligomerization^56^. Moreover, we further reported that prolonged exposure to aSyn oligomers drives to the long-lasting increase of basal glutamatergic synaptic transmission^112^. In fact, aSyn oligomers may activate NMDA receptors, increasing the basal levels of intracellular calcium and recruiting AMPA receptors to the membrane^112^. For the second hypothesis, in response to MGO deleterious effects, the brain may also trigger glutamatergic firing in the midbrain as a compensatory mechanism to enhance the release of dopamine from the surviving dopaminergic neurons and to maintain dopamine homeostasis. Importantly, alterations in glutamate content in the brain of PD patients have been reported by several clinical studies using magnetic resonance imaging, positron emission tomography and single photon emission computed tomography, consistent with increased glutamate neurotransmission^116–118^. Moreover, increased levels of glutamate in the plasma of PD patients have been reported, further reflecting increased cerebral glutamatergic activity^119,120^. Alterations in this glutamatergic transmission contribute to the pathophysiology of dyskinesias, impaired motor coordination and motor fluctuations^28,121–125^. In addition, dysfunction of glutamate metabolism is also implicated in non-motor features of PD, including depression and cognitive impairment^126–128^. However, it is presently unclear if the glutamatergic alterations cause or exacerbate neurodegeneration in patients with PD. In fact, glutamatergic hyperactivity may prompt an excitotoxic cascade further promoting neurodegeneration^37,115^, which would ultimately lead to the observed mice behavioral alterations.

Although MGO-injected WT littermates do not present major phenotypic alterations under our experimental procedure, we observed interesting alterations in their proteome both in the midbrain and prefrontal cortex. MGO alters oxidative phosphorylation players both in the midbrain and prefrontal cortex. These include several components of complex I (*Nduf* family), Complex II (*Sdhb*), Complex III (*Uqcr* family), Complex IV (*Ndufa4*) and ATP synthase (*Atp5* family). Several of these proteins commonly associated with PD-associated pathways. Importantly, mitochondrial dysfunction is suggested as a first player in the neurodegenerative process. These findings suggest that alterations in oxidative phosphorylation represent the acute effects of MGO-treatment in the brain proteome. Remarkably, MGO also impacts proteins involved in dopaminergic signaling in the midbrain.

Several components of neurodegenerative-associated pathways are commonly altered by MGO challenge or upon aSyn overexpression (MGO vs Vehicle-injected WT mice, or Thy1-aSyn vs WT mice, both injected with vehicle). While components of complex I (*Ndufs2* and *Ndufa4*) are decreased, components of complex II (*Sdhb*) and complex III (*Uqcrh* and *Uqcrfs1*) increase, most likely as a compensatory mechanism to complex I failure. aSyn, for example, is increased 1.6 times upon MGO challenge, confirming our western-blot analysis, a feature of PD. Loss-of-function *VPS35* mutations were identified in autosomal-dominant PD patients^129^, a protein with reduced levels in the SNpc^130^ that causes dopaminergic neuronal loss^131^. Upon MGO challenge or aSyn overexpression, a decrease in VPS35 levels (0.8-fold) is detected, which may suggest increased pathology.

In depth analysis of the glycated peptides sequenced via SWATH-MS, reveals that most of the glycated proteins correlate with PD pathway. They are components of the mitochondrial complexes of oxidative phosphorylation, and players in dopaminergic synapse pathways. These include MAOA, involved in the breakdown of dopamine, and the family of CAMK2 (A, B, D, G), with important role in dopaminergic signaling and mobilization of synaptic vesicles^132^. For example, CAMK2A is known to bind to and control the activity of both dopamine transporter^133^ and of dopamine D2 receptors^134^. Notably, these kinases also play an important role in glutamatergic signaling, particularly dependent of NMDA stimulation, impacting memory and synaptic plasticity^132^. Several regulatory subunits of protein phosphatase 2 (PP2) are also glycated. Dysregulation of PP2A with B55α subunit may contribute for the hyperphosphorylation of aSyn since it is a major phosphatase of pS129-aSyn^135,136^. In agreement, an increase of pS129-aSyn in the midbrain of MGO-injected Thy1-aSyn mice was observed. Several proteins involved in carbon metabolism also present MAGEs. A central core of these proteins corresponds to members of the glycolytic pathway involved in the metabolism of the triose phosphates, precursors of MGO formation^50^. This may suggest an alteration in MGO formation rate, as we previously analyzed^137,138^. A second core corresponds to the citric acid cycle and catalase, important players in energy production and in the detoxification of hydrogen peroxide.

The exclusively glycated proteome in MGO-injected Thy1-aSyn mice suggests that the protein quality control systems may be affected. In particular, the proteasomal- and autophagy-associated proteins (*Psmc2/3*, *Usp35*, *Ube3d*, *Usp17l2*, *Atg2b*), which are vital players in protein clearance mechanisms that are highly compromised in PD. In agreement, we previously observed that MGO blocks aSyn clearance by impairing both the proteasome and the autophagy-lysosome pathway^56^. Moreover, glycated heat-shock protein 27 was also detected, which we previously determined to have an important protective role against aSyn pathology^139^, particularly in glycation conditions^57^.

## Conclusions

The pathogenesis of PD and the mechanisms leading to the accumulation and aggregation of aSyn remain poorly understood. Type-2 diabetes mellitus is an important risk factor for PD and, interestingly, glycation mediates aSyn pathogenesis in vitro and in animal models. In this study, we demonstrate that glycation exacerbates PD-like motor and non-motor features in Thy1-aSyn mice. These alterations are followed by the accumulation of aSyn in the midbrain, striatum, and prefrontal cortex, by increased aSyn phosphorylation (pS129) and insolubility, followed by general neuronal loss in the vicinity of the SNpc. Furthermore, we found that MGO-injected Thy1-aSyn mice show pronounced alterations in proteins of the glutamatergic pathway within the midbrain, suggesting an increased production of glutamate and increased glutamatergic transmission. Moreover, several of the glycated proteins are components of PD-associated pathways. Thus, we suggest that in conditions of aSyn pathology, MGO induces glutamatergic hyperactivity in the midbrain aggravating both motor and non-motor features in mice (Fig. 10a)^40,121^. Moreover, we suggest that MGO and aSyn overexpression commonly dysregulate several mechanisms that potentially trigger pathways of neurodegeneration (Fig. 10b, c).

In conclusion, we uncovered a major role for MGO-derived glycation in the exacerbation or anticipation of PD-like features, suggesting that anti-diabetic/anti-glycation agents hold promise as disease-modifying agents in PD. Likewise, glutamatergic-silencing molecules may suppress excitotoxic events that underlie neuronal degeneration in synucleinopathies.

## Methods

### Methylglyoxal production and standardization

MGO was synthesized by sulfuric acid hydrolysis of 1,1-dimethyl acetal and purified by fractional distillation^56,137^. Purified MGO was diluted to estimated 50–100 μM in a solution with 1 mM aminoguanidine hydrochloride in 50 mM sodium phosphate buffer (pH 7.4). The mixture was incubated at 37 °C for 4 h. The reaction between MGO and aminoguanidine forms aminotriazine, whose absorbance was measured spectrophotometrically at 320 nm, from which the concentration of MGO is deduced, according to ε_320_ = 2411 M^−1^cm^−^^1^ ^140,141^.

### Animals

Animal procedures were carried out in accordance with the European Community guidelines (Directive 2010/63/EU), Portuguese law on animal care (DL 113/2013), and approved by the iMM Internal Committee and the Portuguese Animal Ethics Committee (Direcção Geral de Alimentação e Veterinária - DGAV).

Animals were maintained under controlled light (12 h light/12 h dark cycle) and environmental conditions, with a constant temperature of 21 ± 0.5 °C, and relative humidity of 60 ± 10%, and had free access to commercial chow and water (ad libitum). Mice were housed in groups, with two to five animals per cage. Only male animals were used in all experimental procedures. Mice were sacrificed by exsanguination, through the perfusion of PBS from the heart, after anesthesia under isoflurane atmosphere.

We started by evaluating if glycation contributes to the onset or if it exacerbates PD-like features. For that purpose, we used Thy1-aSyn mice as a model of synucleinopathies. This model recapitulates several features of PD, including aSyn pathology, alterations in nigrostriatal dopaminergic pathway, loss of striatal dopamine and TH, and a progressive neurodegenerative process, with gradual motor and non-motor deficits^58,59^.

Transgenic mice overexpressing human aSyn under the Thy1 promoter were generated on a mixed C57BL/6-DBA/2 background^59^. Animals were obtained from our breeding colony on this background by breeding mutant females with WT C57BL/6-DBA/2 males. Offspring were genotyped via polymerase chain reaction (PCR) amplification analysis of DNA extracted from ear or toe. PCR was performed using the following primers: Thy1- F: 5′-CTG GAA GAT ATG CCT GTG GA-3′, Thy1-R: 5′-GAG GAA GGA CCT CGA GGA AT-3′, with an annealing temperature of 60 °C and 40 cycles of amplification as previously^91^.

### Ethics approval and consent to participate

All procedures were performed according the FELASA guidelines and recommendations concerning laboratory animal welfare. All procedures were approved by the national directorate-general of food and veterinary (DGAV) and by Instituto de Medicina Molecular Ethics Committee.

### Mouse demographics and group characterization

The cohort consisted of 27 male Thy1-aSyn and WT littermate mice, distributed among the four experimental groups: vehicle- (1); or MGO-injected (2) WT littermate mice; and vehicle- (3); or MGO-injected (4) Thy1-aSyn mice (Supplementary Fig. 1a, b). Vehicle-injected or MGO-injected WT littermate mice group included 5 animals each, with an average age of 16.00 ± 1.00 weeks and weight of 29.62 ± 4.12 or 29.01 ± 3.62 g, respectively (Supplementary Fig. 1a, b). Vehicle-injected or MGO-injected Thy1-aSyn mice group was composed of nine or eight animals, respectively, with an average age of 16.11 ± 0.78 or 16.25 ± 0.89 weeks and weight of 28.66 ± 1.43 or 28.19 ± 2.47 g (Supplementary Fig. 1a, b). Since Thy1-aSyn female mice exhibit mild behavioral changes, we decided to only include male mice in our study^58,142^.

### Intracerebroventricular injection of MGO and general procedures

Age-matched 16-week-old, male transgenic Thy1-aSyn and WT littermates mice received MGO, or vehicle (PBS, pH 7.4) ICV injection under deep anesthesia (80 mg/kg ketamine hydrochloride, 5 mg/kg xylazine hydrochloride). Animals were kept anesthetized using isoflurane (2–4%) and kept at a constant body temperature using a conventional heat pad. Briefly, 5 µL of MGO (31.6 mM) or PBS were injected into the right lateral ventricle, with the following stereotaxic coordinates, relative to Bregma: anterior-posterior: 0.5 mm, medial-lateral: –1.0 mm, and dorsal-ventral: –2.0 mm. The bolus injection was performed using a Hamilton syringe, attached to a micropump system with a flow rate of 0.5 μL/min. Following the surgery, animals were allowed to recover from the procedure.

Three weeks post injection, mice were weighed, handled, and general phenotype assessed by SHIRPA analysis. Behavioral testing was performed starting four weeks after surgery. Upon conclusion of the behavioral phenotyping, mice were sacrificed (Supplementary Fig. 1c). Brains were collected and separated into left and right hemispheres: the right hemisphere was transferred into paraformaldehyde (PFA) solution for fixation and immunohistological analysis; the left hemisphere was dissected and rapidly frozen in liquid nitrogen for biochemical and SWATH-MS analysis. Baseline body weight prior to the surgery was measured. Body weight was recorded immediately before mice sacrifice.

### Behavioral tests

Mice were handled once a day for 5 days prior to behavior evaluation. Mazes were cleaned with a 10% ethanol solution between each animal. All behavioral tests were done during the light phase between 8 a.m. and 9 p.m. in a sound attenuated room. Prior to each test, animals were allowed to acclimate to the room for 30 min.

To evaluate the effects of glycation, mice underwent a battery of behavioral tests to characterize motor, cognitive, anxiety-related, and olfactory function. Open field test, pole test, rotarod, wire hang test, and adhesive removal test were performed to evaluate motor function. Y maze test was used to assess spatial short-term memory (hippocampal dependent), as a read-out of cognitive function. Anxiety-related behavior was evaluated with elevated plus maze test. The block test was performed to assess olfactory function. SHIRPA protocol was also used to evaluate general health behavior.

#### SHIRPA protocol

SHIRPA protocol was used to evaluate general health behavior and phenotype characterization. We evaluated hindlimbs clasping and colonic function. Hindlimbs clasping is a marker of disease progression and cerebello-cortico-reticular and cortico-striato-pallido-reticular pathways function assessment^61^. In this test, the mouse was suspended by the tail and the extent of hindlimb clasping observed for 30 s and scored from 0 to 3: score 0—both hindlimbs were splayed outward away from the abdomen with splayed toes; score 1—one hindlimb was retracted or both hindlimbs were partially retracted; score 2—both hindlimbs were partially retracted toward the abdomen and were touching the abdomen; score 3—both hindlimbs were fully clasped and touching the abdomen.

Colonic function was assessed during open field test^61,66^. The number of fecal pellets were counted after 10 min in the open field arena.

#### Open field test

The open field test was used to observe general motor activity, gross locomotor activity, and exploration habits^60^. Assessment took place in a square arena (40 cm length × 40 cm wide × 40 cm height) with opaque walls, and a single trial was done. The mouse was placed in the center of the arena and allowed to freely explore for 10 min, while being recorded by an overhead camera. The footage was then analyzed by an automated tracking system and distance moved, velocity, and time spent in pre-defined zones were measured. By observation, we recorded rearing (standing up on hind limbs) and grooming behaviors, and defecation and urination.

#### Pole test

The pole test was performed to assess locomotor activity, mainly motor coordination, and balance^60–63^. The pole was composed of metal rod with 50 cm length and a diameter of 1 mm wrapped with paper tape. The base of the pole was placed in a cage filled with bedding material. The mouse was placed head-upward close to the top of a vertical pole and was expected to orient downward and descend the length of the pole back into the cage. A maximum time of 180 s was given to complete the task. Each mouse underwent one training and four trials, with 30-min intervals. Training and trial tests were recorded. The time to turn down, to climb down and total time were measured manually. Data from four trials were averaged for each mouse and presented as average plus standard deviation.

#### Rotarod test

The rotarod test was used to evaluate motor coordination, balance, and motor learning^60–63^. A commercial apparatus with a rat rod with a diameter of 6 cm was used. Animals underwent one training and three trials at 30-min intervals. During the training, the mouse was placed on the rotating rod at 7 rpm until it could stand on the rod (about 2–3 min). For testing, the mouse was placed on a rotating rod with either constant rotation (7 rpm) or continuous acceleration (from 4 to 40 rpm in 5 min). In the steady rotation protocol, the animals were placed on the rotating rod at a constant speed of 7 rpm, for three minutes. In accelerating conditions, the mouse was placed on the rotating rod at a constant speed of 4 rpm that gradually accelerates from 4–40 rpm in 10 min. The latency to fall was recorded for both protocols. Data are presented as average of the three trials for each mouse.

#### Wire hang test

Wire hang test was used to evaluate balance and grip strength^60,64^. The mouse was placed hanging from an elevated wire cage top, which was then inverted and suspended above the home cage (1 m) for 60 s. Animals underwent three trials at 30-min intervals. The latency to fall was recorded. Data are presented as average of the three trials for each mouse.

#### Adhesive removal test

Adhesive removal test was performed to evaluate sensory and motor deficits related to the paw and the mouth^65^. This test consists of applying a white adhesive tape of 0.8 mm of diameter onto the snout of the mouse. The animal is then released and the time-to-remove the adhesive was measured. Each mouse underwent one training and three trials with 15-min intervals. Data are presented as average of the three trials for each mouse.

#### Y maze test

Y maze test was used to assess short-term spatial reference memory, which is hippocampal dependent^67,68^. The test was performed in a Y-shaped maze with three arms (arm A: 20 cm length × 5 cm wide × 12 cm height, arms B and C: 15 cm length × 5 cm wide × 12 cm height), angled at 120° and with opaque walls. During the training, the mouse was placed in the start point of the Y maze with a closed arm and allowed to freely explore it for 6 min. After one hour, the “novel” arm was open, and the mouse allowed to freely explore the maze for 5 min. A single trial was done and recorded by an overhead camera. Time spent in the novel arm was measured manually, as well as the number of triads (i.e., ABC, CAB, or BCA but not ABB) and entries. The alternative behavior score (%) for each mouse was calculated as the ratio of the number of alternations to the possible number (total number of arm entries minus two) multiplied by 100. The maze was cleaned with diluted 10% ethanol between tests to eliminate odors and residues. Data presented result from a single trial for each mouse. One animal from vehicle-injected WT littermate group was considered an outlier since it deviated more than 1 standard deviation from the group mean of time spent on novel arm. One mouse from MGO-injected Thy1-aSyn group failed to complete the test since it was too stressed.

#### Elevated plus maze test

To characterize anxiety-related behavior, the elevated plus maze test was used^69,70^. The apparatus has a small central platform with four arms radiating outwards and is raised above the ground to a height of 75 cm. The arms are placed at an angle of 90° from each other and are 50 cm in length and 10 in width. Alternating arms are enclosed by high opaque walls of 50 cm height, with open tops. The mouse was placed in the center of the maze and allowed to freely explore for 5 min for a single trial and recorded by an overhead camera. The time spent on open arms and the number of entries were measured manually. Data presented result from a single trial for each mouse.

#### Block test

We performed the block test to assess olfactory function^71,72^. This test evaluates sensitivity to social smells, an ethologically essential ability in mice, thus measuring the olfactory acuity and discrimination. Housed animals were exposed to five wood blocks (2 cm) placed inside each cage for 7 days. During this period, the cage bedding was not replaced. Upon testing, four blocks originally from the mouse’s own cage were placed into a new cage, approximately 1–2 cm apart. The mouse was placed on the novel cage and videotaped for 30 s. Each mouse underwent four training sessions. On the trial test, one block was replaced by a block that was originally in a cage with a different set of animals. The mouse was videotaped for 1 min. The time sniffing novel scent was measured manually. Data presented result from a single test trial for each mouse. Two mice from vehicle-injected Thy1-aSyn group displayed freezing behavior and failed to perform the test.

### Immunohistochemistry and counting of neuronal populations of mice brain

After mice sacrifice, the right hemisphere of the brain was transferred into PFA solution for fixation. Next, they were cryoprotected in TBS (pH 7.6) containing 30% sucrose (w/v) overnight at 4 °C. Sagittal free-floating sections (30 mm) were cut around the region of *substantia nigra* using a cryostat (Leica CM 3050S, Germany), and subsequently stained^143^. Free-floating sections were blocked (5% Goat Serum (BioWest, France) and 1% Bovine serum (VWR, USA) and then incubated with different primary antibodies, specifically anti-tyrosine hydroxylase (TH) rabbit (Millipore, 1:1000), anti-aSyn (1:1000, BD Transduction laboratories), anti-NeuN mouse (Millipore, 1:400) overnight at 4 °C. Sections were then washed with TBS and incubated with Alexa Flour 488/555 secondary antibodies (1:1000, Invitrogen). Sections were mounted in SuperFrost^®^ Microscope Slides using Mowiol mounting media (Calbiochem). Omission of the primary antibody resulted in no staining. Image acquisition (10× for a whole brain; 63× and 100× in the *substantia nigra* region) was performed under a confocal point-scanning microscope using both z-stack and scan tile (Zeiss LSM 800 with Airyscan). Confocal imaging was undertaken at European Neuroscience Institute.

Numbers of TH-, DAPI- or NeuN-positive neurons were counted manually, blinded for experimental grouping, using Cell Counter plugin to mark cells from Fiji open source software^144^. For NeuN-positive cell counting, we first performed a maximum projection for z-stack of the 63× images of the SNpc region. Next, a threshold of the image was performed to detect the NeuN-positive staining in the image. Lastly, “analyse particles” plugin from Fiji open source was activated to count the number of NeuN-positive cells in the image. For each animal within the groups, at least four sagittal slices were quantified, and an average used for statistical analysis.

### Tissue lysate preparation

In all, 200 µL of RIPA buffer (50 mM Tris-HCl pH 7.4, 150 mM NaCl, 2 mM EDTA, 0.1% SDS, 0.25% sodium deoxycholate) were added per 0.02 g of brain tissue. Samples were macerated with an automatic pestle. Samples underwent three cycles of sonication in pulses (1 s on, 45 milliseconds off, for 30 s, with 15% of intensity), with 1 min incubation on ice between them. Protein extracts were centrifuged for 10 min at 9.600 rcf at 4 °C to pellet tissue and cell debris. Supernatant was collected and total protein was quantified using Pierce^®^ BCA Protein Assay Kit (Thermo Fisher Scientific; Waltham, MA, USA)^56^.

### aSyn solubility analysis

aSyn solubility in Triton X-100 (1%) was evaluated using 200 μg of total protein extract^56,145^. Triton X 100 was added to protein extracts at a final concentration of 1%, followed by an incubation at 4 °C for 30 min. The soluble and insoluble fractions of the protein extract were separated by centrifugation at 16,000 g, at 4 °C for 1 h. The supernatant, containing the soluble fraction, was collected and the pellet, containing the insoluble fraction, was resuspended in 40 µL of PBS supplemented with sodium dodecyl sulfate (SDS, 2% final concentration) and cOmplete™, Mini, EDTA-free Protease Inhibitor (Roche; Basel, Switzerland). Soluble fraction (10 µL), and insoluble fraction (15 µL), both supplemented with 2% SDS, were loaded and resolved by SDS-Page and immunoblotted. aSyn insolubility is given by the ratio between the amount of aSyn in the insoluble fraction and the sum of aSyn in both soluble and insoluble fractions.

### Immunoblot analysis

#### Gel separation or dot-blots

A total of 10 or 15 μg of total protein from tissue lysates was separated by SDS-PAGE electrophoresis using a Tetra cell (Bio-Rad; Hercules, CA, USA), in 12% polyacrylamide separation gel and a 4% polyacrylamide stacking gel, applying a constant voltage of 120 V. Pre-stained standard proteins and pool sample, composed by 10 µL of cerebellum samples from each mouse, were also loaded onto the gel for inter-gel normalization purposes. Ten micrograms of total protein from tissue lysates were also loaded onto nitrocellulose membranes using a dot-blot system, and the wells washed twice with PBS before removing the membrane from the apparatus.

#### Western-blotting procedures

Gel separated proteins were transferred to nitrocellulose membranes, using standard procedures with a Mini Trans-Blot system (Bio-Rad; Hercules, CA, USA). Membranes were incubated with blocking solution (5% bovine serum albumin) in 1× TBS (20 mM Tris, 136 mM NaCl, pH 7.6) at room temperature for 30 min. Primary antibody incubations were carried out overnight at 4 °C, using given concentrations in blocking solution: CEL (Mouse Anti-N^ε^-carboxyethyl lysine, in a dilution of 1:1000 in blocking solution, Cosmo-Bio, USA), aSyn (Purified Mouse Anti-α-Synuclein antibody, in a dilution of 1:1000 in blocking solution, BD Biosciences; San Jose, CA, USA), phosphorylated aSyn at residue 129 (pS129, [J18] SMC-600, StressMarq; Victoria, Canada), glyoxalase I (Rabbit polyclonal anti-Glyoxalase I antibody – FL-184, sc67351, Santa Cruz Biotechnology), and β-actin (Mouse Monoclonal anti-β-actin antibody in a dilution of 1:5000 in blocking solution, Ambion, Thermo Fisher Scientific; Waltham, MA, USA). Membranes were washed and incubated with the appropriate secondary antibody (ECL™ Anti-Mouse IgG, HRP-Linked antibody in a dilution of 1:5000 in blocking solution, Amersham™; Little Chalfont, UK; Anti-Rabbit IgG, HRP-Linked antibody in a dilution of 1:5000 in blocking solution, Amersham™; Little Chalfont, UK) for 1.5 h. Detection procedures were carried on according to ECL system (GE Healthcare, Life Sciences; Little Chalfont, UK), and the signal detected using a ChemiDoc™ Imaging Systems (Bio-Rad, Hercules, CA, USA) with the most appropriate exposure time. Densitometry was performed using ImageJ—Image Processing and Analysis in Java^146^. When required, membranes were incubated with stripping solution (250 mM Glycine, 0.1% of 10% SDS, pH 2.0) for 45 min at room temperature with agitation, followed by four washing steps, twice with 1× TBS and twice with 1× TBS supplemented with 10% Tween 20 solution. Membranes were then incubated in blocking solution for 30 min before reprobing with the required antibodies.

### SWATH-MS analysis

A high-throughput proteomics analysis using NanoLC coupled to the TripleTOF 6600 (at UniMS, Mass Spectrometry Unit at iBET/ITQB) was performed to screen for differences in protein expression between experimental groups of midbrain or prefrontal cortex protein extracts.

#### Sample preparation for mass spectrometry analyses

Eighty micrograms of each sample were reduced with 10 mM dithiothreitol (BioUltra, Sigma) for 45 min at 56 °C, alkylated with 20 mM iodoacetamide (BioUltra, Sigma) for 30 min at room temperature in the dark, and then precipitated with acetone (HPLC Plus, Sigma) overnight at −20 °C. The dried sample was resuspended in 50 mM ammonium bicarbonate (BioUltra, Sigma) and digested overnight at 37 °C with trypsin (Sequencing Grade Modified Trypsin, Promega) in a 1:50 trypsin:sample ratio. A second digestion was performed for 3 h at 37 °C, by adding trypsin in a 1:100 trypsin:sample ratio with 80% (v/v) acetonitrile (Optima LC/MS grade, Fisher Scientific). The sample was dried on a SpeedVac (ThermoSavant Scientific) and resuspended in 5% Formic acid (Optima LC/MS grade, Fisher Scientific) to perform peptide cleanup using C18 microcolumns (OMIX C18 pipette tips, Agilent), and then dried again.

#### Information-dependent acquisition (IDA) runs to generate the spectral library

Nano-liquid chromatography-tandem mass spectrometry analysis was performed on an ekspert™ NanoLC 425 cHiPLC system coupled with a TripleTOF 6600 with a NanoSpray III source (Sciex Framingham, US). Peptides were sprayed into the MS through an uncoated fused-silica PicoTip™ emitter (360 µm O.D., 20 µm I.D., 10 ± 1.0 µm tip I.D., New Objective, Oullins, France). The source parameters were set as follows: 15 GS1, 0 GS2, 30 CUR, 2.5 keV ISVF, and 100 °C IHT. Peptides were separated through reversed-phase chromatography in a trap-and-elute mode. Trapping was performed at 2 µL/min on a NanoLC Trap column (Eksigent 350 µm × 0.5 mm, ChromXP C18-CL, 3 µm, 120 Å) with 100% A (0.1% formic acid in water, Fisher Chemicals, Geel, Belgium) for 10 min. The separation was performed at 300 nL/min, on a NanoLC column (Eksigent 75 µm × 15 cm, ChromXP 3C18-CL-120, 3 µm, 120 Å). The gradient was as follows: 0–1 min, 5% B (0.1% formic acid in acetonitrile, Fisher Chemicals, Geel, Belgium); 1–91 min, 5–30% B; 91–93 min, 30–80% B; 93–108 min, 80% B; 108–110 min, 80–5% B; 110–127 min, 5% B.

The samples used to generate the SWATH-MS spectral library were subjected to IDA using three different MS m/z ranges, which were calculated using the SWATH Variable Window Calculator V1.0 (Sciex, Framingham, US) based on a reference sample. The mass range for MS scan was set to m/z 400–614.9, 613.9–791.7, and 790.7–2000. The MS/MS scan mass range was uniformly set to m/z 150–1800. Following a TOF-MS survey scan (250 msec accumulation time), the 50 most intense precursors were selected for subsequent fragmentation and the MS/MS were acquired in high sensitivity mode for 40 msec, for a total cycle time of 2.3 s. The selection criteria for parent ions included an intensity of greater than 125 cps and a charge state ranging from +2 to +5. Once an ion had been fragmented through MS/MS, its mass was excluded from further MS/MS fragmentation for 12 s. The ions were fragmented in the collision cell using rolling collision energy, and CES was set to 5. Biological replicates (individual animals) were pooled to perform the IDA runs. Therefore, the 24 IDA MS raw files were combined and subjected to database searches in unison using ProteinPilot software v. 5.0 (Sciex, Framingham, US) with the Paragon algorithm to generate the Spectral Library. A UniProt reviewed database (17,032 entries, accessed on October 16, 2019) containing the sequences of Mus musculus was used. The following search parameters were set: Cys alkylation: Iodoacetamide; Met oxidation; lys glycation: Argpyrimidine [Nd-(5-hydroxy-4,6-dimethylpyrimi- dine-2-yl)-l-ornithine], hydroimidazolones [Nd-(5-meth- yl-imidazolone-2-yl)-ornithine, isomers and oxidation products] and Nd-(4-carboxy-4,6-dimethyl-5,6-dihydr- oxy-1,4,5,6-tetrahydropyrimidin-2-yl)ornithine, and CEL, and general modifications. Digestion: Trypsin; Instrument: TripleTOF 6600; ID focus: Biological modifications and Amino acid substitutions; Search effort: Thorough; false discovery rate (FDR) analysis: Yes. Only the proteins with <1% FDR were considered.

#### Protein quantification by SWATH-MS

Five biological replicates from each condition were analyzed by SWATH-MS, using the instrument setup described for the IDA runs. The mass spectrometer was set to operate in cyclic data-independent acquisition, similarly to the previously established method (Gillet et al., 2012). SWATH-MS data were acquired using the SWATH acquisition method, applying a set of 64 overlapping variable SWATH windows covering the precursor mass range of 400–2000 m/z. The variable SWATH windows were calculated using the SWATH Variable Window Calculator V1.0 (Sciex, Framingham, US) based on the same reference sample. At the beginning of each cycle, a 10 ms survey scan (400–2000 m/z) was acquired, and the subsequent SWATH windows were collected from 150 to 1800 m/z for 50 ms, resulting in a cycle time of 3.26 s. The collision energy for each window was set using rolling collision energy, and CES was set to 5.

Data processing was performed using the SWATH processing plugin for PeakView 2.2 (Sciex, Framingham, MA USA). In brief, peptides were selected from the library using the following criteria: (i) the unique peptides for each protein were ranked by the precursor ion intensity from the IDA runs as estimated by the ProteinPilot software: (ii) shared peptides (peptides that shared the same amino acid sequence between different protein entries) were excluded from selection. Up to six peptides were chosen per protein, and SWATH quantification was attempted for all proteins in the library file that were identified below 1% Global FDR from fit from ProteinPilot searches (which corresponded to a peptide confidence threshold of 98%). Target fragment ions, up to six, were automatically selected. Manual inspection was performed for random peptides to check the quality of the auto selection and fragment ions were edited accordingly. Peak group confidence threshold was determined based on an FDR analysis using the target-decoy approach, and extraction FDR threshold was set to 1%. Peptides that met the 1% FDR threshold in all the five replicates were retained. The peak areas of the fragment ions were extracted using an XIC window of 6 min, and an XIC width of 20 ppm. Data were directly exported to MarkerView 1.3.1 (Sciex, Framingham, MA USA) and normalized using total area sums to obtain the final quantification values. MarkerView was also used to perform the PCA and *t*-test statistical tests.

#### Quantitative analysis

An outlier removal approach was executed using GraphPad Prism Version 9, using iterative Grubb’s using Alpha = 0.2. Missing values were replaced using average values from the corresponding group of samples. Protein intensities were then divided by the number of theoretically observable peptides between 6 and 30 amino acids, neglecting missed cleavages, to obtain normalized iBAQ values^147^. The log2(*x* + 1) transformed iBAQ values were quantile normalization using the R function “normalize.quantiles”^148^. The normalized values were subjected to statistical analysis utilizing R package limma^149^ where different contrast were specified. Correction for multiple testing was applied using the method of Benjamini and Hochberg^150^.

#### Proteome functional analysis

Venn diagrams of the statistically differently regulated proteins in midbrain or prefrontal cortex between the experimental groups were used to identify the uniquely affected hits affected in Thy1-aSyn mice injected with MGO using the tool InteractiVenn^151^. These were identified via the comparison between Thy1-aSyn mice injected with MGO and vehicle, excluding the common hits between Thy1-aSyn mice injected with vehicle and the WT mice injected with vehicle (excluding hits generally affected by aSyn expression); and between WT mice injected with MGO vs vehicle (excluding hits generally affected by MGO glycation).

The peptides with MAGEs and the corresponding proteins were identified in the midbrain of all experimental groups. Venn diagrams were used to identify proteins uniquely glycated in the Thy1-aSyn mice injected with MGO.

Representation of the number of statistically significant up- and downregulated hits was performed in GraphPad Prism V9 volcano plots. Protein–protein functional associations were retrieved from STRING (http://www.string-db.org/, version 11.0)^152^. We used the online Enrichr tool (http://www.amp.pharm.mssm.edu/Enrichr/) for the functional enrichment analysis, including KEGG and GO resources^153^. Pathways were obtained from “KEGG 2019 Mouse” and GO terms from “GO Biological Process 2018”, “GO Molecular Function 2018”, and “GO Cellular Component 2018”. Top 7 pathways or GO terms with the highest −log_10_ Fisher exact test *p* value were selected (*p* values below 0.05). Heatmaps representing the differently regulated proteins per top 3 KEGG pathways were done in GraphPad Prism version 9. Protein–protein functional associations representations were color-coded according to the top KEGG and GO analysis in STRING (Search Tool for the Retrieval of Interacting Genes/Proteins)^152,154–163^. The minimum required interaction score was defined for high confidence (0.7) and disconnected nodes in the network were hidden.

### Statistical analysis

Each experimental group was composed at least of five mice, unless stated otherwise, and all values are expressed as normalized means plus standard deviation. Statistical analysis was performed using GraphPad Prism version 9. One-way ANOVA were used to compare differences among conditions and groups, followed by Dunnett’s multiple comparison test. Unpaired two-tailed *t*-test with equal SD was performed to compare differences between two experimental groups. Values of *p* < 0.05 were considered significant.

### Reporting summary

Further information on research design is available in the Nature Research Reporting Summary linked to this article.

## Supplementary information

Supplementary Material

Reporting Summary

## Data Availability

The mass spectrometry proteomics data have been deposited to the ProteomeXchange Consortium via the PRIDE^164^ partner repository with the dataset identifier PXD032832. All other relevant data are available from the authors.
