# Supplementary material for: Glycation modulates glutamatergic signaling and exacerbates Parkinson’s disease-like phenotypes
Source: NPJ Parkinsons Dis. 2022 Apr 25;8:51. doi: 10.1038/s41531-022-00314-x (PMC9038780; doi:10.1038/s41531-022-00314-x)
Supplement: Supplementary file 1 — Supplementary Material [file 41531_2022_314_MOESM1_ESM.pdf]

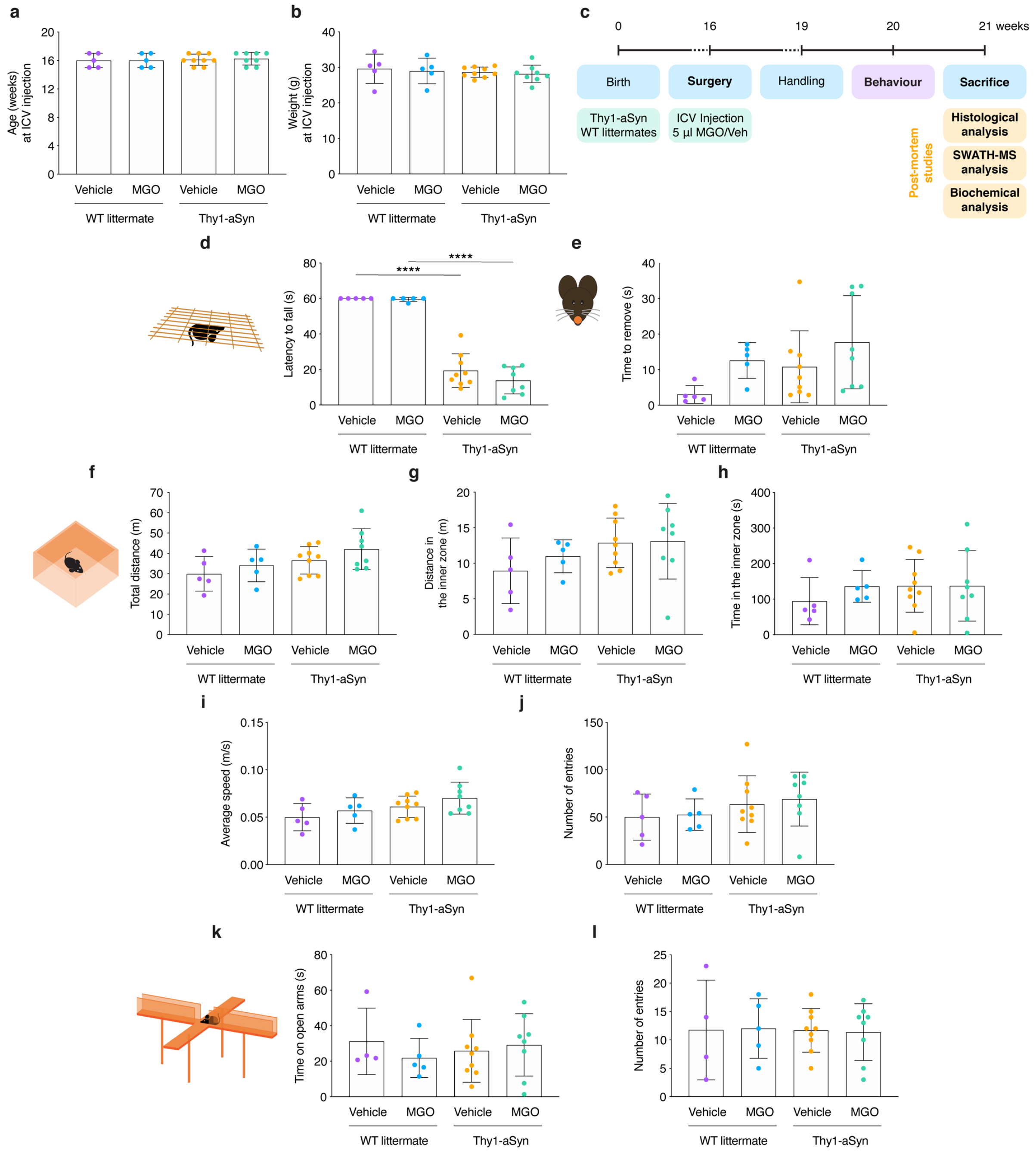

Supplementary Figure 1. Non-altered mice behavior. Plot representations of mice demographics: **(a)** age in weeks and **(b)** weight in g at MGO ICV injection. **(c)** Schematic of experimental design: wild-type littermate (WT) and Thy1-aSyn transgenic (Tg) mice received an intracerebroventricular (ICV) injection of MGO or vehicle (PBS) at 16 weeks of age. Behavioral testing started 4 weeks post-surgery. Plot representations of: (**d**) wire hang test; (**e**) adhesive removal test; open field test - (**f**) total distance, (**g**) distance in the inner zone, (**h**) time in the inner zone, (**i**) average speed, (**j**) number of entries; Elevated plus maze test - (**k**) time on open arms, and (**l**) number of entries; At least n=5 in all groups, data in all panels are average ± standard deviation.

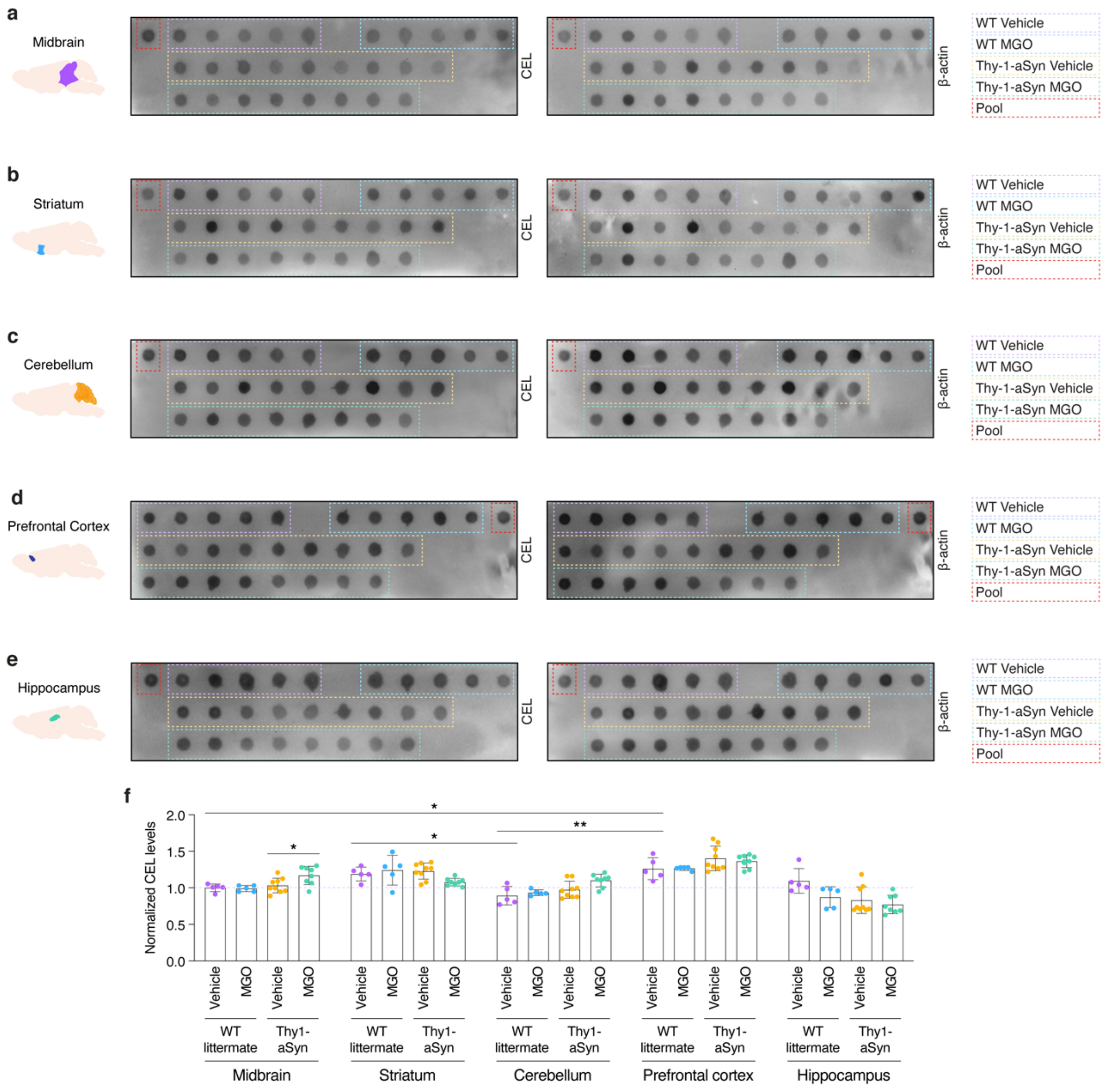

Supplementary Figure 2. Signal of glycated protein in different brain regions of all experimental groups. Wild-type littermate (WT) and Thy1-aSyn transgenic mice received an intracerebroventricular (ICV) injection of MGO or vehicle (PBS) and protein brain extracts from several regions analyzed 5 weeks post-injection. Protein extracts loaded into nitrocellulose membranes in a dot blot system and probed with anti-CEL and anti-β-actin, for normalization. Representative blots from all samples from each experimental group and a pool sample for inter-gel comparison are shown for **(a)** midbrain, **(b)** striatum, **(c)** cerebellum, **(d)** prefrontal cortex and **(e)** hippocampus, respectively. (**f**) Plots of the densitometric analysis is shown comparing CEL levels between all areas and experimental groups. At least n=5 in all groups, data in all panels are average ± standard deviation, Ordinary one-way ANOVA, $*p < 0.05$, $**p < 0.01$.

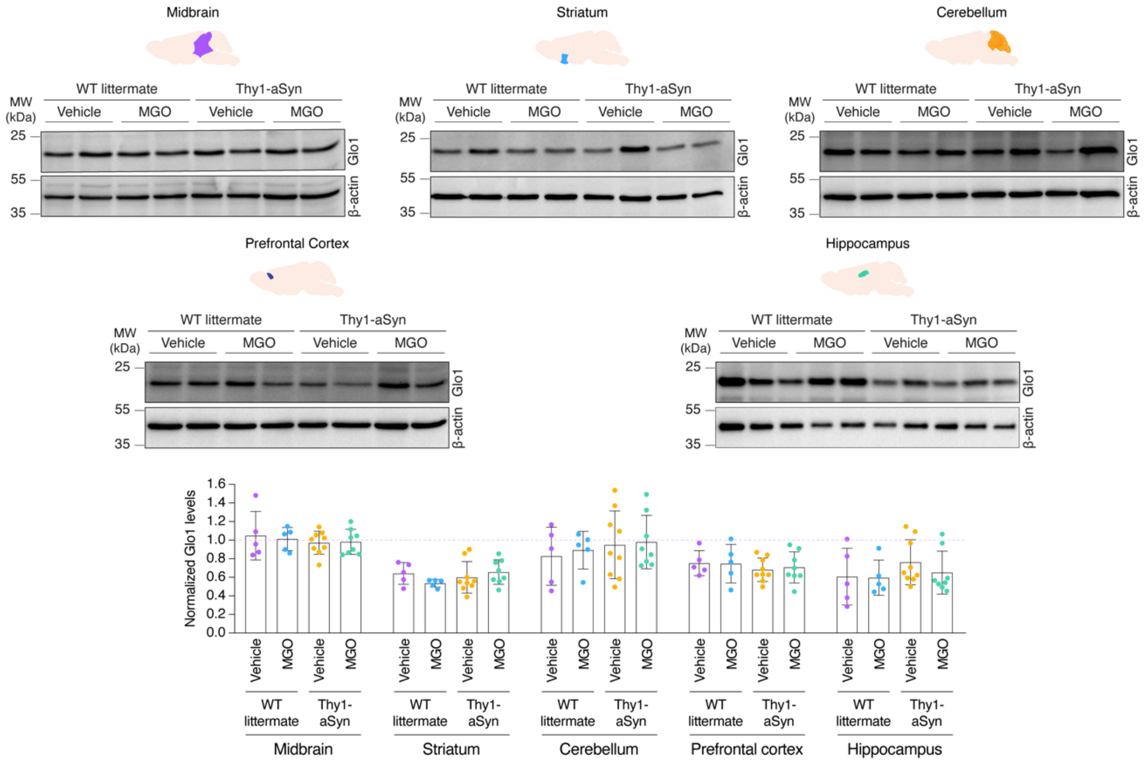

Supplementary Figure 3. The levels of glyoxalase I are similar across different brain regions of all experimental groups. Wild-type littermate (WT) and Thy1-aSyn transgenic mice received an intracerebroventricular (ICV) injection of MGO or vehicle (PBS) and protein brain extracts from several regions analyzed 5 weeks post-injection. Protein extracts were resolved by SDS-PAGE, loaded into membranes, and probed with anti-glyoxalase I and anti-β-actin, for normalization. Representative blots including 2 samples from each experimental group are shown for glyoxalase I and β-actin. Densitometric analysis is represented comparing normalized glyoxalase I levels between all areas and experimental groups. At least n=5 in all groups, data in all panels are average ± standard deviation.

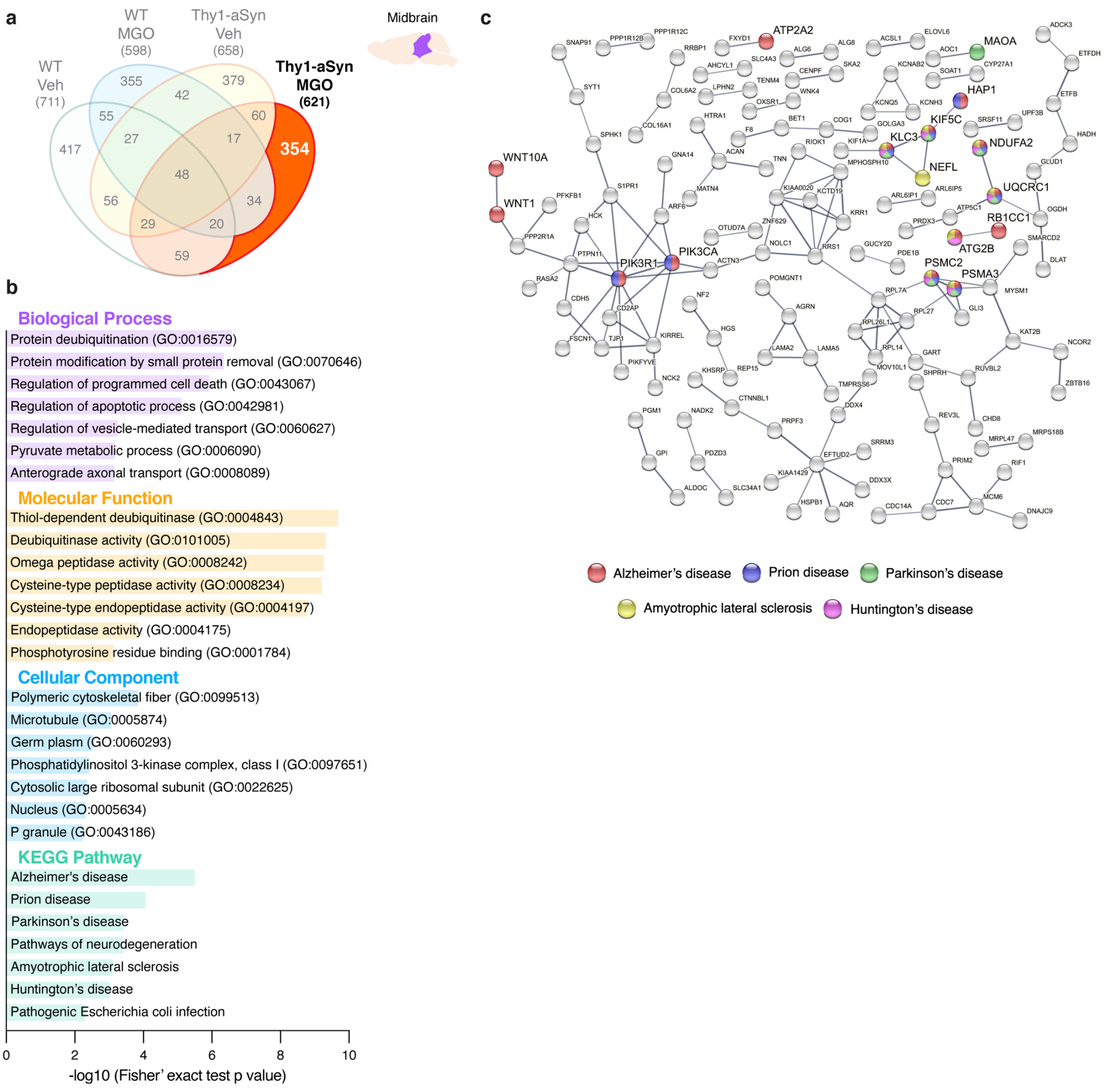

Supplementary Figure 4. Uniquely glycated midbrain proteins in MGO-injected Thy1-aSyn mice correlate with neurodegenerative-associated pathways. Glycation profile of midbrain proteins from the different experimental groups. **(a)** Venn diagram depicting uniquely glycated proteins in MGO-injected Thy1-aSyn mice (354). **(b)** KEEG pathways and gene ontology GO terms analysis is presented for the uniquely glycated proteins in MGO-injected Thy1-aSyn mice. Distribution of -log10 (Fisher exact test p value) is shown. **(c)** Protein-protein interaction networks of the uniquely glycated proteins in MGO-injected Thy1-aSyn mice, extracted from the STRING 11.0 database. Only the proteins that are interacting within a network are show. KEGG pathways or GO Biological processes are color coded.

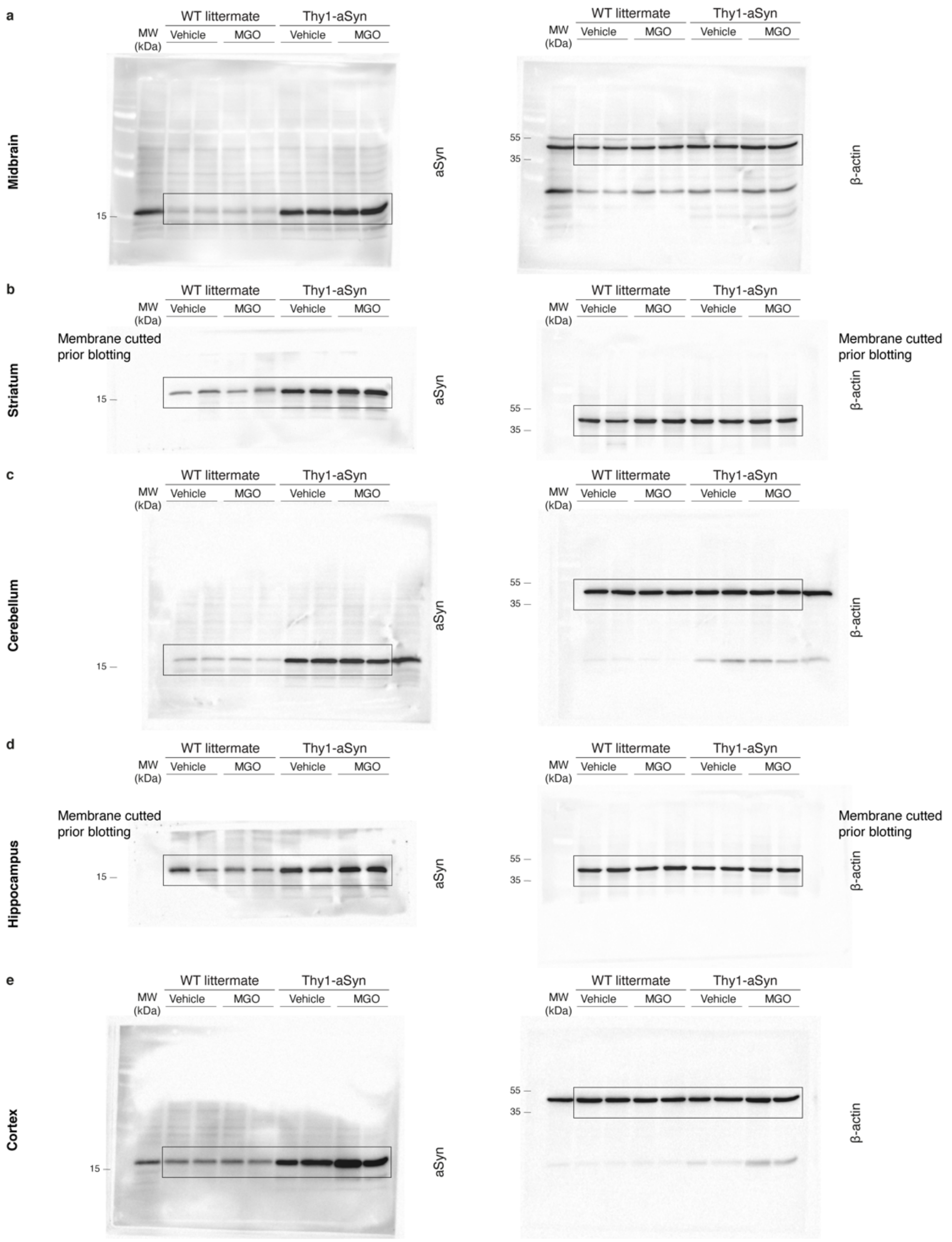

Supplementary Figure 5. Non-cropped blots of biochemical analysis. Non-cropped blots of data presented in Fig. 2.

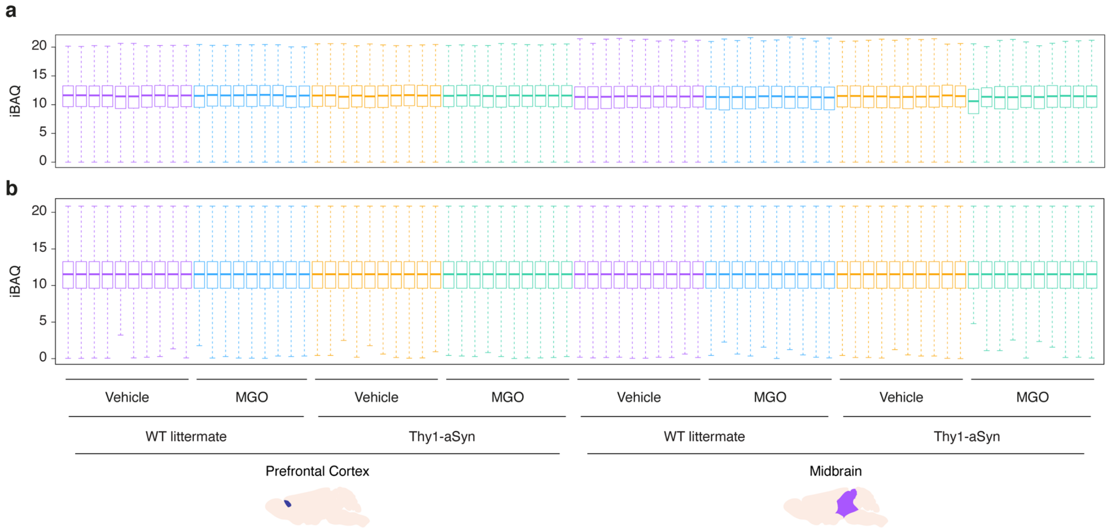

Supplementary Figure 6. Boxplots of intensity-based absolute quantification (iBAQ) values of the SWATH-MS proteomic analysis. Boxplot representation of outlier-removed **(a)** raw and **(b)** normalized iBAQ values for each performed SWATH-MS run.

Supplementary Table 1. List of uniquely altered proteins in the midbrain of MGO injected Thy1-aSyn. Mouse proteins nomenclature is used.

Protein	Annotation	Fold-change
**Mapk3**	Mitogen-activated protein kinase 3	0.17
**Svip**	Small VCP/p97-interacting protein	0.18
**Tmem33**	Transmembrane protein 33	0.23
**Map7d2**	MAP7 domain containing 2	0.25
**Cox7a2**	Cytochrome c oxidase subunit 7A2, mitochondrial	0.28
**Denr**	Density-regulated protein	0.30
**Brsk2**	Serine/threonine-protein kinase BRSK2	0.32
**Hmgb1**	High mobility group protein B1	0.36
**Rpl36**	60S ribosomal protein L36	0.37
**Dhrs1**	Dehydrogenase/reductase (SDR family) member 1	0.41
**Syp**	Synaptophysin	0.42
**Lrat**	Lecithin retinol acyltransferase	0.44
**Mecp2**	Methyl-CpG-binding protein 2	0.49
**Cmbl**	Carboxymethylenebutenolidase homolog	0.52
**Mpp1**	55 kDa erythrocyte membrane protein	0.55
**Hpcal1**	Hippocalcin-like protein 1	0.57
**Atp1b2**	Sodium/potassium-transporting ATPase subunit beta-2	0.57
**Nif3l1**	NIF3-like protein 1	0.58
**Prkar2a**	cAMP-dependent protein kinase type II-alpha regulatory subunit	0.60
**Pgd**	6-phosphogluconate dehydrogenase, decarboxylating	0.60
**Apmap**	Adipocyte plasma membrane-associated protein	0.61
**Map7d1**	MAP7 domain containing 1	0.63
**Csrp1**	Cysteine and glycine-rich protein 1	0.64
**Dctn4**	Dynactin subunit 4	0.65
**Txnl1**	Thioredoxin-like protein 1	0.65
**Anxa5**	Annexin A5	0.65
**Lgi3**	Leucine-rich repeat LGI family member 3	0.71
**Cmpk2**	UMP-CMP kinase 2, mitochondrial	0.71
**Lin7c**	Protein lin-7 homolog C	0.73
**Akr1a1**	Alcohol dehydrogenase [NADP(+)]	0.75
**Nefh**	Neurofilament heavy polypeptide	0.75
**Ethe1**	Persulfide dioxygenase ETHE1, mitochondrial	0.76
**Eci2**	Enoyl-CoA delta isomerase 2, mitochondrial	0.78
**Psat1**	Phosphoserine aminotransferase	0.80
**Serpinb1a**	Leukocyte elastase inhibitor A	0.81
**Cbr1**	Carbonyl reductase [NADPH] 1	1.19
**Npepps**	Puromycin-sensitive aminopeptidase	1.20
**Sirt2**	NAD-dependent protein deacetylase sirtuin-2	1.21
**Pfkm**	ATP-dependent 6-phosphofructokinase, muscle type	1.21

Protein	Annotation	Fold-change
**Fam162a**	Protein FAM162A	1.23
**Uba1**	Ubiquitin-like modifier-activating enzyme 1	1.24
**Fnbp1**	Formin-binding protein 1	1.24
**Hspa5**	78 kDa glucose-regulated protein	1.24
**Hspd1**	60 kDa heat shock protein, mitochondrial	1.24
**Ppp1r7**	Protein phosphatase 1 regulatory subunit 7	1.25
**Ppia**	Peptidyl-prolyl cis-trans isomerase A	1.25
**Rtcb**	tRNA-splicing ligase RtcB homolog	1.25
**Cttn**	Src substrate cortactin	1.25
**Oxct1**	Succinyl-CoA:3-ketoacid coenzyme A transferase 1, mitochondrial	1.25
**Myl12b**	Myosin regulatory light chain 12B	1.26
**Capzb**	F-actin-capping protein subunit beta	1.27
**Actr3**	Actin-related protein 3	1.27
**Atp6v1h**	V-type proton ATPase subunit H	1.27
**Ckb**	Creatine kinase B-type	1.27
**Ahcy**	S-adenosylhomocysteine hydrolase	1.28
**Opa3**	Optic atrophy 3 protein homolog	1.28
**Ncl**	Nucleolin	1.28
**Ncam1**	Neural cell adhesion molecule 1	1.28
**Letm1**	Mitochondrial proton/calcium exchanger protein	1.28
**Blmh**	Bleomycin hydrolase	1.29
**Nsfl1c**	NSFL1 cofactor p47	1.29
**Pdha1**	Pyruvate dehydrogenase E1 component subunit alpha, somatic form, mitochondrial	1.29
**Srsf1**	Serine/arginine-rich splicing factor 1	1.30
**Prkcb**	Protein kinase C beta type	1.31
**Srm**	Spermidine synthase	1.32
**Ndufa8**	NADH dehydrogenase [ubiquinone] 1 alpha subcomplex subunit 8	1.33
**Dusp3**	Dual specificity protein phosphatase 3	1.33
**Inpp1**	Inositol polyphosphate-1-phosphatase	1.34
**Camkv**	CaM kinase-like vesicle-associated protein	1.34
**Slc1a3**	Excitatory amino acid transporter 1	1.34
**Hnrnpm**	Heterogeneous nuclear ribonucleoprotein M	1.35
**Dlg1**	Disks large homolog 1	1.35
**Bsn**	Protein bassoon	1.35
**Rtn1**	Reticulon-1	1.35
**Tpt1**	Translationally-controlled tumor protein	1.36
**Hdhd2**	Haloacid dehalogenase-like hydrolase domain containing 2	1.37
**Camk1d**	Calcium/calmodulin-dependent protein kinase type 1D	1.38
**Gls**	Glutaminase kidney isoform, mitochondrial	1.38
**Srcin1**	SRC kinase signaling inhibitor 1	1.39
**Gsk3b**	Glycogen synthase kinase-3 beta	1.40

Protein	Annotation	Fold-change
**Eno1**	Alpha-enolase	1.40
**Pfkl**	ATP-dependent 6-phosphofructokinase, liver type	1.41
**Rph3a**	Rabphilin-3A	1.41
**Me3**	Malic enzyme 3, NADP(+)-dependent, mitochondrial	1.41
**Gpr158**	Probable G-protein coupled receptor 158	1.41
**Ctnnd1**	Catenin (cadherin associated protein), delta 1	1.41
**Acly**	ATP-citrate synthase	1.42
**Cyfip2**	Cytoplasmic FMR1-interacting protein 2	1.42
**Ndufc2**	NADH dehydrogenase [ubiquinone] 1 subunit C2	1.42
**Snd1**	Staphylococcal nuclease domain-containing protein 1	1.43
**Fn3krp**	Ketosamine-3-kinase	1.43
**Fech**	Ferrochelatase, mitochondrial	1.43
**Ppp1r1b**	Protein phosphatase 1 regulatory subunit 1B	1.43
**Ube2k**	Ubiquitin-conjugating enzyme E2 K	1.45
**Sec31a**	Protein transport protein Sec31A	1.46
**Sh3bgrl3**	SH3 domain-binding glutamic acid-rich-like protein 3	1.47
**Actr2**	Actin-related protein 2	1.47
**C1qbp**	Complement component 1 Q subcomponent-binding protein, mitochondrial	1.48
**Gpd1l**	Glycerol-3-phosphate dehydrogenase 1-like protein	1.48
**Dpp10**	Inactive dipeptidyl peptidase 10	1.49
**Slc2a3**	Solute carrier family 2, facilitated glucose transporter member 3	1.50
**Gria3**	Glutamate receptor 3	1.50
**Rgs7**	Regulator of G-protein signaling 7	1.50
**Hpcal4**	Hippocalcin-like protein 4	1.56
**Tln2**	Talin-2	1.56
**Vps45**	Vacuolar protein sorting-associated protein 45	1.56
**Prrt3**	Proline-rich transmembrane protein 3	1.56
**Mgll**	Monoglyceride lipase	1.57
**Psmd2**	26S proteasome non-ATPase regulatory subunit 2	1.57
**Acadl**	Long-chain specific acyl-CoA dehydrogenase, mitochondrial	1.59
**Isyna1**	Inositol-3-phosphate synthase 1	1.60
**Ppp3r1**	Calcineurin subunit B type 1	1.60
**Prpsap2**	Phosphoribosyl pyrophosphate synthase-associated protein 2	1.60
**Cdh2**	Cadherin-2	1.61
**Dstn**	Destrin	1.61
**Nap1l4**	Nucleosome assembly protein 1-like 4	1.61
**Dock3**	Dedicator of cytokinesis protein 3	1.61
**Akap5**	A-kinase anchor protein 5	1.62
**Actr3b**	Actin-related protein 3B	1.62
**Atp2b4**	Plasma membrane calcium-transporting ATPase 4	1.63
**Arf6**	ADP-ribosylation factor 6	1.64

Protein	Annotation	Fold-change
**Syt7**	Synaptotagmin-7	1.66
**Grin1**	Glutamate receptor ionotropic, NMDA 1	1.66
**Pafah1b2**	Platelet-activating factor acetylhydrolase IB subunit beta	1.66
**Kctd16**	BTB/POZ domain-containing protein KCTD16	1.68
**Mpp2**	MAGUK p55 subfamily member 2	1.69
**Gmfb**	Glia maturation factor beta	1.71
**Gria2**	Glutamate receptor 2	1.73
**Ahcyl2**	Putative adenosylhomocysteinase 3	1.75
**Acyp2**	Acylphosphatase-2	1.75
**Slc17a7**	Vesicular glutamate transporter 1	1.76
**Adss**	Adenylosuccinate synthetase isozyme 2	1.76
**Agap2**	Arf-GAP with GTPase, ANK repeat and PH domain-containing protein 2	1.76
**Slc27a1**	Long-chain fatty acid transport protein 1	1.77
**Mpp6**	Membrane protein, palmitoylated 6 (MAGUK p55 subfamily member 6)	1.79
**Srgap3**	SLIT-ROBO Rho GTPase-activating protein 3	1.80
**Pde1a**	Calcium/calmodulin-dependent 3',5'-cyclic nucleotide phosphodiesterase 1A	1.83
**Pde10a**	cAMP and cAMP-inhibited cGMP 3',5'-cyclic phosphodiesterase 10A	1.85
**Shank2**	SH3 and multiple ankyrin repeat domains protein 2	1.86
**Nptxr**	Neuronal pentraxin receptor	1.91
**Slc44a2**	Choline transporter-like protein 2	1.95
**Camk2b**	Calcium/calmodulin-dependent protein kinase type II subunit beta	1.98
**Jakmip3**	Janus kinase and microtubule interacting protein 3	1.98
**Phyhip**	Phytanoyl-CoA hydroxylase-interacting protein	1.99
**Ptprn2**	Receptor-type tyrosine-protein phosphatase N2	2.00
**Lphn3**	Adhesion G protein-coupled receptor L3	2.01
**Prep**	Prolyl endopeptidase	2.03
**Chgb**	Secretogranin-1	2.05
**Ncdn**	Neurochondrin	2.11
**Grin2b**	Glutamate receptor ionotropic, NMDA 2B	2.14
**Anks1b**	Ankyrin repeat and sterile alpha motif domain-containing protein 1B	2.18
**Nt5m**	5'(3')-deoxyribonucleotidase, mitochondrial	2.22
**Mpc1**	Mitochondrial pyruvate carrier 1	2.22
**Gna11**	Guanine nucleotide-binding protein subunit alpha-11	2.28
**Eif4h**	Eukaryotic translation initiation factor 4H	2.28
**Map2k4**	Dual specificity mitogen-activated protein kinase kinase 4	2.98
**Camk2a**	Calcium/calmodulin-dependent protein kinase type II subunit alpha	3.90
**Hagh**	Hydroxyacylglutathione hydrolase, mitochondrial	3.96
**Coro2b**	Coronin-2B	4.80

Supplementary Table 2. List of uniquely altered proteins in the prefrontal cortex of MGO injected Thy1-aSyn. Mouse proteins nomenclature is used.

Protein	Annotation	Fold-change
**Epn2**	Epsin-2	0.12
**Hbb-bs**	Hemoglobin, beta adult s chain	0.22
**Apoa1**	Apolipoprotein A-I	0.37
**Cul1**	Cullin-1	0.42
**Esyt1**	Extended synaptotagmin-1	0.45
**Gspt1**	Eukaryotic peptide chain release factor GTP-binding subunit ERF3A	0.48
**Krt10**	Keratin, type I cytoskeletal 10	0.50
**Atxn10**	Ataxin-10	0.52
**Alb**	Serum albumin	0.52
**Jakmip3**	Janus kinase and microtubule interacting protein 3	0.54
**Cyb5r3**	NADH-cytochrome b5 reductase 3	0.56
**Gps1**	COP9 signalosome complex subunit 1	0.58
**Eif2s1**	Eukaryotic translation initiation factor 2 subunit 1	0.62
**Gng7**	Guanine nucleotide-binding protein G(I)/G(S)/G(O) subunit gamma-7	0.63
**Rbm5**	RNA-binding protein 5	0.63
**Tmem63b**	CSC1-like protein 2	0.64
**Trmt112**	Multifunctional methyltransferase subunit TRM112-like protein	0.68
**C1qbp**	Complement component 1 Q subcomponent-binding protein, mitochondrial	0.69
**Slc6a11**	Sodium- and chloride-dependent GABA transporter 3	0.69
**Ndufb5**	NADH dehydrogenase [ubiquinone] 1 beta subcomplex subunit 5, mitochondrial	0.69
**Ssb**	Lupus La protein homolog	0.69
**Tsn**	Translin	0.70
**Aldh9a1**	4-trimethylaminobutyraldehyde dehydrogenase	0.71
**Cdh2**	Cadherin-2	0.71
**Gpx1**	Glutathione peroxidase 1	0.72
**Vsnl1**	Visinin-like protein 1	0.73
**Plp1**	Myelin proteolipid protein	0.73
**Psap**	Prosaposin	0.74
**Slc3a2**	4F2 cell-surface antigen heavy chain	0.74
**Gja1**	Gap junction alpha-1 protein	0.74
**Hp1bp3**	Heterochromatin protein 1-binding protein 3	0.75
**Ppp3r1**	Calcineurin subunit B type 1	0.75
**Rps10**	40S ribosomal protein S10	0.76
**Itgb1**	Integrin beta-1	0.76
**Ola1**	Obg-like ATPase 1	0.76
**Phgdh**	D-3-phosphoglycerate dehydrogenase	0.79
**Apoo**	Apolipoprotein O	0.79
**Atl1**	Atlastin-1	0.80
**Calb1**	Calbindin	0.80

Protein	Annotation	Fold-change
**Ndufa4**	Cytochrome c oxidase subunit NDUFA4	0.81
**Cd47**	Leukocyte surface antigen CD47	0.81
**Lancl2**	LanC-like protein 2	0.81
**Sdhb**	Succinate dehydrogenase [ubiquinone] iron-sulfur subunit, mitochondrial	0.82
**Rtn1**	Reticulon-1	0.83
**Sh3bgrl**	SH3-binding domain glutamic acid-rich protein like	0.83
**Lmna**	Prelamin-A/C	0.83
**Mpp2**	MAGUK p55 subfamily member 2	0.83
**Ahcy**	S-adenosylhomocysteine hydrolase	0.83
**Cadm3**	Cell adhesion molecule 3	0.83
**Qdpr**	Dihydropteridine reductase	0.84
**Aldoc**	Aldolase C, fructose-bisphosphate	0.85
**Grb2**	Growth factor receptor-bound protein 2	0.86
**Uqcrc1**	Cytochrome b-c1 complex subunit 1, mitochondrial	1.10
**Nars**	Asparagine--tRNA ligase, cytoplasmic	1.12
**Rac1**	Ras-related C3 botulinum toxin substrate 1	1.12
**Ndufb7**	NADH dehydrogenase [ubiquinone] 1 beta subcomplex subunit 7	1.14
**Pgam1**	Phosphoglycerate mutase 1	1.14
**Nfu1**	NFU1 iron-sulfur cluster scaffold homolog, mitochondrial	1.14
**Pafah1b2**	Platelet-activating factor acetylhydrolase IB subunit beta	1.15
**Camk2b**	Calcium/calmodulin-dependent protein kinase type II subunit beta	1.15
**Myl12b**	Myosin regulatory light chain 12B	1.15
**Dnm1**	Dynamin-1	1.15
**Ntm**	Neurotrimin	1.16
**Dnajc11**	DnaJ homolog subfamily C member 11	1.16
**Ppp2ca**	Serine/threonine-protein phosphatase 2A catalytic subunit alpha isoform	1.18
**Srsf1**	Serine/arginine-rich splicing factor 1	1.19
**Ppp6c**	Serine/threonine-protein phosphatase 6 catalytic subunit	1.20
**Actr3b**	Actin-related protein 3B	1.20
**Actr2**	Actin-related protein 2	1.20
**Ak5**	Adenylate kinase isoenzyme 5	1.21
**Amph**	Amphiphysin	1.21
**Hspa1l**	Heat shock 70 kDa protein 1-like	1.22
**Hsp90ab1**	Heat shock protein HSP 90-beta	1.22
**Gpd1**	Glycerol-3-phosphate dehydrogenase 1 (soluble)	1.22
**Cfl1**	Cofilin-1	1.22
**Trim2**	Tripartite motif-containing protein 2	1.23
**Nt5dc3**	5'-nucleotidase domain containing 3	1.23
**Caprin1**	Caprin-1	1.24
**Ran**	GTP-binding nuclear protein Ran	1.24

Protein	Annotation	Fold-change
**Agap2**	Arf-GAP with GTPase, ANK repeat and PH domain-containing protein 2	1.24
**Prrt3**	Proline-rich transmembrane protein 3	1.24
**Shank2**	SH3 and multiple ankyrin repeat domains protein 2	1.24
**Arpc1a**	Actin-related protein 2/3 complex subunit 1A	1.25
**Hnrnpm**	Heterogeneous nuclear ribonucleoprotein M	1.27
**Efhd2**	EF-hand domain-containing protein D2	1.27
**Myo18a**	Unconventional myosin-XVIIIa	1.28
**Pip4k2c**	Phosphatidylinositol 5-phosphate 4-kinase type-2 gamma	1.28
**Lancl1**	LanC-like protein 1	1.29
**Paip1**	Polyadenylate-binding protein-interacting protein 1	1.29
**Arpc3**	Actin-related protein 2/3 complex subunit 3	1.30
**Scrn1**	Secernin-1	1.33
**Hmgcs1**	Hydroxymethylglutaryl-CoA synthase, cytoplasmic	1.34
**Gnl1**	Guanine nucleotide-binding protein-like 1	1.35
**Ndufs8**	NADH dehydrogenase [ubiquinone] iron-sulfur protein 8, mitochondrial	1.43
**Hars**	Histidine--tRNA ligase, cytoplasmic	1.48
**Rpl10a**	60S ribosomal protein L10a	1.50
**Nme2**	Nucleoside diphosphate kinase B	1.51
**Lars**	Leucine--tRNA ligase, cytoplasmic	1.52
**Rps29**	Ribosomal protein S29	1.54
**Apmap**	Adipocyte plasma membrane-associated protein	1.66
**Acadvl**	Very long-chain specific acyl-CoA dehydrogenase, mitochondrial	1.74
**Dyx1c1**	Dynein assembly factor 4, axonemal	1.83
**Tpm4**	Tropomyosin alpha-4 chain	2.00

Supplementary Table 3. List of proteins that are commonly uniquely altered in both midbrain and prefrontal cortex of MGO injected Thy1-aSyn. Mouse proteins nomenclature is used.

Protein	Annotation	Fold-change	
		Midbrain	Cortex
**Camk2b**	Calcium/calmodulin-dependent protein kinase type II subunit beta OS	1,98	1,15
**C1qbp**	Complement component 1 Q subcomponent-binding protein, mitochondrial OS	1,48	0,69
**Agap2**	Arf-GAP with GTPase, ANK repeat and PH domain-containing protein 2 OS	1,76	1,24
**Pafah1b2**	Platelet-activating factor acetylhydrolase IB subunit beta OS	1,66	1,15
**Actr3b**	Actin-related protein 3B OS	1,62	1,21
**Cdh2**	Cadherin-2 OS	1,61	0,71
**Prrt3**	Proline-rich transmembrane protein 3 OS	1,56	1,25
**Rtn1**	Reticulon-1 OS	1,35	0,83
**Actr2**	Actin-related protein 2 OS	1,47	1,21
**Ppp3r1**	Calcineurin subunit B type 1 OS	1,60	0,76
**Myl12b**	Myosin regulatory light chain 12B OS	1,26	1,16
**Apmap**	Adipocyte plasma membrane-associated protein OS	0,61	1,83
**Shank2**	SH3 and multiple ankyrin repeat domains protein 2 OS	1,86	1,27
**Hnrnpm**	Heterogeneous nuclear ribonucleoprotein M OS	1,35	1,28
**Ahcy**	Adenosylhomocysteinase OS	1,28	0,83
**Srsf1**	Serine/arginine-rich splicing factor 1 OS	1,30	1,20
**Mpp2**	MAGUK p55 subfamily member 2 OS	1,69	0,83
**Jakmip3**	Janus kinase and microtubule-interacting protein 3 OS	1,98	0,54

Supplementary Table 4. List of proteins that are altered in the midbrain of MGO-injected WT mice. Mouse proteins nomenclature is used.

Protein	Fold-change	Protein	Fold-change	Protein	Fold-change	Protein	Fold-change
Gltp	0.19	Ptpn9	1.52	Gad2	1.24	Calm3	1.23
Uqcrh	2.54	Iars2	1.32	Hba	0.33	Atp5pb	0.35
Cnn3	1.67	Twf1	0.79	Plcl1	1.59	Rpl7	1.19
Ndufs2	0.60	Snca	1.60	Pafah1b1	0.81	Rab3b	0.22
Ank3	0.51	Ndufa1	1.43	Rpl10	1.44	Cfl1	1.37
Cltc	1.47	Rock2	1.40	Slc6a1	1.51	Rpl24	1.15
Ppp2ca	1.95	Cacybp	0.75	Pgam1	1.22	Cdc42	1.55
Grb2	0.53	Ndufa4	0.69	Ahsa1	1.62	Pgk1	1.17
Septin2	1.49	Nebl	2.05	Tstd3	0.55	Yars	0.77
Atic	0.40	Sri	0.69	Prxl2b	0.72	Tcea1	0.10
Psmd3	1.72	Prpsap1	1.41	Vcan	1.30	Eif4g3	1.64
Cops8	2.03	Ptbp2	2.16	Ndufa6	1.29	Gyg1	0.77
Fabp5	0.51	Copa	0.73	Pcca	0.49	Pramel7	0.40
Eif5	1.39	Dynlrb1	1.34	Mtnd1	0.57	St13	0.40
Hars	0.37	Ephx3	1.66	Dad1	0.68	Sipa1	0.62
Kif5b	2.20	Rplp0	1.48	Abracl	1.26	Fubp1	1.25
Arhgap35	5.35	Nbea	1.60	Klc1	1.60	Suclg1	1.21
Got2	0.67	Prdx3	1.39	Mtpn	1.34	Rdx	1.23
Tuba1a	1.87	Copg2	1.33	Nod2	0.78	Nudc	1.44
Eef1g	1.45	Clpp	0.64	Prdx6	0.75	Vta1	1.22
Zfyve26	1.60	Syt12	1.87	Rpl22	0.64	Cisd1	0.47
Txndc17	1.50	Gja1	0.62	Pspc1	1.65	Eef1a1	1.20
Nol3	1.58	Ppa2	0.75	Scrn3	1.37	Fntb	5.34
Gng7	0.62	Phgdh	1.34	Glrx5	1.28	Akr7a2	2.27
Agt	1.86	Psmd11	1.37	Acaa2	1.34	Nras	1.14
Cltb	1.87	Slc25a51	0.54	Myadm	1.94	Ndufb9	0.55
Hspa1l	1.80	Dpp6	1.38	Kif15	0.22	Shank3	1.57
Pcyox1	2.10	Ndufs4	0.37	Psmd5	0.83	Rpl38	1.37
Ehd4	1.57	Auh	1.37	Psma3	1.37	Acbd6	5.25
Cox5a	1.28	Tppp	1.33	Wbp2	0.64	Aco2	0.75
Aldh2	1.49	Ndufb7	1.29	Psmc6	1.24	Pgm1	0.74
Rab35	1.60	Psap	0.49	Ndufa13	1.29	Sarnp	1.17
Hacd3	0.72	Vamp2	1.50	Ywhag	0.51	Eif3b	0.44
Penk	0.33	Krt10	0.50	Tardbp	1.34	Cyc1	1.14
Rars	1.59	Timm9	0.66	Cpm	2.68	Pdcd5	1.18
Septin9	1.93	Arl3	1.38	Pcp4	1.50	Chchd6	0.86
Retreg2	2.35	Fh	1.21	Ppp2r1a	1.22	Glrx3	1.38
Hadhb	0.47	Cul3	0.53	Idh2	0.70	Ddc	1.44

Protein	Fold-change	Protein	Fold-change	Protein	Fold-change	Protein	Fold-change
Adck1	1.47	Abcf2	2.09	Taldo1	1.31	Tagln	1.98
Pfn1	1.48	Capn2	1.25	Hspa9	1.23	Tspan2	0.49
Scrn1	1.60	Tpm4	0.33	Pde2a	1.44	Atp1a3	0.87
Ap3d1	1.86	Arpc5l	1.46	Uqcr11	0.58	Mapk10	0.83
Hadh	1.94	Set	1.28	Nudt2	1.34	Carhsp1	1.25
Grm2	2.59	Aak1	1.27	Pitpna	1.56	Dlg4	0.75
Ociad2	1.69	Lancl1	0.53	Cndp2	0.56	Hibadh	1.20
Arhgef19	1.47	Snap47	0.52	Rpl23a	0.50	Ywhaq	0.48
Mapre2	1.37	Gars	1.39	Snrpg	1.97	Idh3g	0.86
Gdi2	0.59	Nckipsd	1.22	Nedd8	1.52	Mdh1	0.53
Ppid	1.43	Hprt1	0.75	Cbr3	0.58	Rexo2	1.21
Kcna2	0.65	Atp5f1e	0.41	Mtch2	0.29	Ckmt1	0.82
Rida	0.55	Atpaf1	0.55	Cacna2d2	1.30	Unc13a	1.38
Atp2a2	1.29	Vps35	0.81	Trmt112	1.31	Rab14	1.19
Bphl	3.67	Rpl9	1.58	Ly6h	0.61	Prxl2a	1.19
Sparcl1	2.18	Esyt1	1.76	Bcas1	0.67	Tagln3	1.16
Samm50	0.40	Stxbp1	1.20	Cycs	0.84	Rpl26	0.68
Kif5c	1.79	Gnaz	0.70	Ttc7b	1.68	Adk	0.56
Prkar1a	1.42	Scamp5	0.33	Wasf1	1.54	Ctnnb1	1.19
Ldha	0.68	Gcsh	1.41	Cct2	1.33	Atp6v1g1	0.61
Rab3a	1.63	Ermn	1.63	Pea15	0.69	Capza2	1.25
Rab5b	1.39	Ddx6	1.23	Aplp2	0.36	Atad3	0.54
Mtx1	2.18	Nme1	1.61	Ptprs	1.60	Dlgap1	1.90
Ctsd	1.35	Hspe1	1.41	Dnajc11	1.31	Snrpd3	1.20
Cplx1	1.48	Pfkp	1.22	Ahcyl1	1.42	Gpd2	1.21
Coasy	1.93	Xpo1	1.45	Dars	1.24	Alyref	0.36
Atp6ap1	0.41	Ppp6c	1.34	Otud6b	0.68	Gdpd1	0.51
Lamp1	0.37	Tspan7	0.33	Mpi	1.17	Bcat1	1.26
Ndufb5	0.74	Strap	1.68	Tppp3	1.37	Mcat	2.23
Cnp	0.69	Lasp1	1.30	Lypla2	1.58	Psmc1	1.34
Rpl27a	0.57	Nutf2	1.36	Slc8a2	1.85	Vapb	1.32
Vamp1	0.42	Slc25a4	0.82	Mapre1	1.28	Aldh1a1	1.29
Ntrk2	1.61	Tufm	1.34	Hdgfl3	1.87	Sv2a	1.21
Ppp1cc	1.57	Ndufs8	1.56	Echs1	0.80	Rpl15	0.62
Pitpnc1	1.56	Rps12	0.70	Syngr3	0.63	Pip4k2c	1.42
Ndufa12	1.55	Ndufb1	0.35	Tpd52l2	0.86	Arhgap44	1.65
Adam23	0.50	Ivd	0.83	Ldhb	0.78	Praf2	0.73
Gng2	0.70	Rpl30	0.58	Txnrd1	1.21	Cct7	1.20
Ddt	1.56	Rpl23	1.32	Ndufab1	0.63	Acsl6	1.14
Rpl35	1.71	Cops5	0.58	Rps3a	1.23	Krt75	2.18

Protein	Fold-change	Protein	Fold-change	Protein	Fold-change	Protein	Fold-change
**Ctnnd2**	1.37	**Rps10**	0.78	**Atp6v0d1**	0.71	**Ppm1a**	0.82
**Mpc2**	0.65	**Ogdh**	0.76	**Cadm3**	0.87	**Ptpmt1**	1.27
**Ap1m1**	1.56	**Acaa1a**	3.05	**Ptpa**	0.74	**Pdcd6**	1.31
**Slc27a4**	1.87	**Hnrnpc**	0.65	**Gstp1**	1.19	**Zc3h15**	5.52
**Fxyd6**	1.45	**Usp5**	1.17	**Ap1s2**	0.59	**Anxa7**	1.25
**Rpl27**	0.61	**Cnrip1**	1.30	**Sncb**	1.25	**Vps26a**	0.66
**Pclo**	2.01	**Atp5pf**	0.59	**Pdcd11**	2.81	**Tcp1**	1.23
**Me2**	1.76	**Gucy1b1**	1.58	**Ykt6**	1.38	**Aldh9a1**	1.31
**Ndufs5**	1.38	**Coro1c**	1.33	**Oxr1**	1.36	**Gabra1**	1.46
**H1f0**	1.46	**Cab39**	1.54	**Bag6**	0.46	**H2afv**	0.59
**Appl1**	2.15	**Ywhah**	0.62	**Slc25a12**	1.19	**Syt1**	1.28
**Gmps**	1.22	**Itgb1**	0.70	**Ndufv2**	1.22	**Cherp**	1.94
**Rps28**	1.52	**Psmb5**	0.56	**Aldh1l1**	1.28	**Afg3l2**	0.89
**Atp6v1g2**	1.42	**Lnpk**	0.42	**Negr1**	1.23	**Hnrnpd**	0.63
**Tsn**	0.73	**Cirbp**	2.06	**Arpc3**	1.24	**Nae1**	0.50
**Rab10**	1.38	**Rack1**	1.56	**Krt85**	1.44	**Hsd17b4**	1.17
**Pls1**	0.40	**Atp6v1a**	1.15	**Gstm7**	2.00	**Napb**	0.55
**Rab7a**	1.39	**Lancl2**	0.63	**Diras2**	1.40	**Aldh6a1**	0.87
**Gphn**	0.63	**Rcn2**	1.58	**Acot7**	0.66	**Pdxk**	0.75
**Park7**	1.28	**Abcd3**	1.32	**Cul2**	1.52	**Maoa**	0.87
**Cplx2**	1.46	**Slc6a11**	0.39	**Asna1**	1.39	**Atp6v1f**	0.42
**Pdk3**	0.80	**Epg5**	7.40	**Ube2v1**	0.38	**Mapk8ip3**	0.79
**Chmp6**	0.47	**Atp5f1a**	1.34	**Ca2**	0.83	**Mmp25**	2.11
**Rplp1**	1.33	**Slc20a2**	1.76	**Wars**	0.78	**Sptb**	1.33
**Fbxo43**	1.33	**Glod4**	0.78	**Hnrnph1**	1.37	**Ppp3ca**	1.26
**L1cam**	1.57	**Eif5a**	1.71	**Tomm22**	1.38	**Plcb1**	0.67
**Uqcrfs1**	1.46	**Fscn1**	1.27	**Smyd5**	1.34	**Epm2aip1**	1.30
**Sdhb**	1.21	**Farsa**	1.97	**Smap1**	1.22	**Actr1a**	1.23
**Cops6**	1.46	**Vdac2**	0.84	**Etfa**	1.15	**Cox5b**	0.20
**Tmem74**	0.42	**Me1**	1.28	**Psma4**	1.29	**Huwe1**	1.43
**Rab1A**	1.43	**Surf4**	0.73	**Vps4a**	1.35	**Ube2m**	1.32
**Fmnl1**	1.89	**Gpd1**	1.38	**Sult4a1**	0.62	**Tfap2e**	1.41
**Rps15a**	0.63	**Dctn2**	1.37	**Prdx1**	0.80	**Cox4i1**	0.76
**Aspa**	0.67	**Cpne5**	1.47	**Slc25a18**	0.45		
**Ap1s1**	1.69	**Tbcc**	1.37	**Twf2**	1.45		
**Dnajb2**	1.70	**Actn1**	1.26	**Pin1**	1.23		
**Sirpa**	1.39	**Dpysl4**	1.18	**Gdap1**	0.76		

Supplementary Table 5. List of proteins that are altered in the prefrontal cortex of MGO-injected WT mice. Mouse proteins nomenclature is used.

Protein	Fold-change	Protein	Fold-change	Protein	Fold-change	Protein	Fold-change
Vim	1.53	Ranbp1	1.27	Psd3	1.23	Mcrs1	1.82
Ube2n	0.72	Acot7	0.61	Anxa5	1.40	Nae1	0.82
Hadh	1.75	Camkv	0.80	Napg	1.15	Slc44a1	1.61
Gfap	2.01	Svip	2.99	Efr3b	0.54	Akap7	2.62
Psmb7	0.66	Glrx3	1.28	Myo5a	0.86	Prps1	3.23
Slc6a17	1.59	Hnrnpu	1.26	Atp2b2	0.86	Eno2	0.84
Nceh1	0.73	Vgf	0.67	Plxna4	0.74	Ehd3	1.17
Dip2a	0.56	Fntb	9.47	Hadhb	1.82	Agap3	1.23
Rdx	1.21	Acot13	1.26	Cacna1e	0.55	Prkcb	0.81
Git1	0.76	Hnrnpc	1.32	Necap1	0.75	Aars	0.71
Vcp	0.84	Esd	0.77	Fdps	0.76	Rcc2	0.56
Ppp1cb	0.64	Anxa7	1.24	Sdhc	1.20	Atp6v0a1	1.13
Snrpg	1.44	Kcnab2	0.66	Copg2	0.81	Fah	0.22
Hebp1	0.75	Ckb	1.29	Epha4	0.83	Acbd6	1.30
Gucy1b1	1.72	Grin2b	0.77	Atp1a3	1.21	Rtcb	1.17
Purb	3.97	Gstm5	1.21	Atp5pf	1.24	Wars	0.67
Hspa4	0.84	Etfb	1.24	Tspan7	0.52	Plpbp	1.21
Septin7	0.78	Arf3	0.42	Dpp10	0.80	Dnm1l	0.71
Scamp1	2.06	Cul3	0.57	Lap3	1.34	Pde1b	0.68
Uqcrh	1.35	Gng2	0.75	Ywhah	0.71	Ube2k	0.77
Dnajb2	0.72	Prdx2	1.22	Agk	1.15	Add2	0.87
Ap1g1	2.13	Grin1	0.67	Pitpna	1.64	Slc17a7	0.81
Fam49b	0.84	Abi2	0.52	Mrps22	0.68	Tollip	1.10
Fabp3	1.38	Gmps	1.30	Ethe1	1.65	Rap1b	0.77
Oxct1	0.79	Canx	1.22	Prune1	0.71	Got2	0.90
Atp6v1f	0.72	Akr1b1	1.19	Ndufs4	0.78	Csnk2a1	0.82
Samm50	0.44	Syt1	1.20	Nptn	1.26	Ddb1	0.75
Atp6v0d1	0.77	Dnaja1	0.51	Pgm3	0.28	Wdr37	0.76
Cbr3	1.79	Cpne5	0.80	Vbp1	0.76	Atp5pd	1.11
Uchl1	1.35	Dynll1	1.19	Oxr1	1.46	Vdac3	0.85
Cycs	1.28	Chgb	0.40	Rack1	1.28	Rab5c	0.61
Selenbp2	1.53	Rimbp2	0.58	Rps8	0.90	Arhgap32	0.73
Rps5	4.58	Pls3	1.33	Basp1	1.21	Ywhag	0.83
Hibch	1.30	Lsamp	0.85	Bag6	0.55	Prpsap2	1.79
Septin6	0.80	Atp6ap1	0.74	Chchd3	0.88	Acp1	0.65
Rps28	1.33	Krt77	1.97	Atp5mf	1.11	Tomm22	0.79
Acyp2	2.28	Rpl28	1.19	Sacm1l	1.22	Atad3	0.66
Sfxn3	1.22	Acadl	0.74	Ist1	1.46	Fech	0.71

Protein	Fold-change	Protein	Fold-change	Protein	Fold-change	Protein	Fold-change
**Mthfd1**	1.53	**Eif3e**	0.69	**Ndufab1**	0.75	**Cplx1**	1.28
**Psmd12**	0.85	**Lypla2**	1.50	**Gk**	2.07	**Dcaf15**	0.50
**Grhpr**	1.26	**Septin11**	1.56	**Krt75**	3.55	**Vps51**	0.45
**Prep**	0.58	**Cnn3**	1.42	**Sh3bgrl3**	0.67	**Hbb-b2**	0.34
**Ca2**	0.64	**Atp1b1**	1.19	**Akap12**	1.93	**Lypla1**	1.52
**Tubb4a**	0.88	**Cds2**	1.25	**Cntnap2**	0.83	**Pak3**	0.64
**Cdc42**	1.75	**Kcnc3**	0.69	**Ppia**	0.81	**Etfa**	1.16
**Pacs1**	0.69	**Adcy2**	0.62	**Hspa5**	1.14	**Calm3**	1.29
**Ptpa**	0.75	**Ndufa12**	1.21	**Aco1**	0.77	**Tuba4a**	0.81
**Cct3**	1.29	**Decr1**	1.17	**Tceal5**	2.80	**Ndufa8**	1.19
**Pi4ka**	0.71	**Cs**	1.16	**Aldh2**	1.18	**Slc1a3**	1.22
**Atox1**	1.28	**Sccpdh**	0.86	**Slc27a4**	1.90		
**Mras**	1.49	**Cyfip2**	0.69	**Wwc1**	0.69		
**Dlgap1**	3.48	**Clic4**	1.43	**App**	0.81		

Supplementary Table 6. List of proteins that are commonly altered by MGO (MGO-WT vs Veh-WT) and aSyn (Veh-Thy1-aSyn vs Veh-WT) in the midbrain. Mouse proteins nomenclature is used.

Protein	Fold-change MGO vs Veh	Fold-change Thy1 vs WT	Protein	Fold-change MGO vs Veh	Fold-change Thy1 vs WT
Gltp	0.19	0.32	Rps12	0.70	0.79
Uqcrh	2.54	2.30	Cab39	1.54	1.28
Ndufs2	0.60	0.83	Itgb1	0.70	0.72
Ank3	0.51	0.52	Psmb5	0.56	0.86
Ppp2ca	1.95	1.31	Rack1	1.56	1.36
Grb2	0.53	0.56	Glod4	0.78	0.86
Septin2	1.49	1.12	Surf4	0.73	0.69
Cops8	2.03	1.17	Actn1	1.26	1.52
Eef1g	1.45	1.40	Ahsa1	1.62	1.31
Hacd3	0.72	0.82	Tstd3	0.55	0.55
Septin9	1.93	1.55	Pcca	0.49	0.48
Ap3d1	1.86	1.28	Mtpn	1.34	1.25
Gdi2	0.59	0.83	Myadm	1.94	1.67
Bphl	3.67	5.21	Kif15	0.22	0.07
Sparcl1	2.18	1.62	Wbp2	0.64	0.56
Kif5c	1.79	1.38	Taldo1	1.31	1.45
Atp6v1g2	1.42	1.31	Mtch2	0.29	0.31
Pls1	0.40	0.36	Cacna2d2	1.30	1.28
Chmp6	0.47	0.54	Ly6h	0.61	0.66
Uqcrfs1	1.46	1.35	Pea15	0.69	0.55
Sdhb	1.21	1.23	Bag6	0.46	0.44
Tmem74	0.42	0.33	Arpc3	1.24	1.34
Snca	1.60	7.71	Krt85	1.44	2.06
Ndufa4	0.69	0.74	Smyd5	1.34	1.46
Rplp0	1.48	1.63	Rpl7	1.19	1.23
Prdx3	1.39	1.23	Rpl24	1.15	1.18
Clpp	0.64	0.85	Fntb	5.34	2.76
Syt12	1.87	1.91	Tagln	1.98	1.60
Timm9	0.66	0.82	Atp1a3	0.87	0.80
Fh	1.21	1.11	Mapk10	0.83	0.82
Capn2	1.25	1.20	Alyref	0.36	0.25
Snap47	0.52	0.39	Afg3l2	0.89	0.84
Atp5f1e	0.41	0.55	Napb	0.55	0.82
Atpaf1	0.55	0.56	Aldh6a1	0.87	0.85
Vps35	0.81	0.81	Pdxk	0.75	0.77
Slc25a4	0.82	0.87	Plcb1	0.67	0.54

Supplementary Table 7. List of proteins that are glycated in the midbrain of MGO-injected Thy1-aSyn mice. Mouse proteins nomenclature is used.

Protein	Protein	Protein	Protein	Protein	Protein	Protein	Protein
Aadat	C2orf42	Dnajc13	Hadh	Mbp	Pbrm1	Rpl24	Tigd5
Abca2	C6orf132	Dnajc25	Hap1	Mcm6	Pcca	Rpl26l1	Tjp1
Abcb10	Cacna1i	Dnajc9	Hck	Mdm1	Pcsk6	Rpl27	Tlx3
Abhd16a	Cacna1s	Dnm1l	Hells	Mdn1	Pde1b	Rpl7	Tmem201
Acan	Cadm2	Dock5	Herc4	Mecp2	Pde4dip	Rpl7a	Tmem216
Acod1	Cadm4	Dpysl3	Hgs	Med24	Pdia3	Rpl8	Tmprss6
Acsl1	Cadps2	Drp2	Hist1h4a	Mff	Pdzd3	Rps24	Tnfrsf10a
Acsl6	Calb2	Dsp	Hmcn2	Mfhas1	Pfkfb1	Rps6	Tnn
Actn3	Camk2a	Dst	Hnrnpk	Mfsd6l	Pfkp	Rrbp1	Tnxb
Adam22	Camk2b	Dtna	Hsp90aa1	Mib2	Pgm1	Rrp8	Toml12
Adgrl2	Camk2d	Dync1h1	Hsp90ab1	Mical2	Pgp	Rrs1	Top1mt
Agap3	Camk2g	Ebf1	Hspb1	Mmrn1	Phf24	Rtel1	Trappc10
Agrn	Cand1	Echdc2	Htra1	Mov10l1	Phf3	Rtn3	Trav38-2dv8
Ahcyl1	Cask	Eddm13	Htra3	Mphosph10	Phlda1	Ruvbl2	Trim28
Ahcyl2	Caskin1	Efhd2	Hydin	Mrpl47	Phlda3	Ryr3	Trim39
Aifm3	Cat	Eftud2	Idua	Mrps18b	Pick1	S1pr1	Tro
Akr1b10	Cavin3	Egr3	Igsf3	Mrps26	Pik3ca	Sash1	Trrap
Aldh1b1	Ccdc102a	Eif3a	Immt	Ms4a5	Pik3r1	Sash3	Tsks
Aldh2	Ccdc120	Eif4h	Ina	Mtmr9	Pikfyve	Scaf11	Ttc39b
Aldh6a1	Ccdc60	Eloa3	Inppl1	Myh9	Pitpna	Scmh1	Ttf2
Aldoa	Ccdc66	Elovl6	Ints6l	Myo18a	Plagl2	Scn2a	Ttn
Aldoc	Ccdc77	Elp1	Ipo5	Myo3a	Plcb1	Sdk1	Tubb
Alg6	Ccdc88b	Eno1	Ipp	Myo5a	Plec	Sdr42e2	Tubb2a
Alg8	Ccdc91	Epha7	Iqcd	Myo5b	Plekhg3	Sec14l1	Tubb2b
Alms1	Cd2ap	Erbb3	Iqcm	Myo9b	Plxnb1	Sema4c	Tubb4a
Amer2	Cdc123	Ercc6l	Iqsec1	Myom2	Pomgnt1	Serbp1	Tubb4b
Ampd2	Cdc14a	Ercc6l2	Iqsec2	Mysm1	Ppfia1	Setbp1	Tulp2
Ank3	Cdc7	Erich3	Iscu	Nadk2	Ppfia4	Sgip1	Tyw3
Ankrd17	Cdh5	Ervk-19	Isg20	Nbea	Ppp1cb	Sh3gl1	Ube2v2
Ankrd63	Cdhr1	Esyt2	Jakmip3	Nck2	Ppp1r12b	Sh3gl2	Ube3b
Anks1b	Cdkl5	Etfb	Jmy	Ncor2	Ppp1r12c	Shank3	Ube3d
Aoc1	Cenpf	Etfdh	Kat2b	Ndrg4	Ppp1r9b	Shld3	Upf3b
Ap1b1	Cep120	Evpl	Kcnab2	Ndufa2	Ppp2r1a	Shprh	Uqcrc1
Ap2b1	Cep162	F8	Kcnh2	Ndufs1	Ppp2r5d	Ska2	Ushbp1
App	Cep250	Fads2	Kcnh3	Ndufs2	Ppp2r5e	Skp1	Uso1
Aqr	Cep290	Fads6	Kcnq5	Ndufs5	Ppp3ca	Slc12a4	Usp17l2
Arf6	Cfap54	Fam135a	Kctd19	Nebl	Ppp3cb	Slc12a5	Usp24
Arfrp1	Cfap57	Fam173b	Khsrp	Nefh	Ppp4r3a	Slc16a8	Usp35

Protein	Protein	Protein	Protein	Protein	Protein	Protein	Protein
Arhgap21	Chd8	Fam53b	Kiaa0100	Nefl	Prag1	Slc22a2	Usp43
Arhgap23	Chtf8	Fam71e1	Kiaa1549l	Nefm	Prdm5	Slc24a4	Utp20
Arhgap28	Ckmt1a	Fam83e	Kiaa1551	Nf1	Prdx3	Slc25a5	Uts2r
Arid4b	Clip2	Farsb	Kif1a	Nf2	Prdx5	Slc2a4	Vars
Arid5b	Cltc	Fat1	Kif21a	Nfasc	Prep	Slc34a1	Vbp1
Arl6ip1	Cmas	Fat2	Kif21b	Nfatc2ip	Prim2	Slc4a3	Vcp
Arl6ip5	Cntrl	Fbxo39	Kif26a	Nin	Prkar2b	Slc7a14	Virma
Arrdc3	Coch	Fer1l6	Kif5c	Nkx6-3	Prkdc	Smarcd2	Wasf1
Asic4	Cog1	Fezf2	Kifap3	Nlrp13	Prpf3	Snap91	Wdr6
Aspm	Col16a1	Fga	Kirrel1	Nolc1	Psap	Snph	Wdsub1
Atf6	Col24a1	Fhod3	Klc3	Npm1	Psma3	Snx29	Wnk4
Atg2b	Col6a2	Fkbp8	Klhl17	Nr2c2	Psmc2	Soat1	Wnt1
Atp1a3	Col9a1	Fmnl1	Klhl2	Nrac	Ptpn11	Son	Wnt10a
Atp1b1	Colgalt1	Fn1	Krr1	Nrg2	Pum3	Spata20	Wwc3
Atp2a2	Coq8a	Fnbp1	Krt80	Nsd2	Qki	Sphk1	Yipf1
Atp2b3	Cplane1	Fscn1	Lama2	Nsd3	Rab12	Spib	Zbtb11
Atp5f1a	Crocc2	Fsip1	Lama5	Nsun7	Rab34	Spink8	Zbtb16
Atp5f1b	Cs	Fsip2	Lca5	Nucks1	Ralgapa2	Sptan1	Zbtb21
Atp5f1c	Cspg5	Fxyd1	Ldlrap1	Nudcd3	Rasa2	Sptbn1	Zc3h7a
Atp6v0a2	Ctnnbl1	Gal3st2	Lmtk2	Obscn	Rb1cc1	Sptbn5	Zdhhc8
Atp6v1c1	Ctsf	Gart	Lonrf3	Ocrl	Rbm12b	Spz1	Zfhx4
Atp6v1g2	Cyp27a1	Gas2l3	Lrig3	Ogdh	Rbm19	Srrm2	Zmym2
Atp8a1	Ddhd2	Gatd3a	Lrp1	Ogfr	Rbmx	Srrm3	Znf195
Atp9a	Ddrgk1	Ghrh	Lrp2	Omg	Rdh14	Srsf11	Znf407
Avpi1	Ddx3x	Gli3	Lrrc74a	Ophn1	Reep1	Srsf3	Znf491
Best3	Ddx4	Glud1	Lrrc8a	Or10j1	Rep15	Srsf7	Znf516
Bet1	Def6	Gna14	Lrrd1	Or4c3	Rev3l	Ssna1	Znf629
Bfsp1	Dennd4c	Gng3	Lyrm4	Or52h1	Rgs9	St13p5	Znf672
Brap	Depdc5	Gnptab	Lzts3	Osbpl5	Rhbdf1	Stk11ip	Znf681
Brf2	Dgat2l7p	Golga3	Macf1	Otol1	Rif1	Suclg1	Znf707
Brinp2	Dgkh	Gphn	Maged4	Otud7a	Riok1	Suclg2	Znf723
Brms1	Dgkk	Gpi	Maoa	Ovos1	Riox1	Syne1	Znf724
Brms1l	Dhx16	Grasp	Map1a	Oxct1	Rmnd1	Syt1	Znf726
Bsn	Dhx9	Grhl3	Map2	Oxsm	Rnf44	Taf1l	Znf764
Btbd11	Dip2c	Gripap1	Map9	Oxsr1	Rock2	Tas2r7	Znf804b
C10orf113	Dlat	Grk3	Mapre2	Pabpc4	Rph3a	Tcaf1	Znf806
C11orf88	Dnah10	Gucy2d	Mast1	Pacs1	Rpia	Tcirg1	Znf816
C12orf66	Dnah8	H1fx	Mast4	Pacsin3	Rpl14	Tenm4	
C12orf74	Dnah9	H2afy	Mat2a	Pam	Rpl15	Tfap2b	
C22orf42	Dnaja2	Habp4	Matn4	Parp4	Rpl19	Tiam2	

Supplementary Table 8. List of proteins that are exclusively glycated in the midbrain of MGO-injected Thy1-aSyn mice. Mouse proteins nomenclature is used.

Protein	Protein	Protein	Protein	Protein	Protein	Protein	Protein
Aadat	Cacna1s	Elovl6	Hells	Mfsd6l	Pfkfb1	S1pr1	Trim39
Abcb10	Cadm2	Ercc6l	Hgs	Mib2	Pgm1	Sash3	Tro
Abhd16a	Calb2	Ercc6l2	Hmcn2	Mical2	Pgp	Scmh1	Tsks
Acan	Cavin3	Erich3	Hspb1	Mmrn1	Phlda1	Sdk1	Ttc39b
Acod1	Ccdc66	Esyt2	Htra1	Mov10l1	Pik3ca	Sdr42e2	Tulp2
Acsl1	Ccdc77	Etfb	Htra3	Mphosph10	Pik3r1	Sema4c	Tyw3
Actn3	Ccdc91	Etfdh	Idua	Mrpl47	Pikfyve	Sh3gl1	Ube3d
Adam22	Cd2ap	Evpl	Igsf3	Mrps18b	Plagl2	Shank3	Upf3b
Adgrl2	Cdc123	F8	Immt	Ms4a5	Plekhg3	Shld3	Uqcrc1
Agrn	Cdc14a	Fam135a	Ipp	Mtmr9	Pomgnt1	Shprh	Usp17l2
Ahcyl1	Cdc7	Fam173b	Iqcd	Myo9b	Ppp1r12b	Ska2	Usp35
Aifm3	Cdh5	Fam71e1	Iqcm	Mysm1	Ppp1r12c	Slc12a4	Virma
Aldoc	Cdhr1	Fam83e	Iscu	Nadk2	Ppp2r1a	Slc16a8	Wdr6
Alg6	Cdkl5	Farsb	Isg20	Nbea	Prag1	Slc22a2	Wdsub1
Alg8	Cenpf	Fat1	Jakmip3	Nck2	Prdx3	Slc24a4	Wnk4
Amer2	Cep120	Fat2	Kat2b	Ncor2	Prim2	Slc34a1	Wnt1
Ankrd63	Cep162	Fbxo39	Kcnab2	Ndrg4	Prkdc	Slc4a3	Wnt10a
Anks1b	Cfap54	Fer1l6	Kcnh3	Ndufa2	Prpf3	Slc7a14	Wwc3
Aoc1	Chd8	Fhod3	Kcnq5	Nefl	Psap	Smarcd2	Yipf1
Aqr	Chtf8	Fkbp8	Kctd19	Nf2	Psma3	Snap91	Zbtb11
Arf6	Cmas	Fmnl1	Khsrp	Nfatc2ip	Psmc2	Soat1	Zbtb16
Arfrp1	Cog1	Fnbp1	Kiaa0100	Nkx6-3	Ptpn11	Son	Zbtb21
Arhgap23	Col16a1	Fscn1	Kiaa1549l	Nlrp13	Pum3	Spata20	Zdhhc8
Arhgap28	Col24a1	Fxyd1	Kiaa1551	Nolc1	Qki	Sphk1	Zfhx4
Arid4b	Col6a2	Gal3st2	Kif1a	Nr2c2	Rasa2	Spib	Zmym2
Arid5b	Colgalt1	Gart	Kif5c	Nsd3	Rb1cc1	Spink8	Znf195
Arl6ip1	Coq8a	Gas2l3	Kirrel1	Nsun7	Rbmx	Spz1	Znf407
Arl6ip5	Ctnnbl1	Gatd3a	Klc3	Nucks1	Rdh14	Srrm3	Znf491
Arrdc3	Ctsf	Ghrh	Klhl17	Nudcd3	Reep1	Srsf11	Znf516
Atf6	Cyp27a1	Gli3	Krr1	Ogdh	Rep15	Syt1	Znf629
Atg2b	Ddhd2	Glud1	Lama2	Ogfr	Rev3l	Taf1l	Znf672
Atp2a2	Ddx3x	Gna14	Lama5	Ophn1	Rgs9	Tas2r7	Znf681
Atp5f1c	Ddx4	Gnptab	Ldlrap1	Or4c3	Rif1	Tcaf1	Znf707
Atp6v0a2	Dgkk	Golga3	Lmtk2	Or52h1	Riok1	Tenm4	Znf723
Atp9a	Dip2c	Gphn	Lrig3	Osbpl5	Rnf44	Tfap2b	Znf726
Avpi1	Dlat	Gpi	Lrrc74a	Otol1	Rph3a	Tigd5	Znf764
Best3	Dnaja2	Grasp	Lrrc8a	Otud7a	Rpl14	Tjp1	Znf804b
Bet1	Dnajc13	Grhl3	Lrrd1	Oxsr1	Rpl26l1	Tlx3	Znf806

Protein	Protein	Protein	Protein	Protein	Protein	Protein	Protein
Bfsp1	Dnajc9	Gripap1	Maoa	Pabpc4	Rpl27	Tmem201	Znf816
Brms1l	Dock5	Grk3	Map9	Pacsin3	Rpl7a	Tmem216	
Btbd11	Ebf1	Gucy2d	Matn4	Parp4	Rrbp1	Tmprss6	
C10orf113	Eddm13	H1fx	Mcm6	Pcca	Rrs1	Tnfrsf10a	
C11orf88	Eftud2	Hadh	Mdm1	Pde1b	Rtel1	Tnn	
C22orf42	Egr3	Hap1	Med24	Pde4dip	Rtn3	Top1mt	
Cacna1i	Eif4h	Hck	Mfhas1	Pdzd3	Ruvbl2	Trappc10	
