# Supplementary material for: Glycation modulates glutamatergic signaling and exacerbates Parkinson’s disease-like phenotypes
Source: NPJ Parkinsons Dis. 2022 Apr 25;8:51. doi: 10.1038/s41531-022-00314-x (PMC9038780; doi:10.1038/s41531-022-00314-x)
Supplement: Supplementary file 2 — Reporting Summary [file 41531_2022_314_MOESM2_ESM.pdf]

nature portfolio

Corresponding author(s): Hugo Vicente Miranda, Tiago Outeiro

Last updated by author(s): Feb 4, 2022

Reporting Summary

Nature Portfolio wishes to improve the reproducibility of the work that we publish. This form provides structure for consistency and transparency in reporting. For further information on Nature Portfolio policies, see our Editorial Policies and the Editorial Policy Checklist.

Statistics

For all statistical analyses, confirm that the following items are present in the figure legend, table legend, main text, or Methods section.

n/a	Confirmed	
☐	☐	The exact sample size (*n*) for each experimental group/condition, given as a discrete number and unit of measurement
☐	☒	A statement on whether measurements were taken from distinct samples or whether the same sample was measured repeatedly
☐	☒	The statistical test(s) used AND whether they are one- or two-sided<br>*Only common tests should be described solely by name; describe more complex techniques in the Methods section.*
☐	☒	A description of all covariates tested
☐	☒	A description of any assumptions or corrections, such as tests of normality and adjustment for multiple comparisons
☐	☒	A full description of the statistical parameters including central tendency (e.g. means) or other basic estimates (e.g. regression coefficient) AND variation (e.g. standard deviation) or associated estimates of uncertainty (e.g. confidence intervals)
☐	☒	For null hypothesis testing, the test statistic (e.g. F, t, r) with confidence intervals, effect sizes, degrees of freedom and P value noted<br>*Give P values as exact values whenever suitable.*
☒	☐	For Bayesian analysis, information on the choice of priors and Markov chain Monte Carlo settings
☒	☐	For hierarchical and complex designs, identification of the appropriate level for tests and full reporting of outcomes
☒	☐	Estimates of effect sizes (e.g. Cohen's *d*, Pearson's *r*), indicating how they were calculated

Our web collection on statistics for biologists contains articles on many of the points above.

Software and code

Policy information about availability of computer code

Data collection	PeakView 2.2 (Sciex, Framingham, MA USA) with MS/MSALL with SWATH TM Acquisition MicroApp (Sciex). ProteinPilot software (v5.0 ABSciex). Mrkerview 1.3.1 (Sciex, Framingham, MA USA).
Data analysis	GraphPad Prism V9.3.1 (Statistics and outlier removal using iterative Grubb's using Alpha = 0.2). R Project for Statistical Computing V4.1.0 with function "normalize.quantiles". and using R package limma for statistical analysis. Correction for multiple testing was applied using the method of Benjamini & Hochverg. FIJI - ImageJ V.2,1,0/1.53c

For manuscripts utilizing custom algorithms or software that are central to the research but not yet described in published literature, software must be made available to editors and reviewers. We strongly encourage code deposition in a community repository (e.g. GitHub). See the Nature Portfolio guidelines for submitting code & software for further information.

Data

Policy information about availability of data

All manuscripts must include a data availability statement. This statement should provide the following information, where applicable:

- Accession codes, unique identifiers, or web links for publicly available datasets
- A description of any restrictions on data availability
- For clinical datasets or third party data, please ensure that the statement adheres to our policy

Authors can confirm that all relevant data supporting the findings of this study are available within the article and its supplementary information fles.

Field-specific reporting

Please select the one below that is the best fit for your research. If you are not sure, read the appropriate sections before making your selection.

☒ Life sciences ☐ Behavioural & social sciences ☐ Ecological, evolutionary & environmental sciences

For a reference copy of the document with all sections, see nature.com/documents/nr-reporting-summary-flat.pdf

Life sciences study design

All studies must disclose on these points even when the disclosure is negative.

Sample size	No information was provided. Provided the difference in the expression levels of aSyn between WT mice and Thy1-aSyn mice (2 times more), a very small number of animals is required to compare between these groups (as determined by power analysis). In particular, For an induction of aSyn accumulation due to MGO in Thy1-aSyn mice, expected of 1.2-fold, a power analysis indicates that 6 animals per group are required for a Power of 95% with a p value of 0.01, or 5 animals per group for a Power of 90% and p value of 0.01. For that reason, a minimal number of 5 mice were included per group.
Data exclusions	For all behavioral and biochemical analysis, all data was included. For Mass spectrometry data, 5 independent measurements of 5 different animals were analyzed. Outlier removal using iterative Grubb's using Alpha = 0.2 was used, as described in the manuscript.
Replication	Pilot study was performed for animal behaviour analysis. Data were replicated. Biochemistry analysis was replicated.
Randomization	The Thy1-aSyn and WT littermates were randomly divided in the sub-groups submitted to Vehicle or MGO injection. Behavioral experiments were also blinded and randomized.
Blinding	Blinded behavioral experimentation was performed. TH- and NeuN-positive nerons counting was blinded for experimental grouping.

Reporting for specific materials, systems and methods

We require information from authors about some types of materials, experimental systems and methods used in many studies. Here, indicate whether each material, system or method listed is relevant to your study. If you are not sure if a list item applies to your research, read the appropriate section before selecting a response.

Materials & experimental systems

n/a	Involved in the study	
☐	☒	Antibodies
☒	☐	Eukaryotic cell lines
☒	☐	Palaeontology and archaeology
☐	☒	Animals and other organisms
☒	☐	Human research participants
☒	☐	Clinical data
☒	☐	Dual use research of concern

Methods

n/a	Involved in the study	
☒	☐	ChIP-seq
☒	☐	Flow cytometry
☒	☐	MRI-based neuroimaging

Antibodies

Antibodies used	Proteins were probed using Nε-(carboxyethyl)lysine (CEL) (Mouse Anti- Nε-carboxyethyl lysine, Cosmo-Bio, USA), aSyn (Purified Mouse Anti-α-Synuclein antibody, BD Biosciences; San Jose, CA, USA), phosphorylated aSyn at residue 129 (pS129, [J18] SMC-600, StressMarq; Victoria, Canada), glyoxalase I (Rabbit polyclonal anti-Glyoxalase I antibody – FL-184, sc67351, Santa Cruz Biotechnology) and β-actin (Mouse Monoclonal anti-β-actin antibody, Ambion, Thermo Fisher Scientific; Waltham, MA, USA).anti-tyrosine hydroxylase (TH) rabbit (Millipore, 1:1000), anti-aSyn (1:1000, BD Transduction laboratories), anti-NeuN mouse (Millipore, 1:400)
Validation	All antibodies are widely validated by us and by several researchers. These are the most used antibodies in the field

Animals and other organisms

Policy information about studies involving animals; ARRIVE guidelines recommended for reporting animal research

Laboratory animals	Transgenic mice overexpressing human aSyn under the Thy-1 promoter were generated on a mixed C57BL/6-DBA/2 background as described previously.
Wild animals	Animals were obtained from our breeding colony on this background by breeding mutant females with wildtype (WT) C57BL/6-DBA/2 males. Offspring were genotyped via polymerase chain reaction (PCR) amplification analysis of DNA extracted from ear or toe. PCR

	was performed using the following primers: Thy-1- F: 5′-CTG GAA GAT ATG CCT GTG GA-3′, Thy-1-R: 5′-GAG GAA GGA CCT CGA GGA AT-3′, with an annealing temperature of 60oC and 40 cycles of amplification as previously.
Field-collected samples	*For laboratory work with field-collected samples, describe all relevant parameters such as housing, maintenance, temperature, photoperiod and end-of-experiment protocol OR state that the study did not involve samples collected from the field.*
Ethics oversight	Animal procedures were carried out in accordance with the European Community guidelines (Directive 2010/63/EU), Portuguese law on animal care (DL 113/2013), and approved by the iMM Internal Committee and the Portuguese Animal Ethics Committee (Direcção Geral de Alimentação e Veterinária - DGAV).

Note that full information on the approval of the study protocol must also be provided in the manuscript.
